# Measuring multimorbidity in research: Delphi consensus study

**DOI:** 10.1136/bmjmed-2022-000247

**Published:** 2022-07-27

**Authors:** Iris S S Ho, Amaya Azcoaga-Lorenzo, Ashley Akbari, Jim Davies, Kamlesh Khunti, Umesh T Kadam, Ronan A Lyons, Colin McCowan, Stewart W Mercer, Krishnarajah Nirantharakumar, Sophie Staniszewska, Bruce Guthrie

**Affiliations:** 1 Usher Institute, University of Edinburgh, Edinburgh Medical School, Edinburgh, UK; 2 Bute Medical School, University of St Andrews, St Andrews, UK; 3 Swansea University Medical School, Swansea University, Swansea, UK; 4 Department of Computer Science, University of Oxford, Oxford, UK; 5 Department of Health Sciences, University of Leicester, Leicester, UK; 6 Health Data Research UK, Swansea University, Swansea, UK; 7 Public Health, University of Birmingham, Birmingham, UK; 8 Division of Health Sciences, University of Warwick, Coventry, UK

**Keywords:** epidemiology, primary health care, public health, research design, medicine

## Abstract

**Objective:**

To develop international consensus on the definition and measurement of multimorbidity in research.

**Design:**

Delphi consensus study.

**Setting:**

International consensus; data collected in three online rounds from participants between 30 November 2020 and 18 May 2021.

**Participants:**

Professionals interested in multimorbidity and people with long term conditions were recruited to professional and public panels.

**Results:**

150 professional and 25 public participants completed the first survey round. Response rates for rounds 2/3 were 83%/92% for professionals and 88%/93% in the public panel, respectively. Across both panels, the consensus was that multimorbidity should be defined as two or more long term conditions. Complex multimorbidity was perceived to be a useful concept, but the panels were unable to agree on how to define it. Both panels agreed that conditions should be included in a multimorbidity measure if they were one or more of the following: currently active; permanent in their effects; requiring current treatment, care, or therapy; requiring surveillance; or relapsing-remitting conditions requiring ongoing care. Consensus was reached for 24 conditions to always include in multimorbidity measures, and 35 conditions to usually include unless a good reason not to existed. Simple counts were preferred for estimating prevalence and examining clustering or trajectories, and weighted measures were preferred for risk adjustment and outcome prediction.

**Conclusions:**

Previous multimorbidity research is limited by inconsistent definitions and approaches to measuring multimorbidity. This Delphi study identifies professional and public panel consensus guidance to facilitate consistency of definition and measurement, and to improve study comparability and reproducibility.

**WHAT IS ALREADY KNOWN ON THIS TOPIC**

- How multimorbidity is defined and measured in research studies varies widely
- Previous consensus studies have focused on choice of conditions to include in multimorbidity measures, and have usually involved only local or regional professional panels

**WHAT THIS STUDY ADDS**

- This study provides guidance on how to define and measure multimorbidity in research studies, based on Delphi consensus in professional and public panels; although consensus was reached that multimorbidity should be defined as two or more long term conditions, none was reached on alternative definitions of complex multimorbidity
- Panels agreed on which conditions to always include and which to usually include in multimorbidity measurement
- Panels also agreed that simple counts of conditions were preferred or considered acceptable for studies estimating prevalence, identifying and counting disease clusters, and exploring trajectories of multimorbidity over time, and that weighted measures were for assessing severity of disease burden, and risk adjustment or outcome prediction

**HOW THIS STUDY MIGHT AFFECT RESEARCH, PRACTICE, OR POLICY**

- The consensus list of conditions to always and usually include in multimorbidity measurement provides a core set for researchers to use to improve comparability and replicability, although researchers can add other conditions relevant to local context and purpose
- Consensus about when weighted measures or simple counts were preferred depending on the purpose of an analysis provides a guide to inform researchers choice of methods
- Further research is needed to better define and demonstrate the value of concepts such as complex multimorbidity

## Introduction

In many regions of the world, a growing proportion of adults has multiple long term conditions or multimorbidity.^1–3^ Multimorbidity is defined as the coexistence of two or more long term conditions.^4^ Multimorbidity prevalence increases substantially with age, and is the norm in people aged 65 years or older.^5–7^ Prevalence is also higher in less affluent and less well educated groups,^6 7^ with multimorbidity also occurring at younger ages in these groups.^1 5^ About 30-40% of people with multimorbidity have both a physical and a mental health condition,^5 6^ with multimorbidity involving a combination of physical and mental health being more common in women, and less affluent and less well educated individuals.^5 6^

Despite broad agreement that multimorbidity should be defined as the presence of two or more chronic conditions, no international consensus exists on how to operationalise this broad definition in measures used in research. Multimorbidity measures vary widely in terms of the number, labelling, type, and severity of included conditions or groups of conditions.^4^ Without common definitions, many different tools have been developed and used to measure multimorbidity. The tools commonly used in research and clinical practice include: simple (unweighted) disease counts, weighted disease counts, and weighted medication counts.^8^ In addition, many different weighting schemes have been applied to serve different purposes.

Consequently, comparing and reproducing studies is difficult, with for example, large variation in estimates of the prevalence of multimorbidity in different studies, ranging from 3.5% to 100%.^9^ The high level of heterogeneity in multimorbidity prevalence has been found to be mainly attributed to age and inconsistent multimorbidity measurement.^10^ The estimated pooled prevalence was 68.7% for an oldest population (aged ≥74 years), 26.3% for a younger population (aged >55 years), 29.3% for a measure including fewer than nine conditions, and 87.6% for a measure including 44 or more conditions.^10^

Previous studies have synthesised existing evidence on multimorbidity measures,^8 11 12^ compared the performance of different measures in predicting selected outcomes,^13 14^ and adapted existing measures to meet the professionally perceived needs of specific regions or populations.^15 16^ These studies have identified heterogeneity in the definition and measurement of multimorbidity as a key issue or limitation, and demonstrate the need for shared approaches to definition to improve comparability and reproducibility. In addition, little attention has been given to directly involving patients and the public in the discussion of multimorbidity definition and measurement. Therefore, this study aimed to explore views and develop consensus on how to measure multimorbidity using a modified Delphi study with an international panel of professionals and the public.

## Methods

The overall study design was a modified Delphi method with two international panels of professionals and of members of the public.^17^ We used this method as a group consensus strategy to systematically and iteratively explore opinions of professionals and public contributors, and develop consensus on methods of defining and measuring multimorbidity. The study protocol is provided in online supplemental appendix 1.

10.1136/bmjmed-2022-000247.supp1 Supplementary data

### Data collection methods

Data were collected in three rounds of online questionnaires sent to each individual member of the panels between 30 November 2020 and 18 May 2021. Core questions were the same for both panels, but some more technical questions were only asked of one panel (eg, questions about the acceptability of simple counts or weighted measures for different research purposes were only asked of the professional panel). In the second and third rounds, participants were fed back a summary of all responses to inform their judgments.^17 18^

Round 1 questions were informed by the findings of a recent systematic review,^19^ which identified the characteristics of multimorbidity measures used in research in relation to the study purposes. Each questionnaire included both closed (Likert scaled) questions and open ended questions. Depending on the question, participants were asked to rate (from strongly agree to strongly disagree) or rank (the importance of statements on a scale of 1-5) items or statements using Likert scales.^17^ The open ended responses were triangulated with close ended responses, and the results were used to develop new items in the following rounds. Second and third round items were a mix of those scored in the previous round that did not achieve consensus, and new items based on open ended responses in previous rounds. The interactive and repetitive survey rounds, as part of standard Delphi methods, were to improve the framing of the statements for panellists, attest their responses through the iterative process, and achieve consensus. All questionnaires are provided in online supplemental appendix 2.

10.1136/bmjmed-2022-000247.supp2 Supplementary data

To conceptualise multimorbidity, eight aspects were explored in the Delphi surveys (online supplemental appendices 2 and 3): the cut-off number of conditions for defining multimorbidity (and complex multimorbidity), duration of a condition for it to be defined as long term, types of conditions to include (eg, medical diagnoses, risk factors, and health behaviours), categorisation of conditions, choice of conditions based on their impact, data sources, which conditions to include (eg, name of individual conditions), and choice of simple counts versus weighted measures for different purposes.

10.1136/bmjmed-2022-000247.supp3 Supplementary data

### Participants

Participants recruited to the professional panel were clinicians with experience of caring for patients with multiple long term conditions; and researchers and policy makers with an interest in multimorbidity. Participants recruited to the public panel were members of the public with multiple long term conditions or an interest in multimorbidity.

We identified participants using a range of methods: publicly available information including published work, publicly available websites, reports, and policy documents (to identify healthcare professionals, policy makers, or public participants for example, in guideline development). For the public panel, we asked conveners of patient and public involvement groups to forward the invite to their members, and asked participants (and potential participants) to forward study information to others who might meet the criteria, directly or via social media (snowball sampling). No direction on the number of participants is required for a Delphi survey.^17^ To provide representative information, some studies have involved more than 60 experts, while others involved as few as 15.^18^ In this Delphi study, we aimed to recruit a minimum number of experts and public contributors of 25-30, but we had no maximum limit.

### Minimising bias and data analysis

We used several techniques to minimise sampling and non-response bias.^20^ These techniques included sampling expert panellists with different study interests in the field of multimorbidity, using multiple survey distribution methods to increase response rates, highlighting the match between the survey and participant interests, identifying any differences in personal characteristics of those who did or did not complete the surveys, collecting multiple waves of data, and ensuring anonymity among panellists to facilitate open and truthful discussion about their views.

Descriptive statistics were used to describe participants’ personal characteristics and responses to statements in three rounds of surveys (including frequency, percentage, median, and interquartile range). Before any data collection, we prespecified consensus as ≥70% of panellists providing the same response.^17 21^

For items relating to multimorbidity definition, any statements that reached consensus (to "strongly agree," "strongly disagree," "very important," and "not important at all"; rated on a scale of 1-5) in the initial round would not be asked again in the next rounds. If no consensus was reached, then questions were asked again in the following rounds. If statements did not reach consensus in all rounds, we examined for any consensus in terms of "agree" (the sum of strongly agree and agree), "disagree" (the sum of strongly disagree and disagree), "sufficiently important" (the sum of very important and sufficiently important), or "not important" (the sum of not important at all and slightly important) in the final round (online supplemental figure S1). "Don’t know" responses were excluded from the denominator when calculating percentages.

For questions related to the choice of conditions to include in multimorbidity measures, we first identified whether consensus was reached to always include a condition (≥70% agreeing) in multimorbidity measurement. If no consensus was reached, we identified any agreement (≥70%) to usually include unless a good reason to exclude in a particular context (referred to here as "usually include"), defined as the sum of responses to "always include" and "usually include."

For the choice of conditions to include in measures, we included all conditions as "always include" if either panel rated it as "always" and the other rated it as "usually." If one panel rated a condition as "usually include" and the other did not, we used the Rasch dichotomised model as a sensitivity analysis to examine items (conditions) being endorsed (rated always or usually include) and unendorsed (not rated always or usually include) by all participants (online supplemental box 1; this analysis was not prespecified).^22^ The level of endorsement was estimated on the basis of the item difficulty parameter in the Rasch model, with negative values representing more frequently endorsed and positive values representing less frequently endorsed.^23^ Conditional maximum likelihood estimation in the Rasch analysis was used to produce consistent item parameter estimates without assuming a specific population distribution for the latent trait.^24^ In the face of disagreement between panels (ie, one panel saying "usually include," the other not), we rated conditions as "usually include" if the item difficulty parameter was ≤0.5.^25^ All statistical analyses were conducted using R version 4.0.4.

### Patient and public involvement

A member of our research team (SS) organised an online meeting with a public reference group in September 2020 to discuss the development and design of the first Delphi questionnaires. Feedback provided by the public reference group included use of simple terms to describe medical diagnosis, and clarity about the difference between multimorbidity and comorbidity and questions relating to weighting. Based on the feedback, we therefore incorporated a short description explaining each medical diagnosis and inserted a two page document introducing the study topic in the online questionnaires. With the support of Health Data Research UK and our colleagues, several members of the public took part in the Delphi study to provide their views on how multimorbidity should be defined and measured. Subsequent round questionnaires were modified in response to comments and suggestions from all panellists including the public. All participants were sent a summary of the findings after completion of data analysis.

## Results

**Figure 1:**
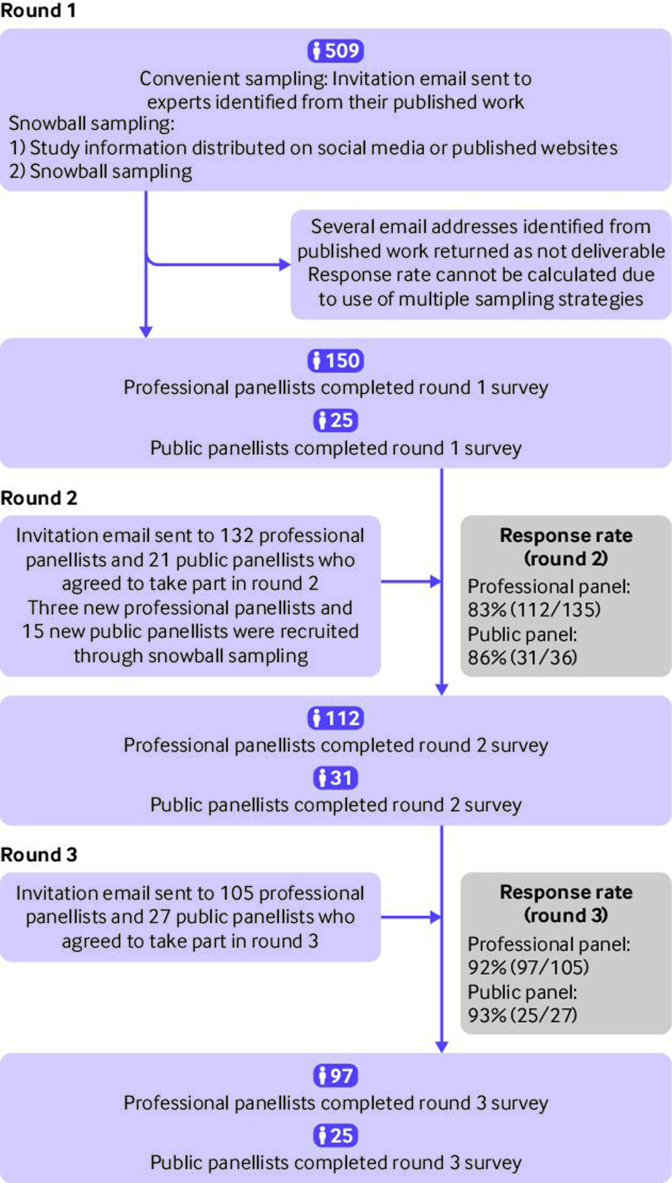
Process of participant recruitment

In round 1, 150 professional panellists and 25 public panellists took part in the survey (figure 1). Owing to the use of multiple sampling strategies, the response rate in round 1 could not be estimated. The response rates for rounds 2 and 3 in the professional panel were 83% (112/135) and 92% (97/105), respectively, and 86% (n=31/36) and 93% (25/27) in the public panel, respectively. The number of participants in round 2 increased because of snowballing sampling (figure 1). Characteristics of respondents and non-respondents were similar across the three rounds in the professional panel and the public panel (table 1 and online supplemental table S1).

**Table 1:** Personal characteristics of participants who responded to Delphi surveys on multimorbidity measurement

Characteristics	Professional panellists			Public panellists		
	Round 1 (n=150)	Round 2 (n=112)	Round 3 (n=97)	Round 1 (n=25)	Round 2 (n=31)	Round 3 (n=25)
Continent						
Europe	80 (53.3)	59 (52.7)	50 (51.5)	21 (84.0)	30 (96.8)	24 (96.0)
North America	31 (20.7)	24 (21.4)	22 (22.7)	1 (4.0)	0	0
Australasia	13 (8.7)	12 (10.7)	10 (10.3)	0	0	0
Asia	20 (13.3)	12 (10.7)	11 (11.3)	1 (4.0)	0	0
South America	5 (3.3)	4 (3.6)	4 (4.1)	1 (4.0)	1 (3.2)	1 (4.0)
Africa	1 (0.6)	1 (0.9)	0	0	0	0
Not stated	0	0	0	1 (4.0)	0	0
Country income						
High income	139 (92.7)	103 (92.0)	89 (91.8)	22 (88.0)	30 (96.8)	24 (96.0)
Low and middle income	11 (7.3)	9 (8.0)	8 (8.2)	2 (8.0)	1 (3.2)	1 (4.0)
Not stated	0	0	0	1 (4.0)	0	0
Multimorbidity of participant						
Yes	17 (11.3)	16 (14.3)	13 (13.4)	13 (52.0)	19 (61.3)	17 (68.0)
No	133 (88.7)	95 (84.8)	83 (85.6)	12 (48.0)	12 (38.7)	8 (32.0)
Not stated	0	1 (0.9)	1 (1.0)	0	0	0
Multimorbidity of participant's family or friends						
Yes	104 (69.3)	76 (67.9)	66 (68.0)	17 (68.0)	24 (77.4)	20 (80.0)
No	46 (30.7)	35 (31.2)	30 (30.9)	8 (32.0)	7 (22.6)	5 (20.0)
Not stated	0	1 (0.9)	1 (1.0)	0	0	0
Area of work*						
Research	123 (82.0)	94 (83.9)	81 (83.5)	—	—	—
Public policy	13 (8.7)	9 (8.0)	7 (7.2)	—	—	—
Clinical practice	58 (38.7)	43 (38.4)	39 (40.2)	—	—	—
Teaching	4 (2.7)	3 (2.7)	4 (4.1)	—	—	—
Main work setting						
Government	10 (6.7)	6 (5.4)	6 (6.2)	—	—	—
Academia	95 (63.3)	70 (65.5)	57 (58.8)	—	—	—
Hospital	18 (12.0)	16 (14.3)	16 (16.5)	—	—	—
Primary care	25 (16.7)	16 (14.3)	14 (14.4)	—	—	—
Other	2 (13.3)	4 (3.6)	4 (4.1)	—	—	—
Populations of interest*						
General population	100 (66.7)	80 (71.4)	69 (71.1)	—	—	—
Older people	98 (65.3)	67 (59.8)	60 (61.9)	—	—	—
Middle aged and older	89 (59.3)	59 (52.7)	53 (54.6)	—	—	—
Socially deprived	85 (56.7)	62 (55.4)	56 (57.7)	—	—	—
Women†	37 (24.7)	33 (29.5)	31 (32.0)	—	—	—
Men†	33 (22.0)	27 (24.1)	26 (26.8)	—	—	—
Children	19 (12.7)	17 (15.2)	16 (16.5)	—	—	—
Ethnic group/indigenous	—	43 (38.4)	40 (41.2)	—	—	—
People with disability	—	46 (41.2)	44 (45.4)	—	—	—
Age group (years)						
18-34	—	—	—	2 (8.0)	1 (3.2)	1 (4.0)
35-54	—	—–	—	6 (24.0)	7 (22.6)	5 (20.0)
55-64	—	—–	—	5 (20.0)	7 (22.6)	6 (24.0)
≥65	—	—	—	12 (48.0)	16 (51.6)	13 (52.0)
Sex						
Female†	—	—	—	14 (56.0)	19 (61.3)	14 (56.0)
Male†	—	—	—	11 (44.0)	12 (38.7)	11 (44.0)

Data are number (%) of participants.

*Participants could choose more than one response so percentages can sum to >100%.

†Female and male refers to the sex of the public panellists; women and men refer to the populations that the professional panellists have research interests in.

In the professional panel in round 1 (table 1), 53.3% of panellists were from Europe and 20.7% from North America with smaller proportions from Australasia (8.7%), Asia (13.3%), South America (3.3%), and Africa (0.6%). Most professional panellists were interested in multimorbidity in the general population or in middle aged or older adults, but only 12.7% were interested in multimorbidity in children. More than half of professional panellists were interested in multimorbidity in socially deprived populations (56.7%), and 38.0% in multimorbidity in ethnic minority and indigenous groups. In the public panel, most panellists were from Europe, with fewer than 4% from Asia, North America, or South America. Just over half of public panellists were women (56.0%), and 48.0% of the public panellists were aged 65 years and older. The proportions of participant characteristics were similar across rounds.

Both panels agreed that multimorbidity should be defined as the co-occurrence of two or more long term conditions. Defining complex multimorbidity was considered useful by more than 80% of both panels, with consensus in the public panel that complex multimorbidity could be defined as the co-occurrence of three or more long term conditions. However, no consensus in the professional panel was reached on how to define complex multimorbidity with variation in whether three or more conditions had to come from any, at least two, or at least three body systems. Neither panel agreed on the value of any other patterns of complex multimorbidity, with physical-mental comorbidity chosen by 33% of professional panellists and 44% of public panellists, physical functional limitations by 30.9% of professional panellists and 32% of public panellists, difficulties in managing illness due to social factors by 26.8% of professional panellists and 28% of public panellists, and frailty by 25.8% of professional panellists and 12% of public panellists (online supplemental table S2).

Conditions were considered to be long term if they persisted for six months or more in the professional panel (70.5%); conditions were considered long term if they lasted 12 months or more in the public panel (76.0%). More than 95% of panellists from both panels would include formal medical diagnoses in multimorbidity measurement. While the public panel agreed that clinical risk factors were important for multimorbidity measurement (74.2%) (online supplemental table S2), the professional panel did not reach a consensus. Symptoms, health behaviour, health impacts, social deprivation, and consequences of treatment did not reach consensus in both panels as conditions to include for measurement. Both panels agreed that conditions should be included in a multimorbidity measure if they were any of the following: currently active; permanent in their effects; requiring current treatment, care, or therapy; requiring surveillance (including treated cancers that require surveillance); or relapsing-remitting conditions that require ongoing treatment, care, or therapy (online supplemental table S3). On the other hand, no consensus was reached on the conditions that might recur or remit but happen rarely and that usually require treatment or therapy at some point in the future even if not currently treated. Both panels reached consensus that studies should count individual conditions rather than categories defined by body system, and that disease complications should be counted separately from diseases (eg, peripheral neuropathy and diabetes). The public panel (but not the professional panel) agreed that individual cancers should be counted separately (table 2 and online supplemental table S2).

**Table 2:** Responses to questions relevant to definitions of multimorbidity and complex multimorbidity. Data are percentage of panellists agreeing (and Delphi survey round (R))

Question or statement	Professional panellists	Public panellists
Definition of multimorbidity		
Multimorbidity is two or more long term conditions	84.8 (R2)	88.0 (R1)
Complex multimorbidity is a useful idea	87.5 (R2)	84.0 (R2)
Complex multimorbidity is three or more long term conditions	No consensus	76.0 (R3)
Types of conditions to include		
Long term means present for six months or more	70.5 (R2)	No consensus
Long term means present for 12 months or more	No consensus	76.0 (R1)
Medical diagnoses	99.1 (R2)	96.8 (R1)
Clinical risk factors	No consensus	74.2 (R2)
Currently active	98.7 (R1)	93.5 (R2)
Permanent in their effects	98.6 (R1)	96.0 (R1)
Requiring current treatment, care, or therapy	100.0 (R2)	96.8 (R2)
Requiring surveillance	74.7 (R3)	88.0 (R3)
Remitting-relapsing conditions requiring ongoing treatment or care	93.8 (R3)	92.0 (R3)
Counting or categorisation		
Count individual conditions not broad disease categories	72.0 (R1)	88.0 (R1)
Count individual cancers separately	No consensus	76.0 (R1)
Criteria for selecting conditions relating to impact		
Significantly increase risk of death	94.6 (R2)	100 (R1)
Significantly reduce quality of life	96.6 (R1)	93.5 (R2)
Cause frailty	89.9 (R2)	90.3 (R2)
Cause physical disability	93.3 (R1)	96.8 (R2)
Significantly worsen mental health	92.6 (R1)	87.1 (R2)
Significantly worsen self-perceived health status	77.4 (R2)	No consensus
Significantly increase treatment burden	87.4 (R2)	87.1 (R2)
Impacted by social deprivation and poverty	No consensus	74.2 (R2)
Data source		
Conditions should be the same/similar in both self-report and clinical/administrative database studies	71.8 (R2)	96.0 (R2)
Purposes where a simple count preferred or acceptable*		
Estimating prevalence	83.7 (R3)	Not asked
Identifying and counting disease clusters	80.2 (R3)	Not asked
Exploring trajectories of multimorbidity	72.7 (R3)	Not asked
Purposes where weighted measure preferred or acceptable*		
Assessing severity of disease burden	94.5 (R3)	Not asked
Risk adjustment or outcome prediction	91.2 (R3)	Not asked
Outcomes important to weight against		
Death	92.8 (R1)	96.8 (R2)
Healthcare use	83.7 (R1)	90.3 (R2)
Health related quality of life	92.3 (R1)	90.3 (R2)
Physical disability	87.8 (R1)	86.7 (R2)
Frailty	86.3 (R1)	76.7 (R2)

Absolute numbers for percentage data are as follows: professional panel, round 1 n=150, round 2 n=112, round 3 n=97; public panel, round 1 n=25, round 2 n=31, round 3 n=25.

*Panellists could either state that they preferred a simple or weighted measure for the listed options, or that a simple or weighted measure were both acceptable; values are the sum of "preferred" or "acceptable."

In respect to criteria for selecting conditions based on impact, more than 70% of both panels agreed that conditions were appropriate to include in multimorbidity measurement if they were any of the following: significantly reduce quality of life, significantly worsen mental health, significantly increase risk of death, cause frailty, cause physical disability, or significantly increase treatment burden. The professional panel (but not the public panel) reached consensus on including conditions that significantly worsen self-perceived health status. The public panel (but not the professional panel) reached consensus on including conditions that are affected by social deprivation and poverty (table 2 and online supplemental table S4). Both panels agreed that conditions included for measurement should be similar in self-report, administrative databases, and medical records.

Technical questions about the use of simple counts versus weighted measures based on study purposes were only asked in the professional panel. In round 1, no consensus was reached on whether simple counts or weighted measures were generally preferable (online supplemental table S5). In rounds 2 and 3, for a range of different purposes, professionals were asked if they preferred simple counts or weighted measures or if either was acceptable. There was no consensus that one or other type of measure was preferred for any of the purposes asked, but for all but one purpose, there was clear consensus that one type of measure was preferred or acceptable (table 2 and online supplemental table S6). Simple counts were preferred or acceptable for estimating the prevalence of multimorbidity, identifying and counting disease clusters, and exploring trajectories of multimorbidity. Weighted measures were preferred or acceptable for assessing the severity of disease burden, risk adjustment, and outcome prediction (in general) and for every specific outcome asked about (online supplemental table S7). No consensus was reached on the best type of measure for exploring or identifying predictors of multimorbidity. In round 2, 21.7% (n=20) of panellists preferred to use weighted indices, 46.7% (n=43) preferred to empirically derive weights based on the individual impact of diseases on outcome (eg, regression models to calculate weights), and 26.1% (n=24) preferred to set rules based on level of severity to grade each condition (eg, having presence of a condition=1 point, treatment=additional 1 points, functional limitation=additional 1 point). In both professional and public panels, mortality, healthcare use, health related quality of life, physical disability, and frailty were rated as sufficiently important or very important to weight against by ≥70% panellists if weighted measures were preferred.

Of the 107 individual conditions asked about in the Delphi questionnaires (online supplemental file 2), 24 were rated as "always include" in multimorbidity measurement (the 107 conditions were defined on the basis of results of a recent systematic review^19^ and panellists’ suggestions in initial rounds). This "always include" list consisted of 16 conditions (table 3) that reached consensus in both professional and public panels (end stage kidney disease, heart failure, dementia, chronic liver disease, chronic kidney disease, stroke, solid organ cancers, metastatic cancers, haematological cancers, multiple sclerosis, Parkinson’s disease, coronary artery disease, cystic fibrosis, epilepsy, diabetes, and HIV/AIDS), seven conditions reaching consensus in the professional (but not public) panel (chronic obstructive pulmonary disease, inflammatory bowel disease, connective tissue disease, paralysis, schizophrenia, peripheral artery disease, and asthma), and one condition reaching consensus in the public (but not professional) panel (Addison’s disease; online supplemental tables S8 and S9).

**Table 3:** Conditions with consensus to always include and usually include unless there is a specific reason not to in a multimorbidity measure, by panel, based on Delphi surveys. Data are percentage of panellists agreeing (and Delphi survey round (R)) unless stated otherwise

Condition	Always include condition		Usually include condition*		Difficulty parameter estimate in both panels (logit)†
	Professional panellists	Public panellists	Professional panellists	Public panellists	
Heart failure	90.0 (R1)	83.9 (R2)	—	—	−3.1
Chronic liver disease	88.5 (R1)	80.6 (R2)	—	—	−3.5
Diabetes	87.3 (R1)	71.0 (R2)	—	—	−3.5
Parkinson’s disease	86.6 (R1)	77.4 (R2)	—	—	−2.8
End stage kidney disease	86.4 (R1)	90.3 (R2)	—	—	−2.0
Coronary artery disease	82.7 (R1)	74.2 (R2)	—	—	−2.6
Dementia	82.6 (R1)	83.3 (R2)	—	—	−2.3
Multiple sclerosis	80.7 (R1)	77.4 (R2)	—	—	−1.9
Stroke	80.0 (R1)	80.6 (R2)	—	—	−2.6
Chronic kidney disease	79.3 (R1)	80.6 (R2)	—	—	−2.8
HIV/AIDS	78.5 (R1)	71.0 (R2)	—	—	−1.5
Metastatic cancers	77.4 (R1)	70.8 (R1)	—	—	−1.3
Haematological cancers	77.2 (R1)	70.8 (R1)	—	—	−1.9
Solid organ cancers	76.5 (R1)	70.8 (R1)	—	—	−2.0
Cystic fibrosis	75.8 (R1)	74.2 (R2)	—	—	−1.3
Epilepsy	73.0 (R1)	71.0 (R2)	—	—	−2.2
Chronic obstructive pulmonary disease	85.9 (R1)	No consensus	—	96.8 (R2)	−3.1
Inflammatory bowel disease	82.6 (R1)	No consensus	—	100 (R2)	−1.9
Connective tissue disease	79.7 (R1)	No consensus	—	93.3 (R2)	−2.3
Paralysis (other than stroke)	76.0 (R1)	No consensus	—	93.3 (R2)	−0.9
Schizophrenia	75.2 (R1)	No consensus	—	93.5 (R2)	−1.6
Peripheral arterial disease	71.1 (R1)	No consensus	—	96.8 (R2)	−1.6
Asthma	70.7 (R1)	No consensus	—	80.6 (R2)	−1.1
Addison’s disease	No consensus	70.8 (R2)	86.9 (R2)	—	−0.8
Depression	—	—	92.9 (R2)	77.4 (R2)	−0.9
Heart valve disorders	—	—	92.0 (R2)	100 (R2)	−1.6
Bipolar disorder	—	—	90.0 (R2)	93.5 (R2)	−1.1
Melanoma	—	—	88.2 (R2)	100 (R2)	−1.1
Bronchiectasis	—	—	86.7 (R3)	88.0 (R3)	−0.7
Osteoarthritis	—	—	84.7 (R2)	87.1 (R2)	−0.5
Pancreatic disease	—	—	84.4 (R2)	96.7 (R2)	−0.7
Arrhythmia	—	—	83.9 (R2)	85.7 (R2)	−0.4
Thyroid disorders	—	—	82.7 (R2)	87.1 (R2)	−0.3
Venous thrombotic disease	—	—	82.4 (R2)	96.8 (R2)	−0.5
Drug or alcohol misuse	—	—	81.8 (R2)	74.2 (R2)	−0.02
Anaemia	—	—	81.7 (R2)	96.7 (R2)	−0.4
Chronic Lyme disease	—	—	81.3 (R3)	79.2 (R3)	−0.03
Transient ischaemic attack	—	—	80.4 (R2)	96.8 (R2)	−0.4
Treated cancer requiring surveillance	—	—	79.3 (R3)	80.0 (R3)	0.01
Eating disorders	—	—	79.1 (R2)	74.2 (R2)	0.2
Vision impairment that cannot be corrected	—	—	78.6 (R2)	74.2 (R2)	0.1
Long term musculoskeletal problems due to injury	—	—	78.4 (R3)	70.8 (R3)	0.2
Tuberculosis	—	—	82.4 (R2)	90.3 (R2)	0.07
Endometriosis	—	—	75.7 (R2)	89.3 (R2)	0.1
Chronic primary pain	—	—	75.3 (R3)	80.0 (R3)	0.2
Hearing impairment that cannot be corrected	—	—	73.9 (R2)	74.2 (R2)	0.4
Peptic ulcer	—	—	73.9 (R2)	83.9 (R2)	0.3
Post-traumatic stress disorder	—	—	73.4 (R2)	74.2 (R2)	0.5
Post-acute covid-19	—	—	73.4 (R3)	92.0 (R3)	0.2
Benign cerebral tumours	—	—	73.3 (R3)	76.0 (R3)	0.4
Peripheral neuropathy	—	—	73.1 (R2)	96.7 (R2)	0.1
Hypertension (untreated)	—	—	73.0 (R2)	71.0 (R2)	0.4
Congenital disease and chromosomal abnormalities	—	—	72.6 (R2)	90.0 (R2)	0.2
Chronic urinary tract infection	—	—	71.8 (R2)	86.7 (R2)	0.3
Aneurysm	—	—	71.6 (R3)	96.8 (R2)	0.5
Meniere’s disease	—	—	71.3 (R2)	71.0 (R2)	0.5
Osteoporosis	—	—	70.3 (R2)	80.6 (R2)	0.5
Autism	—	—	70.1 (R2)	87.1 (R2)	0.5
Hypertension (treated)	—	—	80.4 (R2)	No consensus	0.3
Anxiety	—	—	80.0 (R2)	No consensus	0.4
Gout	—	—	76.1 (R2)	No consensus	0.4

*Consensus of conditions to usually include is defined as more than 70% of panellists rated conditions as always include or usually include.

†Used to examine endorsement (see also online supplemental table S14).

Of 37 conditions rated to usually include unless a good reason to exclude in a particular context, 34 reached consensus in both panels (table 3, online supplemental tables S10 and S11). Of the 22 conditions that reached consensus to usually include in only one panel, three conditions (treated hypertension, gout, and anxiety) had an estimated difficulty parameter ≤0.5, and were therefore considered to be in the "usually include" list (online supplemental table S12). Twenty seven conditions did not reach consensus to include in either panel, but no condition was rated as "usually exclude" or "always exclude" (online supplemental table S12).

Endorsement did not vary by participant characteristics apart from attention deficit hyperactivity disorder, which did not reach consensus in both panels, but was substantially more endorsed by professional panellists interested in multimorbidity in children than those who were not (online supplemental table S13).

## Discussion

### Principal findings

**Figure 2:**
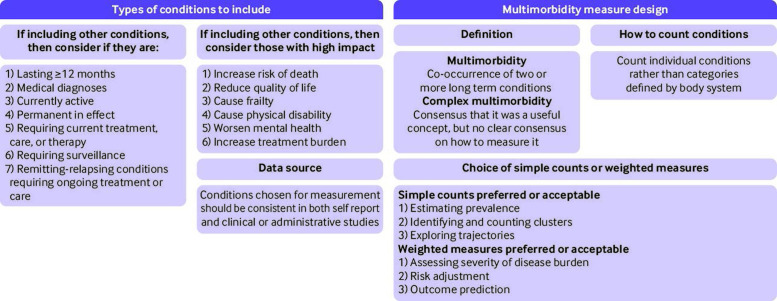
Summary of findings and recommendations on multimorbidity definition. Professional panel consensus was >6 months; patient panel consensus was >12 months

**Figure 3:**
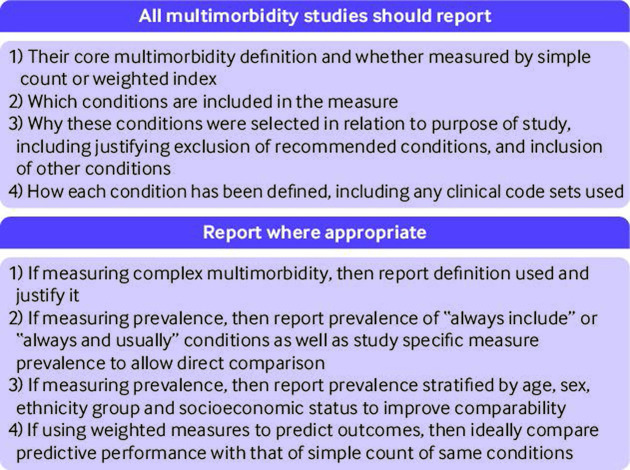
Reporting recommendations on multimorbidity

Figure 2 and figure 3 summarise the research and reporting recommendations, and table 4 lists the conditions recommended for inclusion in multimorbidity measures. This consensus study found that more than 70% of professional and public panellists defined multimorbidity as the co-occurrence of two or more long term conditions. Despite consensus that complex multimorbidity was a useful concept in addition to this, no consensus was reached on how best to define it. Twenty four conditions were rated as ones to "always include," and 37 to "usually include (unless a good reason to exclude in a particular context)." Of the 37 conditions to usually include, untreated and treated hypertension were combined, and conditions that require surveillance has been generally agreed to be included for multimorbidity measurement (criteria for types of conditions to include) and thus treated cancer requiring surveillance was not particularly included in the recommended list of conditions, leading to 35 conditions recommended to usually include in multimorbidity measurement (table 4).

**Table 4:** Conditions reaching consensus to always or usually include in a multimorbidity measure, based on Delphi surveys

Body system (based on ICD-10 chapters)	Always include (n=24)	Usually include (unless a good reason not to in a particular context) (n=35)*
Cardiovascular disease	Stroke, coronary artery disease, heart failure, peripheral artery disease	Heart valve disorders, arrhythmia, venous thromboembolic disease, aneurysm, nypertension (treated and untreated)
Metabolic and endocrine disease	Diabetes, Addison’s disease, cystic fibrosis	Thyroid disorders
Respiratory disease	Chronic obstructive pulmonary disease, asthma	Bronchiectasis
Neurological disease	Parkinson’s disease, epilepsy, multiple sclerosis, paralysis	Transient ischaemic attack, peripheral neuropathy, chronic primary pain
Cancer	Solid organ cancers, haematological cancers, metastatic cancers	Melanoma, benign cerebral tumours that can cause disability
Mental and behavioural disorder	Dementia, schizophrenia	Depression, anxiety, bipolar disorder, drug or alcohol misuse, eating disorder, autism, post-traumatic stress disorder
Musculoskeletal disease	Connective tissue disease	Osteoarthritis, long term musculoskeletal problems due to injury, osteoporosis, gout
Digestive disease	Chronic liver disease, inflammatory bowel disease	Chronic pancreatic disease, peptic ulcer
Urogenital disorder	Chronic kidney disease, end stage kidney disease	Endometriosis, chronic urinary tract infection
Haematological disorder	—	Anaemia (including pernicious anaemia, sickle cell anaemia)
Eye disease	—	Vision impairment that cannot be corrected
Ear disease	—	Hearing impairment that cannot be corrected, Meniere’s disease
Infectious disease	HIV/AIDS	Chronic Lyme disease, tuberculosis, post-acute covid-19
Congenital disease	—	Congenital disease and chromosomal abnormalities

ICD-10=international classification of diseases, 10th revision.

*Untreated and treated hypertension were combined. Conditions that require surveillance (including cancers) were agreed to be included by both sets of panellists, and thus "cancers that require surveillance" was not stated separately in the list.

No conditions were rated by either panel to always exclude or usually exclude, consistent with allowing researchers to choose to additionally include other conditions of particular importance in their context. General criteria reaching consensus in both panels on reasons to select and include conditions in multimorbidity measurement (which could inform such choices) were that a condition was one or more of the following: medical diagnosis; conditions that are currently active; conditions that are permanent in their effects; conditions that require current treatment, care, or therapy; conditions that require surveillance; and remitting-relapsing conditions that require ongoing treatment or care.

Professional and public panels disagreed on how long a condition should persist to be defined as long term, with consensus in the professional panel on ≥6 months versus consensus in the public panel on ≥12 months. Our judgment was to recommend the 12 month cut-off period, but the discrepancy means that other researchers might decide to use a six month cut-off period. Health impacts agreed by both panels as important consideration in the choice of conditions included risk of death, quality of life, frailty, mental health, and treatment burden. As data could be collected from different sources, the consensus was that a consistent approach to multimorbidity measurement should be adopted, irrespective of whether the study used routine data (from patient records or insurance claims databases) or patient self-report. In this study, we found that panellists chose the type of multimorbidity measures depending on study purposes.

Simple counts of conditions were preferred or considered acceptable for estimating prevalence, identifying disease clusters, and exploring trajectories of multimorbidity, whereas weighted measures were preferred or considered acceptable for assessing disease severity and predicting outcomes. No consensus was reached on how to weight measures, consistent with this depending on study purpose, but researchers should therefore explicitly state and justify their choice of how to weight (eg, in relation to severity of disease or in relation to a particular outcome). Stirland et al^26^ provide guidance on which weighted measures to use for a particular purpose for those researchers who judge that a weighted measure is appropriate.^26^

### Strengths and limitations of the study

Strengths of this study include that the surveys were designed on the basis of results of a systematic review and in response to panellists’ input, and that participants were recruited to both professional and public panels with good retention. Limitations include that less than 20% of panellists were from low or middle income countries, meaning that long term conditions prevalent in low or middle income countries might not have been prioritised. The professional panel was also larger than the public panel, meaning that where panels disagreed in which conditions to include, analysis could have favoured the professional perspective. An implication is that the conditions recommended for inclusion are probably best seen as a core list, and that researchers should carefully consider any additional conditions in their context to be included, and ensure public and patient involvement in their choice. However, if reporting prevalence of multimorbidity, then reporting the prevalence using the core list is recommended to improve comparability as well as reporting prevalence using the study specific set of conditions.

Secondly, owing to the difficulty of navigating experts in this relatively new research specialty of multimorbidity, the study results might have differed if those interested in multimorbidity but never involved in multimorbidity research had been included. Finally, the professional and public panels disagreed on a small number of areas, meaning that findings should be interpreted with caution. Future studies could explore these areas of disagreement in more depth than is possible in a Delphi study. More in-depth studies could also explore more technical questions that were not asked of the public panel in this study (eg, relating to the construction of weighted measures).

### Comparison of results with previous studies

Several previous consensus studies and group developed position papers have focused on the definition of multimorbidity, but these typically do not consider how to apply these definitions in measurement.^27 28^ Other studies have highlighted variable measurement of multimorbidity, with large variation in the number and nature of conditions included in measures.^19 29 30^ Prior consensus studies have examined which conditions to include. N’Goran et al^31^ used a modified RAND consensus method with a Swiss family practitioner panel to identify 75 International Classification of Primary Care diagnoses pertinent to the clinical consideration of people with multimorbidity. The main differences with this study were their inclusion of a more heterogeneous set of conditions in the psychological domain (including tobacco abuse and memory disturbance that is not dementia).^31^

Hafezparast et al^32^ aimed to identify local consensus on the choice of conditions to include in a measure relevant to inner city London.^32^ Unspecified participants were asked to rate 86 conditions identified in a scoping review, considering them in terms of their prevalence, impact, preventability and modifiability, treatment burden, disease progression, and data quality. Thirty two conditions were rated as locally important to include in multimorbidity measurement, of which only two were not rated as always or usually include in our study (learning difficulties and morbid obesity). In addition, a qualitative study by Drye et al^33^ identified 10 chronic conditions for quality care measurement (based on their adverse effects on health status, function, and quality of life), all of which were included in the core list of this study. However, several conditions rated as "always or usually include" in our study were not in Drye’s recommendations, such as cancers, schizophrenia, and chronic liver disease.^33^

As previous review has shown that more than half of existing studies did not include mental health conditions in measurement,^19^ the nine mental health conditions rated as "always or usually include" could provide more comprehensive quality measurement for individuals with multimorbidity. Others have noted that the exact choice of conditions is likely to vary by study purpose, that episodic conditions should be included, and that there might be patient characteristics which are very important in clinical care (eg, smoking or socioeconomic status).^29 30^ In line with previous studies, we found consensus on the inclusion of episodic conditions only if they are active, permanent in their effects, or require ongoing treatment or surveillance; but we found no consensus on patient characteristics and social factors in both panels.

### Implications of results

This study has several implications. Firstly, while we recognise that the choice of conditions to include in measurement should be sensitive to purpose and local context,^30^ research in the field would be improved if researchers used a common set of conditions as core, which is provided in the list of conditions to always and usually include be identified in this study (table 4). For studies of prevalence, we recommend that researchers also report age and sex stratified prevalence based on the "always include" and "always or usually include" lists to improve comparability of studies.^10^ More generally, although not the focus of this study, multimorbidity measures are often poorly reported, and clarity about choices made and their rationale is critical (figure 3).^19^ We recommend that selection of other long term conditions in measures should take account of the criteria agreed as important by panellists in this study (figure 2), and that researchers explicitly report why and how they make decisions on condition and measurement choice (figure 3).

Secondly, this study has identified a need for consistent use of validated clinical code lists, but did not seek to identify them. Others have published lists of such codes for use in this context,^34^ and with several initiatives set up to standardise identification of conditions in healthcare data (eg, the Health Data Research UK Phenotype Library^35^).

Thirdly, although others have said that weighted measures are generally preferred over simple counts,^36^ this study provides professional consensus about the particular purposes where simple counts or weighted measures were preferred or considered acceptable (figure 2). However, we need research that considers the relative performance of simple counts and weighted measures (eg, in predicting outcomes), and for wider public discussion about the relevance of weighted measures to patients (eg, in relation to which outcomes measures are weighted against).

Finally, our study found consensus that complex multimorbidity was a useful concept but no clear consensus on how to define it. Researchers who adopt definitions of multimorbidity beyond two or more conditions should therefore clearly justify their choice (figure 3). Research is needed to better understand the experience of complex multimorbidity from a patient perspective, and to examine whether different definitions of complex multimorbidity have better predictive performance than existing measures. We recommend that complex multimorbidity definitions should be co-developed with patients to ensure that these are relevant to their illness experience.

In conclusion, existing measurement of multimorbidity is highly inconsistent. The findings of this Delphi study provide guidance on multimorbidity measurement that will help bring greater consistency to the field, facilitating replication, comparison between studies, and evidence synthesis.

## Data Availability

Data are available upon reasonable request.
