# Supplementary material for: Measuring multimorbidity in research: Delphi consensus study
Source: BMJ Med. 2022 Jul 27;1(1):e000247. doi: 10.1136/bmjmed-2022-000247 (PMC9978673; doi:10.1136/bmjmed-2022-000247)
Supplement: Supplementary data [file bmjmed-2022-000247supp001.pdf]

Measuring multimorbidity in research: a Delphi consensus study

Appendix 1: Study protocol

Ho ISS, *et al. BMJMED* 2022; 1:e000247. doi: 10.1136/bmjmed-2022-000247

Non-CTIMP Study Protocol

A Delphi study to explore an international consensus on the definition and measurement of multimorbidity

	The University of Edinburgh<br>Doorway 3, Medical School, Teviot Place<br>Edinburgh<br>EH8 9AG
Protocol authors	Iris Ho and Bruce Guthrie
Funder	Health Data Research UK (HDRUK)
Funding Reference Number	CFC 0110
Chief Investigator	Professor Bruce Guthrie
Sponsor number	CFC 0110
REC Number	
Project registration	**If applicable trials should be registered on a publically accessible database. ACCORD can provide log-in credentials for clinicaltrials.gov. Please email resgov@accord.scot to arrange**
Version Number and Date	Version 1: 24/02/2020

CONTENTS

1 INTRODUCTION ... 5
1.1 BACKGROUND ... 5
1.2 RATIONALE FOR STUDY ... 6
2 STUDY OBJECTIVES ... 6
2.1 OBJECTIVES ... 6
3 STUDY DESIGN ... 6
3.1 PHASE ONE: A SYSTEMATIC SCOPING REVIEW ... 6
3.1.1 Search strategy ... 7
3.1.2 Study screening ... 7
3.1.3 Data extraction ... 7
3.1.4 Data analysis ... 8
3.2 PHASE TWO: DELPHI STUDY ... 9
3.2.1 Study population ... 9
Inclusion criteria ... 9
3.2.2 Number of participants ... 10
3.2.3 Identifying participants ... 10
3.2.4 Consenting participants ... 10
3.2.5 Withdrawal of Study Participants ... 10
4 DATA COLLECTION ... 10
4.1 DEFINING CONSENSUS OR STABILITY ... 11
4.1.1 Round 1 ... 11
4.1.2 Round 2 ... 11
4.1.3 Round 3 ... 11
4.2 SOURCE DATA DOCUMENTATION ... 11
4.3 CASE REPORT FORMS ... 12
5 DATA MANAGEMENT ... 12
5.1 PERSONAL DATA ... 12
5.2 DATA INFORMATION FLOW ... 12
5.3 TRANSFER OF DATA ... 12
5.4 DATA CONTROLLER ... 12
5.5 DATA BREACHES ... 13
6 STATISTICS AND DATA ANALYSIS ... 13
6.1 SAMPLE SIZE CALCULATION ... 13
6.2 PROPOSED ANALYSES ... 13
7 ADVERSE EVENTS ... 13
8 GOOD CLINICAL PRACTICE ... 14
8.1 ETHICAL CONDUCT ... 14
Before the study commence, all required approvals will be obtained and any conditions of approvals will be met. ..... **Error! Bookmark not defined.**
8.2 INVESTIGATOR RESPONSIBILITIES ... 14
8.2.1 Informed Consent ... 14
8.2.2 Study Site Staff ... 14
8.2.3 Data Recording ... 14

8.2.4 Investigator Documentation 14
8.2.5 GCP Training 14
8.2.6 Confidentiality 15
8.2.7 Data Protection 15

9 STUDY CONDUCT RESPONSIBILITIES 15
9.1 PROTOCOL AMENDMENTS 15
9.2 MANAGEMENT OF PROTOCOL NON COMPLIANCE 15
9.3 SERIOUS BREACH REQUIREMENTS 15
9.4 STUDY RECORD RETENTION 15
9.5 END OF STUDY 16

10 REPORTING, PUBLICATIONS AND NOTIFICATION OF RESULTS 16
10.1 AUTHORSHIP POLICY 16

11 REFERENCES 16

12 APPENDICES 18
APPENDIX 1 – SEARCH STRATEGY 18
APPENDIX 2 – DATA EXTRACTION FORMS FOR THE SCOPING REVIEW 20
APPENDIX 3 – INVITATION EMAIL 21
Survey: Building an international consensus on multimorbidity definition and measures: A Delphi study 21
APPENDIX 4 – FOLLOW-UP EMAIL REMINDER 22
Survey reminder: Building an international consensus on multimorbidity definition and measures: A Delphi study 22
APPENDIX 5 – PARTICIPANT INFORMATION SHEET AND INFORMED CONSENT 23
APPENDIX 6 – SOCIO-DEMOGRAPHIC INFORMATION .. **Error! Bookmark not defined.**

LIST OF ABBREVIATIONS

ACCORD	Academic and Clinical Central Office for Research & Development - Joint office for The University of Edinburgh and Lothian Health Board
CI	Chief Investigator
CRF	Case Report Form
GCP	Good Clinical Practice
ICH	International Conference on Harmonisation
PI	Principal Investigator
QA	Quality Assurance
REC	Research Ethics Committee
SOP	Standard Operating Procedure
CoCoPop	Condition, Context, Population
BOS	Bristol Online Survey

1 INTRODUCTION

1.1 BACKGROUND

In many regions of the world, a growing proportion of the adult population is affected by more than one chronic condition [1-3]. Evidence from several studies indicates that the prevalence of multimorbidity increases substantially with age, and commonly occurs in people aged 65 or older [4-6]. Prevalence is also inversely related to socio-economic status and educational attainment [5, 6]. People living in deprived areas and with lower education tend to be multimorbid at a younger age [1, 4]. Of the population with multimorbidity, approximately 30% to 40% have both a physical and a mental health condition [4, 5]. Women and people with lower education and living in deprived areas are more likely to have physical and mental comorbidity [4, 5].

Multimorbidity is defined as the co-existence of multiple chronic conditions [7].. Unlike comorbidity that focuses on the effects of additional conditions in reference to an index chronic condition, multimorbidity addresses the total effects of chronic conditions without giving priority to any one of the co-existing conditions [8]. In reference to their difference in definition, multimorbidity has, therefore, been placed as a separate Medical Subject Heading (MeSH) since January 2018, distinct from comorbidity [8]. Notwithstanding some agreement on the broad definition of multimorbidity, there remains no international consensus on its

operational definition regarding how to measure it, with the measures varying widely in terms of the number, labelling, type, and severity of included conditions [7].

Without a clear and agreed guide, many measurement tools have been developed and used to measure multimorbidity. The measurement tools used in research and practice include: unweighted disease counts; weighted disease counts; weighted medication counts [9]. In addition, different weighting schemes have been applied to serve different purposes. Consequently, there is a wide discrepancy in prevalence estimates, ranging from 12.9%-95.1% in the general population [3].

Existing research recognises the importance of establishing an agreed approach to the definition and measurement of multimorbidity. Several attempts have been made to synthesise existing evidence on multimorbidity measures [9-11], to compare different measures of multimorbidity to predict certain outcomes [12], and to adapt existing measures to meet the needs of specific regions or populations [13, 14]. Due to the heterogeneity of current approaches, continued efforts are needed to develop an agreed approach as to what constitutes multimorbidity, what should be included in multimorbidity measures, and how to measure it. The aim of this study is to use the Delphi technique to explore international experts' views on the definition and measurement of multimorbidity, and to further provide a comprehensive guide on the use of multimorbidity measures to meet different purposes.

1.2 RATIONALE FOR STUDY

The Delphi technique is an iterative and participatory method to explore experts' opinions, discuss issues and build consensus through a structured group communication process [15]. In the current evidence base, this method has been used in health research for needs assessment [16], policy determination [17], and guideline development [18]. Given the wide range of applications of multimorbidity definition and measures, the Delphi method is considered as a suitable method for this study to collaboratively engage international experts to address the complex issues and support decision-making on how to measure multimorbidity.

2 STUDY OBJECTIVES

2.1 OBJECTIVES

- Objective 1: To identify and summarise published multimorbidity definitions and measures used in the existing literature, and to explore how these vary by the stated purpose of each study through a systematic scoping review
- Objective 2: To develop consensus construction to provide a comprehensive guidance on multimorbidity definition and measurement

3 STUDY DESIGN

This study involves two phases. The first phase is a systematic scoping review to address objective 1, and the second phase is a Delphi study to address objective 2.

3.1 PHASE ONE: A SYSTEMATIC SCOPING REVIEW

We will use a systematic scoping review to examine the broad area of multimorbidity to map key concepts and identify gaps in the evidence [19]. In this review, we will follow the

CoCoPop framework (Condition, Context and Population) to define eligibility criteria and conduct searches. This framework was developed by Munn et al. (2015) for systematic reviews of observational studies [20]. "Condition" is referred to as the variable of interest, which is multimorbidity in this review. In terms of context and population, we will look at studies conducted in hospitals, primary care and community that a population sample of people is being studied. Please see table 1 for the inclusion and exclusion criteria.

Table 1. Inclusion and exclusion criteria

Inclusion criteria:

- Quantitative studies that measured multimorbidity in a defined population
- In any setting, including hospitals, primary care, community
- Studies reported in English

Exclusion criteria:

- Studies that used measures with the presence of an index disease integral to the measures
- Conference proceedings or articles that do not provide full text
- Qualitative research and case series

3.1.1 Search strategy

The search strategy for this review will be developed in collaboration with a medical librarian (Appendix 1). Two sets of key terms (multimorbidity and measure) will be combined to search relevant literature by a means of Boolean logic. Medical subject headings will be used to capture concepts and to see if it yields additional studies in comparison with the results from keyword search. Searches will be conducted in Ovid interface (PsycINFO, Embase, Global Health, Medline), Scopus, Web of Science, Cochrane Library, EBSCO interface (CINAHL Plus), and ProQuest Dissertations & Theses Global, from inception to 21 January, 2020. Following the database searches, reference lists of retrieved articles will be hand-searched and citations will be tracked using Scopus/Google Scholar to ensure the inclusion of all relevant literature.

Conference proceedings and literature that did not provide full text will not be included in the review due to the limited information available for data extraction and analysis.

3.1.2 Study screening

References identified by the search strategy will be exported to EndNote X9 bibliographic software and Excel for deduplication, and then will be imported to Covidence for screening. Titles, abstracts, and full-texts of retrieved articles will be screened against the eligibility criteria by two reviewers (SH and PH). The first reviewer is from a nursing background and trained in systematic review methods and with experience of conducting reviews. The second reviewer is a clinician with experience of providing care for people with multimorbidity. Throughout the review process, any disagreement that arises will be resolved through discussion between the two reviewers or the chief investigator's arbitration.

3.1.3 Data extraction

Pre-designed data extraction tables will be created to organise data (Appendix 2). Where any relevant or important data that do not fit in the tables, the tables will be revised to

facilitate data organisation and capture the holistic picture of the study topic. The types of data that will be extracted include:

- author's name
- year of publication
- study title
- purpose
- methodology (cross sectional/prospective)
- country
- study participants
- number of participants
- reference definition of multimorbidity
- tool used to measure multimorbidity (disease counts, weighted indices, medication-based measures)
- data collection method/data source (self-reports: survey or interview; administrative data, clinical routine data, research data)
- conditions, severity and other elements (e.g. age, gender, sociodemographic information) included in each measure
- rationale for selecting the items
- weighting scheme
- testing of reliability or validity
- prevalence of multimorbidity in the whole population and by sub-groups
- outcome variable
- follow-up years
- results
- confounding factors.

These data will be entered into Microsoft Excel 2016 and will be checked for completeness by SH, PH and BG.

3.1.4 Data analysis

Narrative synthesis will be used to synthesise the evidence. Data will be categorised by collapsing those that are similar and dissimilar into broader and higher order of categories. The relationships and interactions between and within data, categories and observations will be explored (including patterns and processes). Data visualisation tools will be used during data analysis to help make sense of the data. Thereafter, a questionnaire for the Delphi survey will be developed based on review findings and gaps identified from the review.

Ho ISS, et al. BMJMED 2022; 1:e000247. doi: 10.1136/bmjmed-2022-000247

3.2 PHASE TWO: DELPHI STUDY

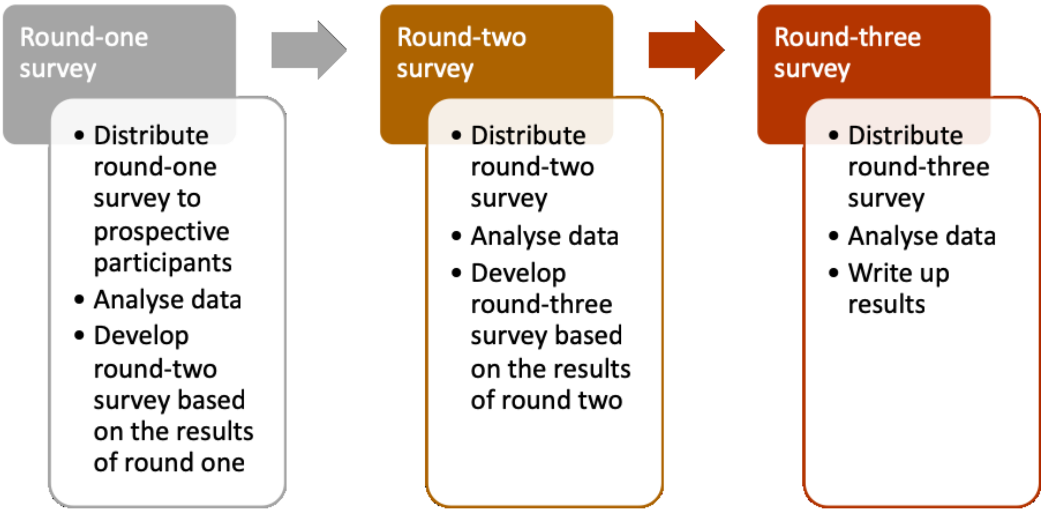

Figure 1: A Map of the project plan

The Delphi technique is designed as a communication process which allows for the inclusion of expert panellists from different sectors to participate in a detailed examination and discussion of a specific problem [15, 21, 22]. Therefore, this method is well suited for consensus building by using a series of questionnaires to collect data from the expert panel. Consensus here is referred to as a statement that is agreed by the majority [15, 23]. As a part of the Delphi process, each individual's responses from each questionnaire will be fed back to the panel alongside a summary of all other responses [15, 24]. The use of successive questionnaires can inform the panel members of the current status of their collective opinions [24]. Typically, more than two rounds of data collection are required to collect needed information and to reach consensus [22, 24, 25].

3.2.1 Study population

Unlike a traditional survey that aims to generalise results, the Delphi is a group decision mechanism requiring individuals who have knowledge, experience or deep understanding of the topic of interest [26]. In that sense, the main focus of this approach is the selection of experts and patients needed for inclusion of all the relevant perspectives [15]. In this proposed Delphi, we aim to recruit international experts and the public who have knowledge and experience of multimorbidity. Individuals who meet one of the following criteria will be included in this study:

Inclusion criteria

- Clinicians with experience of treating patients with more than one chronic condition
- Individuals who have been involved in health policy-making or research where multimorbidity was the focus or relevant
- Members of the public who are interested in multimorbidity or have experience of being multimorbid

3.2.2 Number of participants

There is no direction on the number of participants required for a Delphi survey [15]. The size of expert panel in existing Delphi studies varied considerably from under 10 to 500 [15]. In this Delphi study, the minimum number of experts we plan to recruit is approximately 25-30 experts.

3.2.3 Identifying participants

The expert panellists will be identified by SH (Szu-Szu Ho) and BG (Bruce Guthrie) using publicly available information, including:

1) published work (to identify researchers and policy makers)
2) publicly available websites, reports, and policy documents (to identify healthcare professionals, policy makers or public participants e.g. in guideline development)
3) social media, such as twitter, where we will distribute this study information

or experts will be identified by research participants where they forward the study information to colleagues who meet the criteria.

3.2.4 Consenting participants

Participants will be invited to take part in the study through email along with an attachment of the participant information sheet (PIS) (Appendix 3 and 4). Participants will be asked to read the PIS before deciding to take part, and the survey link will be provided in the PIS to ensure that they have read the study information. Each participant will be given two to three weeks to consider and respond to each round.

3.2.5 Withdrawal of Study Participants

Participants will be informed that they can withdraw their participation at any time by not completing the survey and informing our researcher of their decision. If withdrawal occurs their personally-identifiable information will be permanently deleted, and they will also be allowed to request withdrawal of the data they submitted if they wish.

4 DATA COLLECTION

The Delphi process is iterated until consensus or the consistency of responses between successive rounds of survey is achieved [23]. In this proposed Delphi, we plan to use online questionnaires to collect data. Members of the panel will be anonymous to one another but the research team will know their identity in order that they can be sent individual feedback and invitations to participate in subsequent rounds of survey. The promise of anonymity among panellists is to facilitate participants to be open and truthful about their views on multimorbidity without feeling pressured by more influential panel members [15, 25]. We expect to have three rounds of data collection but will terminate early if consensus is

reached and consider a fourth round if required for elements where consensus is not reached.

4.1 DEFINING CONSENSUS OR STABILITY

Consensus is pre-defined as 70% of participants agreeing/strongly agreeing or disagreeing/strongly disagreeing with a statement. However, not all statements in a Delphi study will gain consensus irrespective of the number of rounds [15, 25]. von der Gracht [23] therefore proposes that the level of consensus should not be the only criterion for stopping the Delphi process, but rather that sufficient clarification of the different viewpoints can also be a desirable goal. We will therefore not only examine consensus, but also stability of responses between rounds. The stability will be measured as the percentage change in agreement with a statement from round to round, and a change lower than 15% will be considered a stable answer [23]. If in round 3, all responses either achieve consensus or stability, then we will terminate the Delphi after round 3. If there are sufficient responses which lack consensus and are unstable, then we will take those items to a round 4.

4.1.1 Round 1

We will use the findings of the scoping review described above to identify the range of multimorbidity measures used in research and practice, their characteristics and use of standards, and purposes for measuring multimorbidity. The existing evidence and gaps identified from the review will serve as a point of reference for developing the round-one Delphi survey. The design of the questionnaire will be structured in a way that includes closed-ended/likert-scaled questions and some open-ended questions. The combination of both types of questions allows each participant an opportunity to generate additional insights, as well as formally scoring items derived by the research team from the literature review. Prospective participants will be required to rate (from strongly agree to strongly disagree or from very important to not important at all) and rank (rank importance of statements on a scale of 1-7) items or statements using Likert scales [15]. The open-ended responses will be triangulated with close-ended responses, and the results will be used to develop new items.

4.1.2 Round 2

In the second round, participants will receive a structured questionnaire and be asked to review their previous response and the items summarised based on the aggregated results of the first round. Second round items will be a mix of those scored in round 1 which did not achieve consensus, and new items based on round 1 open-ended responses. In this round, consensus may begin to form and areas of disagreement and agreement are likely to be identified.

4.1.3 Round 3

Round three will be designed using the results from round two. Prospective participants will receive a questionnaire that includes items or ratings summarised in the previous round and will be asked to review the results and provide their judgements. This round offers an opportunity for panellists to reflect and make further clarifications on the information developed by previous iterations and make their final judgements.
In an attempt to keep the panel motivated, a summary sheet of statements that have achieved consensus will be provided and serve as feedback of completed work to the panel.

4.2 SOURCE DATA DOCUMENTATION

The documents that contain source data are participant-completed questionnaires stored on the Qualtrics or Jisc online survey tools, and in the downloaded csv file. The anonymous

research data will be stored separately from personally-identifiable information, including email address and name (preferred name given by participants). These will be stored securely in the university server with password protection.

4.3 CASE REPORT FORMS

Not applicable

5 DATA MANAGEMENT

5.1 PERSONAL DATA

The personal information that will be collected in this study are: 1) name and email address; 2) country; 3) type of work they are doing; 4) their expertise (Appendix 6). All data will be stored securely on password-protected servers at the University of Edinburgh. The name and email address of participants will be kept separately from the main dataset, and will only be accessible to the research team. Once this study is completed, the file containing participants' names and email addresses will be deleted permanently. Data, other than names and email addresses, will not contain information that can identify participants, and thus will be stored securely and indefinitely on the university server and will not be transferred to external individuals or organisations outside of the sponsoring organisation.

Participants will be assured that their information will be treated confidentially and that their responses would remain anonymous to other panel members throughout the Delphi process. Nonetheless, the researchers will be able to identify participants through their name and email address, which allows for distributing subsequent rounds of survey to the participants and providing them with individual responses and a summary of results from the previous round. They will be informed that they can withdraw their participation at any time. Ethics approval for the study will be sought and obtained from the Usher Research Ethics Committee at the University of Edinburgh.

5.2 DATA INFORMATION FLOW

Data will be collected using Qualtrics or Jisc survey tools and stored in a downloaded csv file for analysis. After the study is completed, participants' personally-identifiable information, name and email address, will be deleted permanently.

5.3 TRANSFER OF DATA

Data collected by the study (including personal data) will not be transferred to any external individuals or organisations outside of the Sponsoring organisation.

5.4 DATA CONTROLLER

The University of Edinburgh and the research team involved in delivering the study will be the data controllers.

5.5 DATA BREACHES

As described above, we will take care to ensure that identifiable information is kept separate from study data. Given the nature of the study data, the potential harm of any data breach is low. We therefore consider the risk of a data breach to be remote and low risk of harm if occurred. All data breaches will be reported to the University of Edinburgh and HDRUK.

6 STATISTICS AND DATA ANALYSIS

6.1 SAMPLE SIZE CALCULATION

There is no one sample size that has been advocated for Delphi studies. It has been suggested that a smaller sample size would be sufficient for a Delphi study, such as 10-15 participants, although others argue that larger sample sizes are likely to produce more generalizable results. Concerned over the lower response rate in online surveys and no guideline for estimating a Delphi sample size, we aim to recruit 25-30 participants for each round.

6.2 PROPOSED ANALYSES

A database will be set up for the analysis of quantitative data (including demographic data) of rounds 1, 2 and 3 using Rstudio (version 4.0.1). Frequencies and descriptive statistics will be used to provide information on the level of agreement with each statement across rounds and expert groups. If the distributions of the responses show less than 15% change from round to round, the responses will be considered to have reached stability [22]. Central tendencies (medians) and levels of dispersion (inter-quartile range) will be provided to see the probability distribution of responses. Bar charts will be created to visually depict the study results. For qualitative data in round one, similar responses will be collapsed and used to modify existing or construct additional unique statements for round 2. New statements created from round 1 will be used for the design of round 2 survey. Response rate for each round of the survey will be calculated.

7 ADVERSE EVENTS

The only plausible potential harm could occur in this study is breach of participants' confidentiality. Precautions will be seriously taken to prevent their identity being disclosed to other panellists and people outside of the research team. Firstly, the file that contains their private information will be kept on the university server with password protection, and separated from the main dataset. Secondly, an invitation email will be sent to participants individually and for the final report, we will use the BCC field to send an email to the participants who would like to receive the report. Once the study is completed, their name and email address will be deleted permanently. Together, these measures are to protect participants' confidentiality.

8 GOOD CLINICAL PRACTICE

8.1 ETHICAL CONDUCT

The study will be conducted in accordance with the principles of the International Conference on Harmonisation Tripartite Guideline for Good Clinical Practice (ICH GCP).

Before the study can commence, all required approvals will be obtained and any conditions of approvals will be met.

8.2 INVESTIGATOR RESPONSIBILITIES

The Investigator is responsible for the overall conduct of the study at the site and compliance with the protocol and any protocol amendments. In accordance with the principles of ICH GCP, the following areas listed in this section are also the responsibility of the Investigator. Responsibilities may be delegated to an appropriate member of study site staff.

8.2.1 Informed Consent

The Investigator is responsible for ensuring informed consent is obtained before any protocol specific procedures are carried out. The decision of a participant to participate in clinical research is voluntary and should be based on a clear understanding of what is involved.

Participants must receive adequate oral and written information – appropriate Participant Information and Informed Consent Forms will be provided. The oral explanation to the participant will be performed by the Investigator or qualified delegated person, and must cover all the elements specified in the Participant Information Sheet and Consent Form.

The participant must be given every opportunity to clarify any points they do not understand and, if necessary, ask for more information. The participant must be given sufficient time to consider the information provided. It should be emphasised that the participant may withdraw their consent to participate at any time without loss of benefits to which they otherwise would be entitled.

The participant will be informed and agree to their medical records being inspected by regulatory authorities and representatives of the sponsor(s).

The Investigator or delegated member of the trial team and the participant will sign and date the Informed Consent Form(s) to confirm that consent has been obtained. The participant will receive a copy of this document and a copy filed in the Investigator Site File (ISF) and participant's medical notes (if applicable).

8.2.2 Study Site Staff

The Investigator must be familiar with the protocol and the study requirements. It is the Investigator's responsibility to ensure that all staff assisting with the study are adequately informed about the protocol and their trial related duties.

8.2.3 Data Recording

Not applicable

8.2.4 Investigator Documentation

Not applicable

8.2.5 GCP Training

For non-CTIMP (i.e. non-drug) studies all researchers are encouraged to undertake GCP training in order to understand the principles of GCP. However, this is not a mandatory requirement unless deemed so by the sponsor. GCP training status for all investigators should be indicated in their respective CVs.

8.2.6 Confidentiality

All laboratory specimens, evaluation forms, reports, and other records must be identified in a manner designed to maintain participant confidentiality. All records must be kept in a secure storage area with limited access. Clinical information will not be released without the written permission of the participant. The Investigator and study site staff involved with this study may not disclose or use for any purpose other than performance of the study, any data, record, or other unpublished information, which is confidential or identifiable, and has been disclosed to those individuals for the purpose of the study.. Prior written agreement from the sponsor or its designee must be obtained for the disclosure of any said confidential information to other parties.

8.2.7 Data Protection

All Investigators and study site staff involved with this study must comply with the requirements of the appropriate data protection legislation (including the General Data Protection Regulation and Data Protection Act) with regard to the collection, storage, processing and disclosure of personal information.

Computers used to collate the data will have limited access measures via user names and passwords.

Published results will not contain any personal data and be of a form where individuals are not identified and re-identification is not likely to take place.

9 STUDY CONDUCT RESPONSIBILITIES

9.1 PROTOCOL AMENDMENTS

Any changes in research activity, except those necessary to remove an apparent, immediate hazard to the participant in the case of an urgent safety measure, must be reviewed and approved by the Chief Investigator.

Amendments will be submitted to a sponsor representative for review and authorisation before being submitted in writing to the appropriate REC, and local R&D for approval prior to participants being enrolled into an amended protocol.

9.2 MANAGEMENT OF PROTOCOL NON COMPLIANCE

Not applicable

9.3 SERIOUS BREACH REQUIREMENTS

A serious breach is a breach which is likely to effect to a significant degree:

(a) the safety or physical or mental integrity of the participants of the trial; or

(b) the scientific value of the trial.

If a potential serious breach is identified by the Chief investigator, Principal Investigator or delegates, the co-sponsors (seriousbreach@accord.scot) must be notified within 24 hours. It is the responsibility of the co-sponsors to assess the impact of the breach on the scientific value of the trial, to determine whether the incident constitutes a serious breach and report to research ethics committees as necessary.

9.4 STUDY RECORD RETENTION

All study documentation will be kept for a minimum of 3 years from the protocol defined end of study point. When the minimum retention period has elapsed, study documentation will not be destroyed without permission from the sponsor.

9.5 END OF STUDY

The end of study is defined as the last participant's last visit.

The Investigators or the co-sponsor(s) have the right at any time to terminate the study for clinical or administrative reasons.

The end of the study will be reported to the REC, and R+D Office(s) and co-sponsors within 90 days, or 15 days if the study is terminated prematurely. The Investigators will inform participants of the premature study closure and ensure that the appropriate follow up is arranged for all participants involved. End of study notification will be reported to the co-sponsors via email to resgov@accord.scot

A summary report of the study will be provided to the REC within 1 year of the end of the study.

10 REPORTING, PUBLICATIONS AND NOTIFICATION OF RESULTS

10.1 AUTHORSHIP POLICY

Ownership of the data arising from this study resides with the study team.

11 REFERENCES

1. Afshar, S., et al., *Multimorbidity and the inequalities of global ageing: a cross-sectional study of 28 countries using the World Health Surveys.* BMC Public Health, 2015. **15**(776): p. 1-10.
2. Garin, N., et al., *Global multimorbidity patterns: a cross-sectional, population-based, multi-country study.* Journals of Gerontology Series A, 2015. **71**(2): p. 205-214.
3. Violan, C., et al., *Prevalence, determinants and patterns of multimorbidity in primary care: a systematic review of observational studies.* PloS one, 2014. **9**(7): p. e1021-1049.
4. Barnett, K., et al., *Epidemiology of multimorbidity and implications for health care, research, and medical education: a cross-sectional study.* The Lancet, 2012. **380**(9836): p. 37-43.
5. Schiøtz, M.L., et al., *Social disparities in the prevalence of multimorbidity – A register-based population study.* BMC Public Health, 2017. **17**(422): p. 1-11.
6. Sakib, M.N., et al., *The prevalence of multimorbidity and associations with lifestyle factors among middle-aged Canadians: an analysis of Canadian Longitudinal Study on Aging data.* BMC Public Health, 2019. **19**(243): p. 1-13.
7. Willadsen, T.G., et al., *The role of diseases, risk factors and symptoms in the definition of multimorbidity – a systematic review.* Scandinavian Journal of Primary Health Care, 2016. **34**(2): p. 112-121.
8. Tugwell, P. and J.A. Knottnerus, *Multimorbidity and Comorbidity are now separate MESH headings.* Journal of Clinical Epidemiology, 2019. **105**: p. vi-viii.
9. Huntley, A.L., et al., *Measures of multimorbidity and morbidity burden for use in primary care and community settings: a systematic review and guide.* The Annals of Family Medicine, 2012. **10**(2): p. 134-141.
10. Diederichs, C., K. Berger, and D.B. Bartels, *The Measurement of Multiple Chronic Diseases—A Systematic Review on Existing Multimorbidity Indices.* The Journals of Gerontology: Series A, 2010. **66A**(3): p. 301-311.
11. de Groot, V., et al., *How to measure comorbidity: a critical review of available methods.* Journal of Clinical Epidemiology, 2003. **56**(3): p. 221-229.

12. Brilleman, S.L. and C. Salisbury, *Comparing measures of multimorbidity to predict outcomes in primary care: a cross sectional study.* Family Practice, 2013. **30**(2): p. 172-178.
13. Calderón-Larrañaga, A., et al., *Assessing and Measuring Chronic Multimorbidity in the Older Population: A Proposal for Its Operationalization.* The Journals of Gerontology, 2016. **72**(10): p. 1417-1423.
14. Wister, A., et al., *Development and validation of a multi-domain multimorbidity resilience index for an older population: results from the baseline Canadian Longitudinal Study on Aging.* BMC Geriatrics, 2018. **18**(170): p. 1-13.
15. Keeney, S., H. McKenna, and F. Hasson, *The Delphi technique in nursing and health research*. 2011, Oxford: John Wiley & Sons.
16. McIlfatrick, S.J. and S. Keeney, *Identifying cancer nursing research priorities using the Delphi technique.* Journal of Advanced Nursing, 2003. **42**(6): p. 629-636.
17. Adler, M. and E. Ziglio, *Gazing into the oracle: The Delphi method and its application to social policy and public health*. 1996, London: Jessica Kingsley Publishers.
18. Schwermer, M., et al., *An expert consensus-based guideline for the integrative anthroposophic treatment of acute gastroenteritis in children.* Complementary Therapies in Medicine, 2019. **45**: p. 289-294.
19. The Joanna Briggs Institute. *The Joanna Briggs Institute Reviewers' Manual 2015 Methodology for JBI Scoping Review*. 2015 [cited 2020 07 January]; Available from: https://nursing.lsuhsc.edu/JBI/docs/ReviewersManuals/Scoping-.pdf.
20. Munn, Z., et al., *Methodological guidance for systematic reviews of observational epidemiological studies reporting prevalence and cumulative incidence data.* International Journal of Evidence-based Healthcare, 2015. **13**: p. 147-153.
21. Hsu, C.-C. and B.A. Sandford, *The Delphi technique: making sense of consensus.* Practical Assessment, Reserach & Evaluation, 2007. **12**(10): p. 1-8.
22. Nair, R., R. Aggarwal, and D. Khanna, *Methods of formal consensus in classification/diagnostic criteria and guideline development.* Semin Arthritis Rheum, 2011. **41**(2): p. 95-105.
23. von der Gracht, H.A., *Consensus measurement in Delphi studies: review and implications for future quality assurance.* Technological Forecasting & Social Change, 2012. **79**: p. 1525-1536.
24. Hasson, F., S. Keeney, and H. McKenna, *Research guidelines for the Delphi survey technique.* Journal of Advanced Nursing, 2000. **32**(4): p. 1008-1015.
25. Milevska-Kostova, N. and W.N. Dunn, *Delphi analysis*, in *Methods and Tools in Public Health: A Handbook for Teachers, Researchers and Health Professionals*, L. Zaletel-Kragelj and J. Bozikov, Editors. 2010, Hans Jacobs Publishing Company: Hellweg. p. 423-436.
26. Okoli, C. and S.D. Pawlowski, *The Delphi method as a research tool: an example, design considerations and applications.* Information & Management, 2004. **42**(1): p. 15-29.

Supplemental material BMJ Publishing Group Limited (BMJ) disclaims all liability and responsibility arising from any reliance placed on this supplemental material which has been supplied by the author(s) BMJMED

12 APPENDICES

APPENDIX 1 – SEARCH STRATEGY

Database	Search strategy
Ovid Interface<br><br>PsycINFO<br>Embase<br>Global Health<br>Ovid MEDLINE	1. (multimorbidit$ or multi-morbidit$ or comorbidit$ or co-morbidit$ or polymorbidit$ or poly-morbidit$ or multicondition$ or multicondition$ or "multiple chronic condition$" or "morbidity burden" or ((multiple or coexisting or co-existing or concurrent or con-current or comorbid or co-morbid) adj2 (disease$ or illness$ or condition$ or diagnos$ or morbid$))).m_titl.<br>2. (measure$ or index or indices or instrument$ or scale$ or "disease count$").mp.<br>3. 1 and 2<br>4. Limit 3 to human
EBSCO Interface<br><br>CINAHL Plus	1. MM (multimorbidit* or multi-morbidit* or comorbidit* or co-morbidit* or polymorbidit* or poly-morbidit* or multicondition* or multicondition* or "multiple chronic condition*" or "morbidity burden" or ((multiple or coexisting or co-existing or concurrent or con-current or comorbid or co-morbid) N2 (disease* or illness* or condition* or diagnos* or morbid*)))<br>2. AB (measure* or index or indices or instrument* or scale*)<br>3. 1 AND 2<br><br>Limiters – Full Text; Human; Language: English
Scopus	TITLE (multimorbidit* or multi-morbidit* or comorbidit* or co-morbidit* or polymorbidit* or poly-morbidit* or multicondition* or multicondition* or "multiple chronic condition*" or "morbidity burden" or ((multiple or coexisting or co-existing or concurrent or con-current or morbid or co-morbid) W/2 (disease* or illness* or condition* or diagnos?s or morbid*))) AND TITLE (measure* or index or indices or instrument* or scale* or "disease counts")
Web of Science	(TI=(measure* or index or indices or instrument* or scale*))AND (TI=(multimorbidit* or multi-morbidit* or comorbidit* or co-morbidit* or polymorbidit* or poly-morbidit* or multicondition* or multicondition* or 'multiple chronic condition*' or 'morbidity burden' or ((multiple or coexisting or co-existing or concurrent or con-current or comorbid or co-morbid) NEAR/2 (disease* or illness* or condition* or diagnos* or morbid*)))) AND LANGUAGE: (English)

Cochrane library	(multimorbidity or multi-morbidity or comorbidity or co-morbidity or polymorbidity or poly-morbidity or multicondition or multicondition or 'multiple chronic conditions' or 'morbidity burden' or ((multiple or coexisting or co-existing or concurrent or con-current or comorbid or co-morbid) NEAR/2 (disease or illness or condition or diagnosis or morbid))) AND (measure or index or indices or instrument or scale or "disease count*"):ti
ProQuest Dissertations & Theses Global	ti((multimorbidit* OR multi-morbidit* OR comorbidit* OR co-morbidit* OR polymorbidit* OR poly-morbidit* OR multicondition* OR multicondition* OR 'multiple chronic condition*' OR 'morbidity burden' OR ((multiple OR coexisting OR co-existing OR concurrent OR con-current OR morbid OR co-morbid) NEAR/2 (disease* OR illness* OR condition* OR diagnos?s OR morbid*)))) AND noft((measure* OR index OR indices OR instrument* OR scale*))<br>Limited by: Manuscript type: Doctoral dissertations,<br>Master's theses<br>Language: English

<Multimorbidity definition and measures>
<Version 1: 24/February/2020>

APPENDIX 2 – DATA EXTRACTION FORMS FOR THE SCOPING REVIEW

Characteristics of multimorbidity measures

Study	Purpose	Participants	Country/Region	Definition of Multimorbidity	Measurement tool	Data collection method	Data source	Elements in the measure	Rationale for selecting the items	Weighting	Reliability /validity	Prevalence of multimorbidity	Outcome variable
								Condition, severity, frailty…					

Confounding factors	Key results

Items of multimorbidity measures

Measure	Condition name	Condition name	Condition name	Condition name	Condition name	Condition name	Condition name	Severity scale	Age
Name of measure		○	○	○	○				
Name of measure	)			○	○	○	○		
Name of measure									

Ho ISS, *et al. BMJMED* 2022; 1:e000247. doi: 10.1136/bmjmed-2022-000247

BMJ Publishing Group Limited (BMJ) disclaims all liability and responsibility arising from any reliance placed on this supplemental material which has been supplied by the author(s)

APPENDIX 3 – INVITATION EMAIL

Subject: Multimorbidity definition and measurement study

Dear [to be addressed individually to each participant],

We are a research team working on a project aiming to explore an international consensus on the definition and measurement of multimorbidity. This study is funded by the Health Data Research UK (HDR UK ─ CFC0110).

We send you this email to invite you to participate in a Delphi survey. Your name and email address were identified from one of your published articles, reports, policy documents, or websites relevant to multimorbidity through our scoping review conducted between January and September, 2020. We seek your participation in this survey as we appreciate your valuable input on multimorbidity definition and measurement. International multimorbidity researchers, clinicians, policy makers and members of the public are all welcome to take part in the study. Please share this survey with your colleagues, if you can, to ensure we reach as many experts as possible. This research experience allows us to share, debate and scrutinise anonymously and move forward through consensus.

The Delphi process consists of **two to four** surveys─ one that needs to be completed by [date] and the other rounds that will be sent out subsequently in the next few months. The first survey will take about 22 minutes to complete. **Taking part in the survey is voluntary and please read the attached Participant Information Sheet (PIS) carefully before you decide to take part. The online survey can be accessed via the link provided in the attached PIS (this is to ensure your acknowledgement of the study information prior to participation).**

The data you provide us will be treated confidentially and will not be accessed by anyone outside of the research team.

The results are envisioned to support and inform research, practice and policy around multimorbidity definition and measurement thanks to your contribution. **A summary of the results will be available to you at the end of the study**.

We hope that you are able to support this important work. If you have any questions, please contact the researcher, Iris Ho or the principal investigator, Bruce Guthrie.

Yours Sincerely,

Professor Bruce Guthrie, Chief investigator, Centre for Population and Health Sciences, Usher Institute, University of Edinburgh, Email: bruce.guthrie@ed.ac.uk

Dr Iris S.S. Ho, Centre for Population and Health Sciences, Usher Institute, University of Edinburgh, Email: iris.s.ho@ed.ac.uk

<Multimorbidity definition and measures>
<Version 1: 24/February/2020>

APPENDIX 4 – FOLLOW-UP EMAIL REMINDER

Subject: Multimorbidity definition and measurement study

Dear [to be addressed individually to each participant],

You were recently invited to participate in a Delphi survey aimed at exploring a consensus on multimorbidity definition and measurement (see the email below). If you have not already completed the survey, we encourage you to take a few munities to do so before [date]. If you have already completed the survey we thank you for your support and ask you to ignore this email. Your valuable opinions can help to shape the future of multimorbidity research and management. If possible, please share this study information with your colleagues in the field.

The survey can be accessed via the link provided in the attached Participant Information Sheet. Please complete the survey by [date].

Thank you in advance.

Yours sincerely,

Professor Bruce Guthrie, Chief investigator, Centre for Population and Health Sciences, Usher Institute, University of Edinburgh, Email: bruce.guthrie@ed.ac.uk

Dr Iris S.S. Ho, Centre for Population and Health Sciences, Usher Institute, University of Edinburgh, Email: iris.s.ho@ed.ac.uk

<Multimorbidity definition and measures>
<Version 1: 24/February/2020>

APPENDIX 5 – PARTICIPANT INFORMATION SHEET

Principal investigator: Professor Bruce Guthrie
Postdoctoral researcher: Dr Iris Ho
Institute: Usher Institute, University of Edinburgh
Research Ethics Committee: Usher Institute Research Ethics Committee at the University of Edinburgh (ref:).
Survey link:

- If you are **clinicians, academics, researchers or policy makers** who have read the PIS and agree to take part, please access the survey via this link:
- If you are **members of the public** who have read the PIS and agree to take part, please access the survey via this link:

You are being invited to take part in this Delphi study aimed at exploring international consensus and guide on the definition and measurement of multimorbidity. Before you decide to take part, it is important that you understand why this research is being conducted and what it involves. Please take time to read this study information carefully.

Thank you for taking the time to read this.

Who will conduct the research?
This research is being conducted by Iris Ho, a researcher at the University of Edinburgh, and Professor Bruce Guthrie (the Chief Investigator). Our names and contact details are given at the end of this information sheet.

Aim of this study
The aim of this study is to explore a consensus on the definition of multimorbidity and on the design of quantitative measures of multimorbidity.

Why have I been invited to take part?
You are invited to participate in this study because we recognised your expertise relevant to multimorbidity through your published work, publicly-available reports, websites, or policy documents following our scoping review. You may also have been invited to take part in this study by a colleague who has forwarded the survey to you.

Do I have to take part?
No – participation is entirely voluntary. You can ask questions about the study before deciding whether or not to participate. If you do agree to participate, please keep this study information sheet and check the “Yes” boxes at the first page of the online survey to indicate that you agree with the listed statements and consent to take part.

Even if you agree to participate, you may withdraw yourself from the study at any time and without giving a reason by advising the researchers of this decision. We will respect your decision to withdraw and there will be no loss of good feeling/will. If withdrawal occurs, your personal and contact information we collected for this project will be permanently deleted. Participant survey responses collected prior to your withdrawal will be kept for analyses, as they will be completely anonymous.

Ho ISS, et al. BMJMED 2022; 1:e000247. doi: 10.1136/bmjmed-2022-000247

<Multimorbidity definition and measures>
<Version 1: 24/February/2020>

What will happen if I decide to take part?
We are inviting you to participate as a panel member in this Delphi study. The Delphi method is used in this study to obtain your opinions and ideas on the definition of multimorbidity, and what should be included in multimorbidity measures and how to use them for different purposes. Two to four rounds of survey will be carried out to ensure that the convergence of opinion on this topic is achieved. This survey takes approximately 22 minutes to complete. A second round of survey will be sent to you via email in a few months following data analysis. Thank you for your contribution.

What are the possible benefits of taking part?
Your valuable input in this Delphi process will allow researchers, practitioners, funders and policy makers in the field to better understand what constitutes multimorbidity, and how to measure it to meet different purposes.

Are there any risks associated with taking part?
There are no foreseeable risks associated with participation in this study.

What happens to the data provided?
We will not tell anyone you have taken part in this study. The research data you give us will be anonymised and stored confidentially at our university server with password protection. Only Bruce Guthrie and Iris Ho have the permission to use the data. The name and email address we collected from you is for sending the subsequent rounds of survey. This information will be permanently deleted after this study is completed. The anonymous research data will be stored securely and indefinitely and will not be transferred to external individuals or organisations outside of the sponsoring organisation.

What will happen with the results of this study?
The results of this study will be summarised in articles and reports, and will be disseminated through peer-reviewed journals and presented at conferences. The results will be made anonymous in any formal outputs, and be available to you all.

Who do I contact if I have a concern about the study or I wish to complain?
If you have any further questions about the study, please contact Dr Iris Ho or the Principal Investigator Professor Bruce Guthrie, who will do their best to answer your query. If you remain unhappy or wish to make a formal complaint, please contact Professor Sarah Cunningham Burley, Dean SMGPHS [Email: sarah.c.burley@ed.ac.uk ; Tel: 0131 650 3217]

Further information and contact details

Principal investigator: Professor Bruce Guthrie [bruce.guthrie@ed.ac.uk]
Postdoctoral researcher: Dr Iris S.S. Ho [iris.s.ho@ed.ac.uk]

Survey link:
- If you are **clinicians, academics, researchers or policy makers** who have read the PIS and agree to take part, please access the survey via this link:
- If you are **members of the public** who have read the PIS and agree to take part, please access the survey via this link:

CFC 0110
24 of 26
