# Supplementary material for: Measuring multimorbidity in research: Delphi consensus study
Source: BMJ Med. 2022 Jul 27;1(1):e000247. doi: 10.1136/bmjmed-2022-000247 (PMC9978673; doi:10.1136/bmjmed-2022-000247)
Supplement: Supplementary data [file bmjmed-2022-000247supp002.pdf]

Measuring multimorbidity in research: a Delphi consensus study

Appendix 2: Surveys

Table of Contents

Round-one survey in the professional panel 2
Round-two survey in the professional panel 19
Round-three survey in the professional panel 37
Round-one survey in the public panel 44
Round-two survey in the public panel 61
Round-three survey in the public panel 80

Round one professional panel

Round-one survey in the professional panel

Socio-demographic information

Q1: Please describe the country where you are currently living or working.

Q2: Please check the category that best describes the type of work you do most of the time (choose one).

- ☐ Research
- ☐ Public policy or other public sector work
- ☐ Clinical Practice
- ☐ Other

Q3: Please check the category that best describes the setting in which you work most of the time (choose one).

- ☐ Government
- ☐ Academia
- ☐ Hospital
- ☐ Primary care/General practice
- ☐ Social care sector
- ☐ Other

Q4: Do you have multiple chronic conditions?

- ☐ Yes
- ☐ No

Q5: Do you have family or friends who have multiple chronic conditions?

- ☐ Yes
- ☐ No

Q6: To understand multimorbidity, what population would you be more interested in (please choose all that apply)?

Round one professional panel

☐ General population
☐ Older people
☐ Middle-aged and older
☐ Socially-deprived population (including homeless people or drug users)
☐ Women
☐ Men
☐ Children
☐ Other

Operational definition of multimorbidity

Q7: Researchers have defined multimorbidity in many ways. Some define it as '2 or more long-term conditions', but others define it as '3 or more', '4 or more' or '5 or more' long-term conditions. How many long-term conditions do you think someone has to have in order to have multimorbidity? Please choose one.

☒ 2 or more long-term conditions
☐ 3 or more long-term conditions
☐ 4 or more long-term conditions
☒ 5 or more long-term conditions
☐ Other, X or more long-term conditions (please only type a number in the box)

Q8: How would you define "condition" for the concept of multimorbidity? (please choose all that apply)

☐ Formal medical diagnoses (e.g. coronary heart disease, alcohol dependence)
☐ Clinical risk factors (e.g. obesity, high cholesterol)
☐ Symptoms that are not formal medical diagnoses (e.g. dizziness or fatigue)
☐ Health behaviours (e.g. smoking or exercise level)
☐ Health impact (e.g. disability or frailty)
☐ Social deprivation and poverty
☐ Consequences of treatment and care (e.g. side effects of medications or the overall burden of treatment)
☐ Other (please specify)

Round one professional panel

Q9: Researchers justify their choice of conditions in many different ways. Please answer the following questions on the principles for selection of chronic conditions in multimorbidity measures.

1) How long-term a condition is: Researchers vary in what they mean by "long-term". How long does a condition have to be to count as long-term? Please choose one.

☐ Conditions lasting for three months or more
☐ Conditions lasting for six months or more
☐ Conditions lasting for twelve months or more
☐ Other, conditions lasting for X months or more (please only type a number for X in the box)

☐

2) Whether a condition is currently active: Please rate the degree to which you agree or disagree with each of the following statements

Statement	Strongly disagree	Disagree	Neither agree nor disagree	Agree	Strongly agree	Don't know
Include conditions which are permanent in their effects (e.g. Parkinson's disease)	☐	☐	☐	☐	☐	☐
Include conditions which are currently active or currently treated (e.g. asthma with intermittent wheeze; asthma using regular inhalers)	☐	☐	☐	☐	☐	☐
Include conditions which may recur but happen rarely (e.g. people with a history of asthma or depression with no current symptoms and not currently on treatment)	☐	☐	☐	☐	☐	☐
Other ☐	☐	☐	☐	☐	☐	☐

3) Whether condition is treated in healthcare: Please rate the degree to which you agree or disagree with each of the following statements

Statement	Strongly disagree	Disagree	Neither agree nor disagree	Agree	Strongly agree	Don't know

Round one professional panel

Only include conditions which usually require formal treatment or care (e.g. hypertension, diabetes, schizophrenia)	☒	☒	☒	☒	☐	☐
Other	☐	☐	☒	☒	☒	☒

4) The impact of the condition on a range of outcomes: Please rate the degree to which you agree or disagree with each of the following statements

Statement	Strongly disagree	Disagree	Neither agree nor disagree	Agree	Strongly agree	Don't know
Include conditions which significantly increase risk of death	☐	☐	☐	☐	☐	☐
Include conditions which significantly reduce health-related quality of life	☐	☐	☐	☐	☐	☐
Include conditions which cause significant physical disability	☐	☐	☐	☐	☐	☐
Include conditions which cause frailty (general physical and/or mental weakness and vulnerability)	☐	☐	☐	☐	☐	☐
Include conditions which significantly worsen mental health	☐	☐	☐	☐	☐	☐
Include conditions which significantly worsen self-perceived health status	☐	☐	☐	☐	☐	☐
Impact: Other	☐	☐	☐	☐	☐	☐

Q10: Researchers vary in how detailed their definitions of 'condition' are. For example, they might be very broad – one category for "lung disease". Or they might be more detailed – separately count "asthma", "chronic obstructive pulmonary disease" and so on. Please choose one option and explain your choice if you like.

Round one professional panel

☐ Broad disease category based on body and mind system (e.g. cardiovascular disease, mental health problems, skin conditions). Please explain:

☐ Individual conditions (e.g. coronary heart disease, hypertension, depression, schizophrenia, eczema, psoriasis). Please explain:

☒ Other. Please explain:

☒ Don't know

Q11: Some conditions included in multimorbidity measures are closely linked to each other. In particular, some conditions can be caused by other conditions. For example, heart attacks can lead to heart failure. Diabetes can lead to kidney failure. How do you think researchers should count conditions in this situation? Please choose one option and explain your choice if you like.

☐ Count all of the conditions that are currently active. Please explain:

☐ Only count the complications (e.g. if people with heart attack develop heart failure, we would count only heart failure). Please explain:

☐ Count the primary health condition (e.g. if people with diabetes develop kidney failure, we would only count diabetes). Please explain:

☐ Other. Please explain:

Round one professional panel

☒ Don't know

What conditions should be included?

In our review of over 500 research studies, we identified all the conditions which researchers have counted when measuring multimorbidity. There is very large variation in which conditions researchers choose. Only seven conditions were counted by more than half of studies. Many conditions were rarely counted. We would like you to rate conditions that you think are more or less important to include in multimorbidity measures. There are now a set of questions organised by body system.

Q12: We are starting with body systems/disease domains. Please rate how important you think it is to include the following systems/domains in a multimorbidity measure.

Condition	Exclude (not important)	Usually exclude (unless a good reason to include in a particular context)	Could include or exclude	Usually include (unless a good reason to exclude in particular context)	Always include (extremely important)	Don't know
Mental health	☐	☐	☐	☐	☐	☐
Cancer	☐	☐	☐	☐	☐	☐
Cardiovascular	☐	☐	☐	☐	☐	☐
Haematological disease	☐	☐	☐	☐	☐	☐
Neurological disease	☐	☐	☐	☐	☐	☐
Metabolic and Endocrine	☐	☐	☐	☐	☐	☐
Musculoskeletal	☐	☐	☐	☐	☐	☐
Digestive	☐	☐	☐	☐	☐	☐
Urogenital	☐	☐	☐	☐	☐	☐
Skin	☐	☐	☐	☐	☐	☐
Ear, Nose and Throat (ENT)	☐	☐	☐	☐	☐	☐
Oral	☐	☐	☐	☐	☐	☐
Ophthalmology	☐	☐	☐	☐	☐	☐
Chronic infections	☐	☐	☐	☐	☐	☐

Q13: Please rate how important you think it is to include the listed mental health conditions in a multimorbidity measure. If you are not sure, just tick 'Don't know'.

Condition	Exclude (not important)	Usually exclude (unless a good reason to include in a particular context)	Could include or exclude	Usually include (unless a good reason to exclude in particular context)	Always include (extremely important)	Don't know
Anxiety	☐	☐	☐	☐	☐	☐
Depression	☐	☐	☐	☐	☐	☐
Dementia	☐	☐	☐	☐	☐	☐

BMJ Publishing Group Limited (BMJ) disclaims all liability and responsibility arising from any reliance placed on this supplemental material which has been supplied by the author(s)

Round one professional panel

Schizophrenia	☐	☐	☐	☐	☐	☐
Substance misuse (including alcohol and drug dependence)	☐	☐	☐	☐	☐	☐
Nicotine dependence	☐	☐	☐	☐	☐	☐
Post-traumatic stress disorder (PTSD)	☐	☐	☐	☐	☐	☐
Bipolar disorder	☐	☐	☐	☐	☐	☐
Chronic insomnia	☐	☐	☐	☐	☐	☐
Dissociative or personality disorders	☐	☐	☐	☐	☐	☐
Eating disorders (including bulimia or anorexia)	☐	☐	☐	☐	☐	☐
Learning disability	☐	☐	☐	☐	☐	☐
Autism	☐	☐	☐	☐	☐	☐
Obsessive compulsive disorder (OCD)	☐	☐	☐	☐	☐	☐
Somatoform disorders (psychological disorder where a person experiences physical symptoms that cannot be explained by medical doctors)	☐	☐	☐	☐	☐	☐
Attention deficit hyperactivity disorder (ADHD)	☐	☐	☐	☐	☐	☐
Other (please specify either one or more conditions): ______________	☐	☐	☐	☐	☐	☐

Q14: Please rate how important you think it is to include the listed cancers in a multimorbidity measure.

Condition	Exclude (not important)	Usually exclude (unless a good reason to include in a particular context)	Could include or exclude	Usually include (unless a good reason to exclude in particular context)	Always include (extremely important)	Don't know
Solid organ cancers (e.g. lung,	☐	☐	☐	☐	☐	☐

Round one professional panel

colon, prostate, breast etc)						
Haematological cancers (e.g. leukaemia, lymphoma, myeloma)	☐	☐	☐	☐	☐	☐
Melanoma (malignant skin cancer)	☐	☐	☐	☐	☐	☐
Non-melanoma skin cancer	☐	☐	☐	☐	☐	☐
Benign cancers (excluding benign skin lumps and bumps)	☐	☐	☐	☐	☐	☐
Metastatic cancers	☐	☐	☐	☐	☐	☐
Other (please specify either one or more conditions): ____________	☐	☐	☐	☐	☐	☐

Q15: For cancers, some researchers count every cancer a person has had. Others only count 'cancer' once even if someone has had more than one type of cancer. Which methods would you recommend. Please choose one:

- ☐ Count all cancers as one
- ☐ Count individual cancers separately.
- ☐ Other

Q16: Please rate how important you think it is to include the listed cardiovascular conditions in a multimorbidity measure.

Condition	Exclude (not important)	Usually exclude (unless a good reason to include in a particular context)	Could include or exclude	Usually include (unless a good reason to exclude in particular context)	Always include (extremely important)	Don't know
Hypertension	☐	☐	☐	☐	☐	☐
Stroke	☐	☐	☐	☐	☐	☐
Transient Ischaemic Attack (mini stroke)	☐	☐	☐	☐	☐	☐
Dyslipidaemia/ Lipid disorder (e.g. high cholesterol)	☐	☐	☐	☐	☐	☐

Round one professional panel

Coronary artery disease (heart attack or angina)	☐	☐	☐	☐	☐	☐
Heart failure	☐	☐	☐	☐	☐	☐
Peripheral artery disease	☐	☐	☐	☐	☐	☐
Arrhythmia	☐	☐	☐	☐	☐	☐
Heart valves problem	☐	☐	☐	☐	☐	☐
Other (please specify either one or more conditions): ____________	☐	☐	☐	☐	☐	☐

Q17: Please rate how important you think it is to include the listed haematological conditions in a multimorbidity measure.

Condition	Exclude (not important)	Usually exclude (unless a good reason to include in a particular context)	Could include or exclude	Usually include (unless a good reason to exclude in particular context)	Always include (extremely important)	Don’t know
Anaemia (including Iron deficiency anaemia, pernicious anaemia, sickle cell anaemia, aplastic anaemia)	☐	☐	☐	☐	☐	☐
Venous thrombotic disease	☐	☐	☐	☐	☐	☐
Other (please specify either one or more conditions): ____________	☐	☐	☐	☐	☐	☐

Q18: Please rate how important you think it is to include the listed neurological conditions in a multimorbidity measure.

Condition	Exclude (not important)	Usually exclude (unless a good reason to include in a particular context)	Could include or exclude	Usually include (unless a good reason to exclude in particular context)	Always include (extremel y important)	Don’t know
Parkinson's disease	☐	☐	☐	☐	☐	☐
Epilepsy (fits/ seizures)	☐	☐	☐	☐	☐	☐
Chronic pain (including chronic low back pain fibromyalgia,	☐	☐	☐	☐	☐	☐

Round one professional panel

trigeminal neuralgia and other chronic pain)						
Migraine or other regular headache	☐	☐	☐	☐	☐	☐
Multiple sclerosis	☐	☐	☐	☐	☐	☐
Peripheral neuropathy	☐	☐	☐	☐	☐	☐
Paralysis/ Hemiplegia/ Paraplegia (not including those caused by stroke)	☐	☐	☐	☐	☐	☐
Other (please specify either one or more conditions): ____________	☐	☐	☐	☐	☐	☐

Q19: Please rate how important you think it is to include the listed metabolic/endocrine/nutritional conditions in a multimorbidity measure.

Condition	Exclude (not important)	Usually exclude (unless a good reason to include in a particular context)	Could include or exclude	Usually include (unless a good reason to exclude in particular context)	Always include (extremely important)	Don't know
Diabetes (any type)	☐	☐	☐	☐	☐	☐
Thyroid problems (including hypothyroidism and hyperthyroidism)	☐	☐	☐	☐	☐	☐
Malnutrition	☐	☐	☐	☐	☐	☐
Other (please specify either one or more conditions): ____________	☐	☐	☐	☐	☐	☐

Q20: Please rate how important you think it is to include the listed musculoskeletal conditions in a multimorbidity measure.

Condition	Exclude (not important)	Usually exclude (unless a good reason to include in a particular context)	Could include or exclude	Usually include (unless a good reason to exclude in particular context)	Always include (extremely important)	Don't know
Osteoporosis (thinning of the bones)	☐	☐	☐	☐	☐	☐
Osteoarthritis	☐	☐	☐	☐	☐	☐
Connective tissue disease	☐	☐	☐	☐	☐	☐
Gout	☐	☐	☐	☐	☐	☐
Long-term musculoskeletal	☐	☐	☐	☐	☐	☐

Round one professional panel

problems due to injury (including hip fracture)						
Other (please specify either one or more conditions): ____________	☐	☐	☐	☐	☐	☐

Q21: Please rate how important you think it is to include the listed respiratory conditions in a multimorbidity measure.

Condition	Exclude (not important)	Usually exclude (unless a good reason to include in a particular context)	Could include or exclude	Usually include (unless a good reason to exclude in particular context)	Always include (extremely important)	Don't' know
Chronic obstructive pulmonary disease	☐	☐	☐	☐	☐	☐
Asthma	☐	☐	☐	☐	☐	☐
Cystic fibrosis	☐	☐	☐	☐	☐	☐
Chronic/Allergic rhinitis	☐	☐	☐	☐	☐	☐
Other (please specify either one or more conditions): ____________	☐	☐	☐	☐	☐	☐

Q22: Please rate how important you think it is to include the listed gastrointestinal conditions in a multimorbidity measure.

Condition	Exclude (not important)	Usually exclude (unless a good reason to include in a particular context)	Could include or exclude	Usually include (unless a good reason to exclude in particular context)	Always include (extremely important)	Don't know
Chronic liver disease (including liver cirrhosis, liver failure and chronic hepatitis)	☐	☐	☐	☐	☐	☐
Inflammatory bowel disease (including *ulcerative colitis and Crohn's disease)*	☐	☐	☐	☐	☐	☐
Irritable bowel syndrome	☐	☐	☐	☐	☐	☐
Diverticular disease	☐	☐	☐	☐	☐	☐
Pancreatic disease	☐	☐	☐	☐	☐	☐

Round one professional panel

Peptic ulcer (including gastric/ stomach ulcers and duodenal ulcers)	☐	☐	☐	☐	☐	☐
Gastroesophageal reflux (acid reflux and heartburn)	☐	☐	☐	☐	☐	☐
Gall bladder problems (including gallstones)	☐	☐	☐	☐	☐	☐
Other (please specify either one or more conditions): ____________	☐	☐	☐	☐	☐	☐

Q23: Please rate how important you think it is to include the listed urogenital conditions in a multimorbidity measure.

Condition	Exclude (not important)	Usually exclude (unless a good reason to include in a particular context)	Could include or exclude	Usually include (unless a good reason to exclude in particular context)	Always include (extremely important)	Don't know
Chronic kidney disease	☐	☐	☐	☐	☐	☐
End-stage kidney disease (including kidney dialysis and transplant)	☐	☐	☐	☐	☐	☐
Kidney or bladder stones	☐	☐	☐	☐	☐	☐
Chronic urinary tract infections (including chronic bladder infections)	☐	☐	☐	☐	☐	☐
Urinary incontinence	☐	☐	☐	☐	☐	☐
Uterus (womb) problems (including prolapse and fibroid)	☐	☐	☐	☐	☐	☐
Polycystic ovary syndrome	☐	☐	☐	☐	☐	☐
Prostatic hypertrophy	☐	☐	☐	☐	☐	☐
Endometriosis	☐	☐	☐	☐	☐	☐
Infertility	☐	☐	☐	☐	☐	☐
Sexual dysfunction	☐	☐	☐	☐	☐	☐
Other (please specify either one or more conditions): ____________	☐	☐	☐	☐	☐	☐

Round one professional panel

Q24: Please rate how important you think it is to include the listed chronic infectious conditions in a multimorbidity measure.

Condition	Exclude (not important)	Expect to usually exclude (unless a good reason to include in a particular context)	Could include or exclude	Expect to usually include (unless a good reason to exclude in particular context)	Always include (extremely important)
HIV/AIDS	☐	☐	☐	☐	☐
Tuberculosis	☐	☐	☐	☐	☐
Other (please specify either one or more conditions): ____________	☐	☐	☐	☐	☐

Q25: Please rate how important you think it is to include the listed skin conditions in a multimorbidity measure.

Condition	Exclude (not important)	Usually exclude (unless a good reason to include in a particular context)	Could include or exclude	Usually include (unless a good reason to exclude in particular context)	Always include (extremely important)	Don't know
Psoriasis	☐	☐	☐	☐	☐	☐
Eczema	☐	☐	☐	☐	☐	☐
Chronic urticarial (chronic hives)	☐	☐	☐	☐	☐	☐
Other (please specify either one or more conditions): ____________	☐	☐	☐	☐	☐	☐

Q26: Please rate how important you think it is to include the listed ENT, eye & oral conditions in a multimorbidity measure.

Condition	Exclude (not important)	Usually exclude (unless a good reason to include in a particular context)	Could include or exclude	Usually include (unless a good reason to exclude in particular context)	Always include (extremely important)	Don't know
Hearing impairment or deafness	☐	☐	☐	☐	☐	☐

Round one professional panel

Meniere's disease	☐	☐	☐	☐	☐	☐
Ear, nose, throat disease (including chronic sinusitis)	☐	☐	☐	☐	☐	☐
Vision impairment or blindness	☐	☐	☐	☐	☐	☐
Cataract	☐	☐	☐	☐	☐	☐
Glaucoma	☐	☐	☐	☐	☐	☐
Edentulism (having no teeth)	☐	☐	☐	☐	☐	☐
Chronic gum disease	☐	☐	☐	☐	☐	☐
Other (please specify either one or more conditions): ____________	☐	☐	☐	☐	☐	☐

Q27: Please rate how important you think it is to include the listed congenital conditions in a multimorbidity measure.

Condition	Exclude (not important)	Usually exclude (unless a good reason to include in a particular context)	Could include or exclude	Usually include (unless a good reason to exclude in particular context)	Always include (extremely important)	Don't know
Congenital disease (conditions that babies are born with, including congenial heart disease, genital anomaly)	☐	☐	☐	☐	☐	☐
Other (please specify either one or more conditions): ____________	☐	☐	☐	☐	☐	☐

Q28: Please rate how important you think it is to include the listed risk factors/health behaviour/symptoms/syndromes in a multimorbidity measure.

Condition	Exclude (not important)	Usually exclude (unless a good reason to include in a particular context)	Could include or exclude	Usually include (unless a good reason to exclude in particular context)	Always include (extremely important)	Don't know
Obesity (body mass index ≥30)	☐	☐	☐	☐	☐	☐
Smoking	☐	☐	☐	☐	☐	☐

Round one professional panel

High blood pressure (untreated)	☐	☐	☐	☐	☐	☐
High cholesterol (untreated)	☐	☐	☐	☐	☐	☐
Sedentary lifestyle	☐	☐	☐	☐	☐	☐
Physical disability	☐	☐	☐	☐	☐	☐
Dizziness (without a specific diagnosis)	☐	☐	☐	☐	☐	☐
Chronic cough (without a specific diagnosis)	☐	☐	☐	☐	☐	☐
Post-sepsis syndrome	☐	☐	☐	☐	☐	☐
Side effects of medications	☐	☐	☐	☐	☐	☐
Treatment burden (the sum of all the hassles of taking medicines or attending appointments)	☐	☐	☐	☐	☐	☐
Social deprivation and poverty	☐	☐	☐	☐	☐	☐
Other (please specify either one or more conditions): ____________	☐	☐	☐	☐	☐	☐

Weighting

Most researchers define multimorbidity by just counting how many conditions someone has (a “simple count” of conditions). Others use a “weighted” count to estimate multimorbidity burden and predict outcomes (e.g. death, hospitalisation, quality of life). For example, a simple count would say that hay fever + heart attack + back pain = 3 conditions. A weighted measure that gave more weight to risk of death might count hay fever = 0.5, high blood pressure = 3, back pain = 0.5, total score = 4 *weighted for risk of death*. However a weighted measure that gave more weight to quality of life might count hay fever = 1, high blood pressure = 0.5, and back pain = 2, total score = 3.5 *weighted for quality of life.*

In practice, weighted counts have been commonly used for predicting outcomes despite the fact that a simple count is the most common type of measures used for various purposes.

Q29: Please describe what type of measures you would use to measure multimorbidity and for what purposes.

☐ Weighted morbidity measures for the purpose of ___ (please describe):

Round one professional panel

☐ A simple count of conditions for the purpose of ___ (please describe):

☐ Other (please describe)

Q30: There is much debate over whether 'weighted morbidity measures' or 'simple counts of conditions' are better at predicting outcomes (e.g. mortality, healthcare utilisation etc..). Please describe what type of measures you would use to understand the impact of multimorbidity on outcomes.

☐ Simple counts of conditions
☐ Weighted morbidity measures
☐ Other (please specify)

Note: A key issue for weighted measures, is which outcomes to focus on. Researchers vary in which outcomes they think are most important. Professionals and patients also vary in which outcomes they think are important.

Q31: Please rate the degree to which the outcomes listed below are important to be weighted against.

Outcome	Not at all important	Slightly important	Important	Fairly important	Very important	No opinion
Mortality	☐	☐	☐	☐	☐	☐
Healthcare use (e.g. number of emergency admissions to hospital; outpatient appointments)	☐	☐	☐	☐	☐	☐
Health-related quality of life	☐	☐	☐	☐	☐	☐
Physical disability	☐	☐	☐	☐	☐	☐
Frailty (general physical and/or	☐	☐	☐	☐	☐	☐

Round one professional panel

mental weakness and vulnerability)						
Mental health	☐	☐	☐	☐	☐	☐
Treatment burden (the sum of all the hassles of taking medicines or attending appointments)	☐	☐	☐	☐	☐	☐
Healthcare costs (how much treatment and care for each individual costs)	☐	☐	☐	☐	☐	☐
Self-perceived health status	☐	☐	☐	☐	☐	☐
Other (please specify)	☐	☐	☐	☐	☐	☐

Q32: What weighting methods would you use in a multimorbidity measure?

☐ Use existing weighted indices.

☐ Empirically derive weights based on the individual impact of diseases on an outcome (e.g. use regression models to calculate weights)

☐ Set rules based on level of severity to grade each condition/disease category (e.g. rate each condition/category based on the rules on if having— presence of a condition: 1 point; treatment: additional 1 point; functional limitation: additional 1 point)

☐ No opinion

☐ Other

Round two professional panel

Round-two survey in the professional panel

Socio-demographic information

Q1: Please describe the country where you are currently living or working.

Q2: Please check the category that best describes the type of work you do most of the time (choose one).

- ☐ Research
- ☐ Public policy or other public sector work
- ☐ Clinical Practice
- ☐ Teaching
- ☐ Other

Q3: Please check the category that best describes the setting in which you work most of the time (choose one).

- ☐ Government
- ☐ Academia
- ☐ Hospital
- ☐ Primary care/General practice
- ☐ Social care sector
- ☐ Other

Q4: Do you have multiple chronic conditions?

- ☐ Yes
- ☐ No

Q5: Do you have family or friends who have multiple chronic conditions?

- ☐ Yes
- ☐ No

Q6: To understand multimorbidity, what population would you be more interested in (please choose all that apply)?

- ☐ General population

Round two professional panel

- ☐ Older people
- ☐ Middle-aged and older
- ☐ Socially-deprived population (including homeless people or drug users)
- ☐ Women
- ☐ Men
- ☐ Children
- ☐ Ethnic minority groups or indigenous populations
- ☐ People with disabilities
- ☐ Other

Operational definition of multimorbidity

Note inserted: In round one, there was no consensus on the number of conditions someone have to have in order to have multimorbidity. There were also numerous free text comments, including some which suggested that there should be distinctions made between 'simple' and 'complex' multimorbidity. In this section, we are therefore asking you the round 1 question about 'number of conditions' and some new questions about 'simple' and 'complex' multimorbidity.

Q7-1: How many long-term conditions do you think someone has to have in order to have multimorbidity? Please choose one.*

- ☐ 2 or more long-term conditions (chosen by 68.5% of panellists in round one)
- ☐ 3 or more long-term conditions (chosen by 28.9% of panellists in round one)
- ☐ 4 or more long-term conditions (chosen by 1.3% of panellists in round one)
- ☐ 5 or more long-term conditions (chosen by 0.7% of panellists in round one)
- ☐ Other, X or more long-term conditions

Q7-2-1: Some studies differentiated "complex multimorbidity" from "basic multimorbidity", and used "complex multimorbidity" to identify people with higher care needs (e.g. older people). Do you agree that defining complex multimorbidity *in addition to a core definition of simple multimorbidity* is useful?

Strongly disagree/Disagree/Neither disagree nor agree/Agree/Strongly agree/Don't know

Q7-2-2: Irrespective of whether you agree, how would you define "complex multimorbidity" based on number of conditions. (please choose one)

- ☐ 3+ conditions irrespective of how many body systems

Round two professional panel

- ☐ 3+ conditions from 3+ body systems
- ☐ 4+ conditions irrespective of how many body systems
- ☐ 4+ conditions from 4+ body systems
- ☐ 5+ conditions irrespective of how many body systems
- ☐ 5+ conditions from 5+ body systems
- ☐ Other ____+ conditions from ____+ body systems

Q7-2-3: Sub-question: In addition to the above option you choose, please describe if there are other statements that, you think, should be included in the definition of "complex multimorbdiity".

- ☐ Any combination of 2+ conditions which includes both physical and mental health conditions
- ☐ Other

Q8:
Note inserted: Based on the results from round one, only medical diagnoses reached ≥70% consensus. Having read a summary of the results from round one, we would like you to answer the question again.

How would you define "condition" for the concept of multimorbidity? (please choose all that apply)

- ☐ Formal medical diagnoses (e.g. coronary heart disease, alcohol dependence)
- ☐ Clinical risk factors (e.g. obesity, high cholesterol)
- ☐ Symptoms that are not formal medical diagnoses (e.g. dizziness or fatigue)
- ☐ Health behaviours (e.g. smoking or exercise level)
- ☐ Health impact (e.g. disability or frailty)
- ☐ Social deprivation and poverty
- ☐ Consequences of treatment and care (e.g. side effects of medications or the overall burden of treatment)
- ☐ Environmental factors (e.g. polluted areas or busy roads)
- ☐ Other (please specify)

Q9-1: Definition of chronicity or long-term

Note inserted: None of the statements in Q9-1 reached ≥ 70% consensus in round one. Most professionals defined long-term conditions as conditions that last for 6 months or more, whereas more than 70% of members of the public considered conditions lasting for 12

Round two professional panel

months or more as “long term”. Having read a summary of responses, please answer Q9-1 again so that we could know if your responses have changed between two rounds.

How long does a condition have to be to count as long-term? Please choose one.*

☐ Conditions lasting for three months or more
☐ Conditions lasting for six months or more
☐ Conditions lasting for twelve months or more
☐ Conditions lasting for eighteen months or more

Q9-2: Recurrence or remission

Note inserted: In round one, more than 70% of panellists strongly agreed that conditions can be included in a multimorbidity measure if they are permanent in their effects, or are currently active or currently treated. There was no consensus for categories related to remitting/relapsing conditions. The first question below was asked in round 1 (reworded in response to feedback), and the second is based on suggestions in feedback. Please rate the degree to which you agree or disagree with the following statements.

Statement	Strongly disagree	Disagree	Neither agree nor disagree	Agree	Strongly agree	Don’t know
Include conditions which may recur	☐	☐	☐	☐	☐	☐
Include remitting-relapsing conditions which have happened during the last five years	☐	☐	☐	☐	☐	☐
Other ☐	☐	☐	☐	☐	☐	☐

Q9-3: Treatment, care or surveillance

Note inserted: Some panellists suggested that some conditions that do not necessarily require treatment or care should be included because of significant impact on the individual. For example, people with arthritis might not be receiving current treatment/care but their quality of life could still be significantly affected. In addition, around 53.7% of panellists would include clinical risk factors many of which do not require treatment. Due to widely divergent views on Q9-3, we would like to ask you to rate the degree to which you agree or disagree with each of the following statements.

Round two professional panel

Statement	Strongly disagree	Disagree	Neither agree nor disagree	Agree	Strongly agree	Don't know
Include conditions which usually require current treatment, care or therapy (e.g. hypertension, diabetes, schizophrenia)	☐	☐	☐	☐	☐	☐
Include conditions which usually require treatment, care or therapy at some point in the future even if not currently treated	☐	☐	☐	☐	☐	☐
Include conditions which usually require surveillance (e.g. treated cancer or depression)	☐	☐	☐	☐	☐	☐
Other	☐	☐	☐	☐	☐	☐

Q9-4: Principles of selecting conditions based on impacts

Note inserted: In the professional version of survey, more than 70% of panellists strongly agreed that quality of life, physical disability and mental health should be taken into account when selecting conditions, and we are not asking you about these again. On the other hand, only risk of death was strongly agreed by more than 70% of members of the public.

Please rate the degree to which you agree or disagree with each of the following statements

Statement	Strongly disagree	Disagree	Neither agree nor disagree	Agree	Strongly agree
Include conditions which significantly increase risk of death	☐	☐	☐	☐	☐
Include conditions which cause frailty (general physical and/or mental weakness and vulnerability)	☐	☐	☐	☐	☐
Include conditions which significantly worsen self-perceived health status	☐	☐	☐	☐	☐
Include conditions which significantly increase treatment burden	☐	☐	☐	☐	☐

Round two professional panel

Include conditions which could be impacted by or impact social deprivation and poverty	☐	☐	☐	☐	☐
Impact: Other [ ]	☐	☐	☐	☐	☐

Q10: Categorisation of conditions/counting methods

Note inserted: In round 1, we asked about how to account for complications of conditions and there was consensus (>80%) that all conditions that are currently active should be counted even if some are complications of other conditions. We are not asking this again. There was no consensus about whether to count broader categories of conditions or individual conditions. There was considerable free-text comment, including about the challenges of identifying specific conditions in electronic medical records. We have therefore revised the options for this question.

Please choose one option that you would prefer to use.

☐ Broad disease category based on body systems (e.g. cardiovascular disease, mental health problems, skin conditions).

☐ Individual conditions (e.g. myocardial infarction, angina, hypertension, depression, schizophrenia, eczema, psoriasis).

☐ Grouping together similar conditions that are in the same body system and treated similarly (e.g. angina and myocardial infarction, or aplastic anaemia and sickle cell anaemia)

☐ Other. Please explain:

[ ]

☐ Don't know

Q11: Data source

Multimorbidity has been measured by existing studies using either self-report or databases (e.g. medical records and administrative databases). Please rate the degree to which you agree or disagree the following statements.

Round two professional panel

Statement	Strongly disagree	Disagree	Neither agree nor disagree	Agree	Strongly agree
Conditions included for multimorbidity measurement should be the same in both self-report and databases (e.g. identifying a core set of conditions or condition framework).	☐	☐	☐	☐	☐
Conditions included in multimorbidity measures should differ between self-report and databases	☐	☐	☐	☐	☐
Other	☐	☐	☐	☐	☐

What conditions should be included?

Q12: System/disease domain

Note inserted: There was consensus that six body systems should always be included in a multimorbidity measure (mental health, cancer, cardiovascular, neurological, metabolic/ endocrine, and musculoskeletal). We are not asking about these again in round 2. Please rate the degree to which the following systems/disease domains, you think, are important.

Condition	Exclude (not important)	Usually exclude (unless a good reason to include in a particular context)	Could include or exclude	Usually include (unless a good reason to exclude in particular context)	Always include (extremely important)	Don't know
Respiratory disease	☐	☐	☐	☐	☐	☐
Haematological disease	☐	☐	☐	☐	☐	☐
Digestive	☐	☐	☐	☐	☐	☐
Urogenital	☐	☐	☐	☐	☐	☐
Skin	☐	☐	☐	☐	☐	☐
Ear, Nose and Throat (ENT)	☐	☐	☐	☐	☐	☐
Oral	☐	☐	☐	☐	☐	☐
Ophthalmology	☐	☐	☐	☐	☐	☐
Chronic infections	☐	☐	☐	☐	☐	☐

Note inserted: The number of conditions included in multimorbidity measurement varies substantially, which has led to the heterogeneity of multimorbidity prevalence. In the following section, we would like to ask you to rate a number of conditions that have been used for multimorbidity measurement, with the aim to identify a set of core conditions and conditions likely included for particular populations or regions.

Q13: Mental health

Note inserted: There was consensus that dementia and schizophrenia should always be included in multimorbidity measurement and we are not asking about these again. Please rate

Round two professional panel

the following mental health conditions again which allows us to see if your responses have changed between rounds. If you are not sure, please tick 'Don't know'.

Condition	Exclude (not important)	Usually exclude (unless a good reason to include in a particular context)	Could include or exclude	Usually include (unless a good reason to exclude in particular context)	Always include (extremely important)	Don't know
Anxiety	☐	☐	☐	☐	☐	☐
Depression	☐	☐	☐	☐	☐	☐
Substance misuse (including alcohol and drug dependence)	☐	☐	☐	☐	☐	☐
Nicotine dependence	☐	☐	☐	☐	☐	☐
Post-traumatic stress disorder (PTSD)	☐	☐	☐	☐	☐	☐
Bipolar disorder	☐	☐	☐	☐	☐	☐
Chronic insomnia	☐	☐	☐	☐	☐	☐
Dissociative or personality disorders	☐	☐	☐	☐	☐	☐
Eating disorders (including bulimia or anorexia)	☐	☐	☐	☐	☐	☐
Learning disability	☐	☐	☐	☐	☐	☐
Autism	☐	☐	☐	☐	☐	☐
Obsessive compulsive disorder (OCD)	☐	☐	☐	☐	☐	☐
Somatoform disorders (psychological disorder where a person experiences physical symptoms that cannot be explained by medical doctors)	☐	☐	☐	☐	☐	☐
Attention deficit hyperactivity disorder (ADHD)	☐	☐	☐	☐	☐	☐
Other (please specify either one or more conditions): ____________	☐	☐	☐	☐	☐	☐

Round two professional panel

Q14: Note inserted: There was consensus that solid organ cancers, haematological cancers and metastatic cancers should always be included in multimorbidity measurement and we are not asking about these again. Please rate the following cancer conditions again which allows us to see if your responses have changed between rounds. If you are not sure, please tick 'Don't know'.

Condition	Exclude (not important)	Usually exclude (unless a good reason to include in a particular context)	Could include or exclude	Usually include (unless a good reason to exclude in particular context)	Always include (extremely important)	Don't know
Melanoma	☐	☐	☐	☐	☐	☐
Non-melanoma skin cancer	☐	☐	☐	☐	☐	☐
Benign cancers (excluding benign skin lumps and bumps)	☐	☐	☐	☐	☐	☐
Other (please specify either one or more conditions): ____________	☐	☐	☐	☐	☐	☐

Q15: Based on the panellists' responses, we have revised the options and would like to ask you to choose the method you would recommend (there were other comments for this question relating to recurrence and remission, but Q9 that you have already answered covers these).

☐ Count all cancers as one
☐ Count individual cancers separately irrespective of which systems they affect (e.g. count gastric cancer and liver cancer separately).
☐ Count individual cancers separately if they affect different systems (e.g. pancreatic cancer and lung cancer).
☐ Other

Q16: Note inserted: There was consensus that stroke, coronary heart disease, heart failure and peripheral artery disease should always be included in multimorbidity measurement and we are not asking about these again. Please rate the following cardiovascular conditions again which allows us to see if your responses have changed between rounds. If you are not sure, please tick 'Don't know'.

Condition	Exclude (not important)	Usually exclude (unless a good reason	Could include or exclude	Usually include (unless a good reason	Always include (extremely important)	Don't know

Round two professional panel

		to include in a particular context)		to exclude in particular context)		
Hypertension (treated)	☐	☐	☐	☐	☐	☐
High blood pressure (untreated)	☐	☐	☐	☐	☐	☐
Transient Ischaemic Attack (mini stroke)	☐	☐	☐	☐	☐	☐
Dyslipidaemia/ Lipid disorder (treated)	☐	☐	☐	☐	☐	☐
High cholesterol (untreated)	☐	☐	☐	☐	☐	☐
Arrhythmia	☐	☐	☐	☐	☐	☐
Heart valve problems (Including Rheumatic heart disease)	☐	☐	☐	☐	☐	☐
Other (please specify either one or more conditions): ____________	☐	☐	☐	☐	☐	☐

Q17: Haematological conditions

Consistent with the results in Q12 that less than 70% of panellists would always include haematological system in multimorbidity measurement, none of the individual haematological conditions was rated by more than 70% of panellists as ‘always include’.

Please rate the following haematological conditions as to whether they should be included or excluded. If you are not sure, please tick ‘Don’t know’.

Condition	Exclude (not important)	Usually exclude (unless a good reason to include in a particular context)	Could include or exclude	Usually include (unless a good reason to exclude in particular context)	Always include (extremely important)	Don’t know
Anaemia (including pernicious anaemia, sickle cell anaemia, aplastic anaemia)	☐	☐	☐	☐	☐	☐
Venous thrombotic disease	☐	☐	☐	☐	☐	☐
Other (please specify either one or more conditions): ____________	☐	☐	☐	☐	☐	☐

Round two professional panel

Q18: Note inserted: There was consensus that Parkinson's disease, epilepsy, multiple sclerosis, and paralysis/ hemiplegia/ paraplegia (not including those caused by stroke) should always be included in multimorbidity measurement and we are not asking about these again. Please rate the following neurological conditions again which allows us to see if your responses have changed between rounds. If you are not sure, please tick ‘Don’t know’.

Condition	Exclude (not important)	Usually exclude (unless a good reason to include in a particular context)	Could include or exclude	Usually include (unless a good reason to exclude in particular context)	Always include (extremel y important)	Don’t know
Migraine or other regular headache	☐	☐	☐	☐	☐	☐
Peripheral neuropathy	☐	☐	☐	☐	☐	☐
Other (please specify either one or more conditions): ____________	☐	☐	☐	☐	☐	☐

Q19: Note inserted: There was consensus that diabetes (any types) should always be included in multimorbidity measurement and we are not asking about these again. Please rate the following metabolic and endocrine conditions again which allows us to see if your responses have changed between rounds. If you are not sure, please tick ‘Don’t know’.

Condition	Exclude (not important)	Usually exclude (unless a good reason to include in a particular context)	Could include or exclude	Usually include (unless a good reason to exclude in particular context)	Always include (extremely important)	Don’t know
Thyroid problems (including hypothyroidism and hyperthyroidism)	☐	☐	☐	☐	☐	☐
Hyper and hypoparathyroidism	☐	☐	☐	☐	☐	☐
Malnutrition (including protein energy deficiency)	☐	☐	☐	☐	☐	☐
Addison’s disease	☐	☐	☐	☐	☐	☐
Other (please specify either one or more conditions): ____________	☐	☐	☐	☐	☐	☐

Q20: Note inserted: There was consensus that connective tissue disease (including rheumatoid arthritis or lupus) should always be included in multimorbidity measurement and we are not asking about these again. Please rate the following musculoskeletal conditions again which allows us to see if your responses have changed between rounds. If you are not sure, please tick ‘Don’t know’.

Round two professional panel

Condition	Exclude (not important)	Usually exclude (unless a good reason to include in a particular context)	Could include or exclude	Usually include (unless a good reason to exclude in particular context)	Always include (extremely important)	Don't know
Osteoporosis	☐	☐	☐	☐	☐	☐
Osteoarthritis	☐	☐	☐	☐	☐	☐
Gout	☐	☐	☐	☐	☐	☐
Long-term musculoskeletal problems due to injury (including low back pain, complex regional pain syndrome, neuropraxia)	☐	☐	☐	☐	☐	☐
Other (please specify either one or more conditions): ____________	☐	☐	☐	☐	☐	☐

Q21: Note inserted: There was consensus that COPD, asthma and cystic fibrosis should always be included in multimorbidity measurement and we are not asking about these again. Please rate the following respiratory conditions again which allows us to see if your responses have changed between rounds.

Condition	Exclude (not important)	Usually exclude (unless a good reason to include in a particular context)	Could include or exclude	Usually include (unless a good reason to exclude in particular context)	Always include (extremely important)	Don't' know
Chronic/Allergic rhinitis (Nose becomes inflamed by allergens, such as dust, pollen, animal fur)	☐	☐	☐	☐	☐	☐
Sleep apnoea	☐	☐	☐	☐	☐	☐
Bronchiectasis	☐	☐	☐	☐	☐	☐
Post-acute COVID-19	☐	☐	☐	☐	☐	☐
Other (please specify either one or more conditions): ____________	☐	☐	☐	☐	☐	☐

Q22: Note inserted: There was consensus that chronic liver disease and inflammatory bowel disease should always be included in multimorbidity measurement and we are not asking about these again. Please rate the following digestive conditions again which allows us to see if your responses have changed between rounds.

Round two professional panel

Condition	Exclude (not important)	Usually exclude (unless a good reason to include in a particular context)	Could include or exclude	Usually include (unless a good reason to exclude in particular context)	Always include (extremely important)	Don't know
Irritable bowel syndrome	☐	☐	☐	☐	☐	☐
Diverticulosis (small pockets in the lining of the intestine)	☐	☐	☐	☐	☐	☐
Pancreatic disease	☐	☐	☐	☐	☐	☐
Peptic ulcer	☐	☐	☐	☐	☐	☐
Gastroesophageal reflux	☐	☐	☐	☐	☐	☐
Gall bladder problems (including gallstones)	☐	☐	☐	☐	☐	☐
Other (please specify either one or more conditions): ____________	☐	☐	☐	☐	☐	☐

Q23: Note inserted: There was consensus that chronic kidney disease and end-stage kidney disease should always be included in multimorbidity measurement and we are not asking about these again. Please rate the following urogenital conditions again which allows us to see if your responses have changed between rounds.

Condition	Exclude (not important)	Usually exclude (unless a good reason to include in a particular context)	Could include or exclude	Usually include (unless a good reason to exclude in particular context)	Always include (extremely important)	Don't know
Kidney or bladder stones	☐	☐	☐	☐	☐	☐
Chronic urinary tract infections (including chronic bladder infections)	☐	☐	☐	☐	☐	☐
Urinary incontinence (loss of control over passing urine)	☐	☐	☐	☐	☐	☐
Uterus (womb) problems (including prolapse and fibroid)	☐	☐	☐	☐	☐	☐
Polycystic ovary syndrome	☐	☐	☐	☐	☐	☐

Round two professional panel

Prostatic hypertrophy (large prostate glands)	☐	☐	☐	☐	☐	☐
Endometriosis	☐	☐	☐	☐	☐	☐
Infertility	☐	☐	☐	☐	☐	☐
Sexual dysfunction	☐	☐	☐	☐	☐	☐
Other (please specify either one or more conditions): ____________	☐	☐	☐	☐	☐	☐

Q24: Note inserted: There was consensus that HIV/AIDS should always be included in multimorbidity measurement and we are not asking about these again. Please rate the following chronic infection conditions again which allows us to see if your responses have changed between rounds.

Condition	Exclude (not important)	Expect to usually exclude (unless a good reason to include in a particular context)	Could include or exclude	Expect to usually include (unless a good reason to exclude in particular context)	Always include (extremely important)
Tuberculosis	☐	☐	☐	☐	☐
Lyme disease	☐	☐	☐	☐	☐
Other (please specify either one or more conditions): ____________	☐	☐	☐	☐	☐

Q25: Note inserted: None of the individual skin conditions was rated by more than 70% of panellists as 'always include'. Please rate the following skin conditions as to whether they should be included or excluded. If you are not sure, please tick 'Don't know'.

Condition	Exclude (not important)	Usually exclude (unless a good reason to include in a particular context)	Could include or exclude	Usually include (unless a good reason to exclude in particular context)	Always include (extremely important)	Don't know
Psoriasis	☐	☐	☐	☐	☐	☐
Eczema	☐	☐	☐	☐	☐	☐
Chronic urticarial (chronic hives)	☐	☐	☐	☐	☐	☐
Other (please specify either one or more conditions): ____________	☐	☐	☐	☐	☐	☐

Round two professional panel

Q26: Note inserted: None of the individual ENT, eye and oral conditions was rated by more than 70% of panellists as ‘always include’.Please rate the following ENT, eye and oral conditions as to whether they should be included or excluded. If you are not sure, please tick ‘Don’t know’.

Condition	Exclude (not important)	Usually exclude (unless a good reason to include in a particular context)	Could include or exclude	Usually include (unless a good reason to exclude in particular context)	Always include (extremely important)	Don’t know
Hearing impairment or Deafness (that cannot be easily corrected with hearing aids)	☐	☐	☐	☐	☐	☐
Meniere’s disease (an ear condition that causes sudden attacks of vertigo)	☐	☐	☐	☐	☐	☐
Ear, nose, throat disease (including chronic sinusitis)	☐	☐	☐	☐	☐	☐
Vision impairment or Blindness (that cannot be easily corrected with glasses)	☐	☐	☐	☐	☐	☐
Cataract	☐	☐	☐	☐	☐	☐
Glaucoma	☐	☐	☐	☐	☐	☐
Edentulism (having no teeth)	☐	☐	☐	☐	☐	☐
Chronic gum disease	☐	☐	☐	☐	☐	☐
Other (please specify either one or more conditions): ____________	☐	☐	☐	☐	☐	☐

Q27: Note inserted: Congenital condition was rated by less than 70% of panellists as ‘always include’. Please rate the following congenital conditions as to whether they should be included or excluded. If you are not sure, please tick ‘Don’t know’.

Condition	Exclude (not important)	Usually exclude (unless a good reason to include in a particular context)	Could include or exclude	Usually include (unless a good reason to exclude in particular context)	Always include (extremely important)	Don’t know
Congenital disease (conditions that	☐	☐	☐	☐	☐	☐

Round two professional panel

babies are born with, including congenial heart disease, chromosomal aberrations)						
Other (please specify either one or more conditions): ____________	☐	☐	☐	☐	☐	☐

Q28: Note inserted: The following risk factors/symptoms have been included in multimorbidity measures by existing studies, but none of them was rated by more than 70% of panellists as 'always include'. Some suggested that those should be measured separately rather than as part of a multimorbdiity measure.

Having read a summary of the results from round one, please rate the following statement as to whether this type of conditions should be included or excluded again. If you are not sure, please tick 'Don't know'.

Condition	Exclude (not important)	Usually exclude (unless a good reason to include in a particular context)	Could include or exclude	Usually include (unless a good reason to exclude in particular context)	Always include (extremely important)	Don't know
Obesity (body mass index ≥30)	☐	☐	☐	☐	☐	☐
Smoking	☐	☐	☐	☐	☐	☐
Sedentary lifestyle	☐	☐	☐	☐	☐	☐
Physical disability	☐	☐	☐	☐	☐	☐
Dizziness or vertigo (without a specific diagnosis)	☐	☐	☐	☐	☐	☐
Chronic cough (without a specific diagnosis)	☐	☐	☐	☐	☐	☐
Post-sepsis syndrome	☐	☐	☐	☐	☐	☐
Side effects of medications	☐	☐	☐	☐	☐	☐
Treatment burden (the sum of all the hassles of taking medicines or attending appointments)	☐	☐	☐	☐	☐	☐
Social deprivation and poverty	☐	☐	☐	☐	☐	☐
Other (please specify either one or more conditions): ____________	☐	☐	☐	☐	☐	☐

Round two professional panel

Weighting

Q29: Weighted count versus simple count

For the following purposes, please choose which type of multimorbidity measure you would prefer to use.

Statement	Simple counts	Weighted measures	Both types of measures	Don't know
For the purpose of estimating the prevalence of multimorbidity, I prefer to use_____	☐	☐	☐	☐
For the purpose of identifying and counting disease clusters, I prefer to use_____	☐	☐	☐	☐
For the purpose of exploring/identifying predictors of multimorbidity (e.g. socio-demographic information), , I prefer to use_____	☐	☐	☐	☐
For the purpose of exploring trajectories of multimorbidity (e.g. trends of multimorbidity prevalence and the number of conditions an individual has had), I prefer to use_____	☐	☐	☐	☐
For the purpose of assessing the severity of disease burden, I prefer to use_____	☐	☐	☐	☐
For the purpose of risk adjustment or outcome prediction, I prefer to use_____	☐	☐	☐	☐

Q31: Outcomes

Note inserted: In round one, you were invited to rate which outcomes are important to be weighted against. Mortality and quality of life were considered very important in round one. However, adapting a measure that fits all purposes (predicting all outcomes) may not be feasible. It has also been suggested that healthcare use and costs should be separated from patient-centred outcomes, and distinct measures for these two outcomes are necessary. Another discrepancy identified from round one is that some preferred to use simple counts for predicting outcomes, but others preferred to use weighted measures. Instead of rating levels of importance, we would like to ask you to choose which type of multimorbidity measures you would prefer to use for predicting the following outcomes.

Outcome	Simple counts	Weighted measures	Both types of measures	No opinion/don't know

Round two professional panel

Mortality	☐	☐	☐	☐
Healthcare use (e.g. number of emergency admissions to hospital; outpatient appointments)	☐	☐	☐	☐
Health-related quality of life	☐	☐	☐	☐
Physical disability	☐	☐	☐	☐
Frailty (general physical and/or mental weakness and vulnerability)	☐	☐	☐	☐
Treatment burden (the sum of all the hassles of taking medicines or attending appointments)	☐	☐	☐	☐
Healthcare costs (how much treatment and care for each individual costs)	☐	☐	☐	☐
Self-perceived health status	☐	☐	☐	☐
Other (please specify)	☐	☐	☐	☐

Q32: Having reflected results from round one and your responses to the questions so far, please choose which weighting method you would prefer to use in a multimorbidity measure (which allows to see if the responses have changed over time).

☐ Use existing weighted indices.

☐ Empirically derive weights based on the individual impact of diseases on an outcome (e.g. use regression models to calculate weights)

☐ Apply weights by defining specific criteria to each condition/disease category (e.g. set rules based on level of severity to grade each condition/disease category)

☐ No opinion

☐ Other

Round three professional panel

Round-three survey in the professional panel

Socio-demographic information

Q1: Please describe the country where you are currently living or working.

Q2: Please check the category that best describes the type of work you do most of the time (choose one).

- ☐ Research
- ☐ Public policy or other public sector work
- ☐ Clinical Practice
- ☐ Teaching
- ☐ Other

Q3: Please check the category that best describes the setting in which you work most of the time (choose one).

- ☐ Government
- ☐ Academia
- ☐ Hospital
- ☐ Primary care/General practice
- ☐ Social care sector
- ☐ Other

Q4: Do you have multiple chronic conditions?

- ☐ Yes
- ☐ No

Q5: Do you have family or friends who have multiple chronic conditions?

- ☐ Yes
- ☐ No

Round three professional panel

Q6: To understand multimorbidity, what population would you be more interested in (please choose all that apply)?

- ☐ General population
- ☐ Older people
- ☐ Middle-aged and older
- ☐ Socially-deprived population (including homeless people or drug users)
- ☐ Women
- ☐ Men
- ☐ Children
- ☐ Ethnic minority groups or indigenous populations
- ☐ People with disabilities
- ☐ Other

What is multimorbidity?

Note: In both the professional panel and public panel, there was consensus (≥ 70%) that someone had to have two or more chronic conditions in order to have multimorbidity. In addition, more than 70% of panellists also strongly agreed that conditions included in multimorbidity measurement should have the following characteristics: 1) permanent in their effects, 2) currently active, 3) lasting six months or longer, 4) requiring current treatment, care or therapy. However, less than 70% of professional panellists agreed that it was useful to conceptualise complex multimorbidity in addition to basic multimorbidity. It is therefore not entirely clear whether and how we should define complex multimorbidity. Please answer the following questions again.

Q7: Please rate the degree to which you agree or disagree with the following statement again so we can see if there is a change between round 2 and round 3.

	Strongly disagree	Disagree	Neither disagree nor agree	Agree	Strongly agree	Don't know
Do you agree that defining complex multimorbidity in addition to a core definition of simple multimorbidity is useful?						

Q8: Irrespective of whether you agree that complex morbidity is a useful concept, we would still like to invite you to define "complex multimorbidity" based on number of conditions. (please choose one)

- ☐ 3+ conditions irrespective of how many body systems

Round three professional panel

☐ 3+ conditions from 2+ body systems
☐ 3+ conditions from 3+ body systems
☐ 4+ conditions irrespective of how many body systems
☐ No opinion
☐ Other

Note: In the professional and public panel, there was no consensus on what additional patterns of conditions should be included in the definition of complex multimorbidity. We have added a few more statements based on panellists' comments.

Q8-1: Sub-question: Please choose the following statements that, you think, should be included in the definition of "complex multimorbidity".

☐ No other patterns that I would like to include
☐ Any combination of two or more conditions which includes both physical and mental health conditions
☐ Any combination of two or more conditions with significantly physical functional limitation
☐ Difficulty in managing illnesses due to social factors/social determinants of health (e.g. poverty).
☐ Any combination of two or more conditions and frailty
☐ Other

Q9. Recurrence or remission

Note inserted: More than 70% of panellists in the professional and public panels agreed that conditions that may recur or remit could be included in the measurement of multimorbidity. However, it remains uncertain as to how this type of conditions should be defined in the concept of multimorbidity. Some suggested suggested take 'treatment' into account when deciding which remitting-relapsing conditions to include, and thus we have revised the statements.

Please rate the degree to which you agree or disagree with the following statements.

Statement	Strongly disagree	Disagree	Neither disagree nor agree	Agree	Strongly agree	Don't know
Include remitting-relapsing conditions that require ongoing treatment/therapy/care (e.g. depression, epilepsy)—newly added	☐	☐	☐	☐	☐	☐
Include remitting-relapsing conditions which have relapsed during the last five years	☐	☐	☐	☐	☐	☐

Round three professional panel

Q10: Treatment, care or surveillance

Note inserted: More than 70% of panellists strongly agreed to include conditions that require current treatment, care, or therapy, and this question will not be asked again in round three. Please rate the degree to which you agree or disagree with the following statements.

Statement	Strongly disagree	Disagree	Neither disagree nor agree	Agree	Strongly agree	Don't know
Include conditions which usually require treatment, care or therapy 'at some point in the future' even if not currently treated (one panellist suggested to conceptualise this as 'current risk to future health outcomes')	☐	☐	☐	☐	☐	☐
Include conditions which usually require surveillance (one panellist suggested to conceptualise this as 'current health needs or complexity of providing care')	☐	☐	☐	☐	☐	☐

Q11: Categorisation of conditions/counting methods

Please choose one option that you would prefer to use.

☐ Broad disease category based on body and mind system (e.g. cardiovascular disease, mental health problems, skin conditions).

☐ Individual conditions (e.g. TIA (mini stroke) and stroke are counted separately).

☐ Grouping together similar conditions that are in the same category and treated similarly (e.g. group together 'Angina and Myocardial Infarction' or 'Aplastic anaemia and Sickle cell anaemia')

☐ Other. Please explain:

☒ Don't know

Q12: Data source

Please rate the following statement again.

Round three professional panel

Statement	Strongly disagree	Disagree	Neither disagree nor agree	Agree	Strongly agree	Don't know
Conditions included for multimorbidity measurement should be the same/similar in both self-report and databases (e.g. identifying a core set of conditions or condition framework).	☐	☐	☐	☐	☐	☐
Other comments	☐	☐	☐	☐	☐	☐

What conditions should be included?

Q13: Note inserted: In cancer conditions, we added two new statements based on panellists' comments. Please rate the following cancer conditions as to whether they should be included or excluded. If you are not sure, please tick 'Don't know'.

Condition	Exclude (not important)	Usually exclude (unless a good reason to include in a particular context)	Could include or exclude	Usually include (unless a good reason to exclude in particular context)	Always include (extremely important)	Don't know
Treated cancer that requires surveillance	☐	☐	☐	☐	☐	☐
Treated cancer that did not recur over the past 5 years and does not require surveillance	☐	☐	☐	☐	☐	☐
Benign cerebral tumours (brain tumours that can cause functional limitations)	☐	☐	☐	☐	☐	☐

Q14: Please choose the categorisation method you would prefer again.

☐ Count all primary cancers as one
☐ Count individual primary cancers separately irrespective of which systems they affect (e.g. count gastric cancer and liver cancer separately).
☐ Count individual primary cancers separately only if they affect different systems (e.g. pancreatic cancer and lung cancer).
☐ Other

Round three professional panel

Q15: Note inserted: Conditions relevant to chronic pain and consequences of injury were revised based on panellists' comments. Please rate the following conditions as to whether they should be included or excluded. If you are not sure, please tick 'Don't know'.

Condition	Exclude (not important)	Usually exclude (unless a good reason to include in a particular context)	Could include or exclude	Usually include (unless a good reason to exclude in particular context)	Always include (extremel y important)	Don't know
Chronic or recurrent low back pain	☐	☐	☐	☐	☐	☐
Chronic primary pain (defined as pain with no clear underlying condition but signfiicant impact on the person)	☐	☐	☐	☐	☐	☐
Long-term musculoskeletal problems due to injury (e.g. consequences of accidental injuries)	☐	☐	☐	☐	☐	☐

Q16: Conditions newly added in round two

Please rate again the following conditions so we can see if your responses have changed between round two and round three

Condition	Exclude (not important)	Usually exclude (unless a good reason to include in a particular context)	Could include or exclude	Usually include (unless a good reason to exclude in particular context)	Always include (extremel y important)	Don't know
Addison's disease	☐	☐	☐	☐	☐	☐
Bronchiectasis	☐	☐	☐	☐	☐	☐
Post-acute covid 19 ("long COVID")	☐	☐	☐	☐	☐	☐
Chronic Lyme disease	☐	☐	☐	☐	☐	☐
Aneurysm	☐	☐	☐	☐	☐	☐

Type of measures to use for a particular purpose

Q17: Which type of measures you would use for a particular purpose? (Please tick one or both - if you choose both a simple count and weighted measures, we would count either as being acceptable/usable for that purpose)

Round three professional panel

Type of measures	Estimating the prevalence of multimorbidity	Identifying and counting disease clusters	Exploring trajectories of multimorbidity	Exploring/identifying predictors of multimorbidity	Assessing the severity of disease burden	Risk adjustment or outcome prediction
Prefer simple count of conditions	☐	☐	☐	☐	☐	☐
Prefer weighted measure	☐	☐	☐	☐	☐	☐
Either is acceptable/ useable	☐	☐	☐	☐	☐	☐
Don't know	☐	☒	☐	☐	☐	☐

Q18: Which type of measures you would use for prediction of a particular outcome? (Please tick one or both - if you choose both a simple count and weighted measures, we would count it as either being acceptable/usable for that purpose)

Type of measures	Risk of death	Health-related quality of life	Physical disability	Frailty	Treatment burden	Healthcare use	Healthcare costs	Self-perceived health	Mental health
Prefer simple count of conditions	☐	☐	☐	☐	☐	☐	☐	☐	☐
Prefer weighted measure	☐	☐	☐	☐	☐	☐	☐	☐	☐
Either is acceptable / useable	☐	☐	☐	☐	☐	☐	☐	☐	☐
Don't know	☐	☐	☐	☐	☐	☐	☐	☐	☐

Round one public panel

Round-one survey in the public panel

Socio-demographic information

Q1: Please describe the country where you are currently living.

Q2: What is your age?

- ☐ 18-34
- ☐ 35-54
- ☐ 55-64
- ☐ ≥ 65
- ☐ Prefer not to answer

Q3: What gender do you identify as?

- ☐ Female
- ☐ Male
- ☐ Other __________
- ☐ Prefer not to answer

Q4: We are seeking your views as a member of the public, but we know that some of you will have worked in healthcare or academia. Do you currently or did you previously work in academia, health and social care practice or healthcare policy?

- ☐ Yes
- ☐ No

Q5: Do you have multiple chronic conditions?

- ☐ Yes
- ☐ No

Q6: Do you have family or friends who have multiple chronic conditions?

- ☐ Yes
- ☐ No

What is multimorbidity?

Q7: Researchers have defined multimorbidity in many ways. Some define it as ‘2 or more long-term conditions’, but others define it as ‘3 or more’, ‘4 or more’ or ‘5 or more’ long-term conditions. How many long-term conditions do you think someone has to have in order to have multimorbidity? Please choose one.

Round one public panel

- ☐ 2 or more long-term conditions
- ☐ 3 or more long-term conditions
- ☐ 4 or more long-term conditions
- ☐ 5 or more long-term conditions
- ☐ Other, X or more long-term conditions (please only type a number in the box)

Q8: How would you define "condition" for the concept of multimorbidity? (please choose all that apply)

- ☐ Formal medical diagnoses (e.g. coronary heart disease, alcohol dependence)
- ☐ Clinical risk factors (e.g. obesity, high cholesterol)
- ☐ Symptoms that are not formal medical diagnoses (e.g. dizziness or fatigue)
- ☐ Health behaviours (e.g. smoking or exercise level)
- ☐ Health impact (e.g. disability or frailty)
- ☐ Social deprivation and poverty
- ☐ Consequences of treatment and care (e.g. side effects of medications or the overall burden of treatment)
- ☐ Other (please specify)

Q9: Researchers justify their choice of conditions in many different ways. Please answer the following questions on the principles for selection of chronic conditions in multimorbidity measures.

1) How long-term a condition is:
   Researchers vary in what they mean by "long-term". How long does a condition have to be to count as long-term? Please choose one.

   - ☐ Conditions lasting for three months or more
   - ☐ Conditions lasting for six months or more
   - ☐ Conditions lasting for twelve months or more
   - ☐ Other, conditions lasting for X months or more (please only type a number for X in the box)

2) Whether a condition is currently active: Please rate the degree to which you agree or disagree with each of the following statements

Round one public panel

Statement	Strongly disagree	Disagree	Neither disagree nor agree	Agree	Strongly agree	Don't know
Include conditions which are permanent in their effects (e.g. Parkinson's disease)	☐	☐	☐	☐	☐	☐
Include conditions which are currently active or currently treated (e.g. asthma with intermittent wheeze; asthma using regular inhalers)	☐	☐	☐	☐	☐	☐
Include conditions which may recur but happen rarely (e.g. people with a history of asthma or depression with no current symptoms and not currently on treatment)	☒	☐	☐	☐	☐	☐
Other	☐	☐	☐	☐	☐	☐

3) Whether condition is treated in healthcare: Please rate the degree to which you agree or disagree with each of the following statements

Statement	Strongly disagree	Disagree	Neither disagree nor agree	Agree	Strongly agree	Don't know
Only include conditions which usually require formal treatment or care (e.g. hypertension, diabetes, schizophrenia)	☐	☐	☐	☐	☐	☐
Other	☐	☐	☐	☐	☐	☐

4) The impact of the condition on a range of outcomes: Please rate the degree to which you agree or disagree with each of the following statements

Statement	Strongly agree	Agree	Neither agree nor disagree	Disagree	Strongly disagree	Don't know

Round one public panel

Include conditions which significantly increase risk of death	☐	☐	☐	☐	☐	☐
Include conditions which significantly reduce quality of life	☐	☐	☐	☐	☐	☐
Include conditions which cause significant physical disability	☐	☐	☐	☐	☐	☐
Include conditions which cause frailty (general physical and/or mental weakness and vulnerability)	☐	☐	☐	☐	☐	☐
Include conditions which significantly worsen mental health	☐	☐	☐	☐	☐	☐
Include conditions which significantly worsen how people perceive their general health	☐	☐	☐	☐	☐	☐
Other impact	☐	☐	☐	☐	☐	☐

Q10: Researchers vary in how detailed their definitions of 'condition' are. For example, they might be very broad – one category for "lung disease". Or they might be more detailed – separately count "asthma", "chronic obstructive pulmonary disease" and so on. Please choose one option and explain your choice if you like.

☐ Broad disease category based on body and mind system (e.g. cardiovascular disease, mental health problems, skin conditions). Please explain:

☐ Individual conditions (e.g. coronary heart disease, hypertension, depression, schizophrenia, eczema, psoriasis). Please explain:

☒ Other. Please explain:

Round one public panel

☐ Don't know

Q11: Some conditions included in multimorbidity measures are closely linked to each other. In particular, some conditions can be caused by other conditions. For example, heart attacks can lead to heart failure. Diabetes can lead to kidney failure. How do you think researchers should count conditions in this situation? Please choose one option and explain your choice if you like.

☐ Count all of the conditions that are currently active. Please explain:

☐ Only count the complications (e.g. if people with heart attack develop heart failure, we would count only heart failure). Please explain:

☐ Count the primary health condition (e.g. if people with diabetes develop kidney failure, we would only count diabetes). Please explain:

☐ Other. Please explain:

☒ Don't know

What conditions should be included?

In our review of over 500 research studies, we identified all the conditions which researchers have counted when measuring multimorbidity. There is very large variation in which conditions researchers choose. Only seven conditions were counted by more than half of studies. Many conditions were rarely counted. We would like you to rate conditions that you think are more or less important to include in multimorbidity measures. There are now a set of questions organised by body system.

Q12: We are starting with body systems/disease domains. Please rate how important you think it is to include the following systems/domains in a multimorbidity measure.

Round one public panel

Condition	Exclude (not important)	Usually exclude (unless a good reason to include in a particular context)	Could include or exclude	Usually include (unless a good reason to exclude in particular context)	Always include (extremely important)	Don't know
Mental health	☐	☐	☐	☐	☐	☐
Cancer	☐	☐	☐	☐	☐	☐
Cardiovascular	☐	☐	☐	☐	☐	☐
Blood disease (which affects formation of blood cells and blood clots)	☐	☐	☐	☐	☐	☐
Neurological disease (which affects the nervous system)	☐	☐	☐	☐	☐	☐
Metabolic and Endocrine (which affects metabolic regulation)	☐	☐	☐	☐	☐	☐
Musculoskeletal (which affects joints, bones and muscles)	☐	☐	☐	☐	☐	☐
Digestive (which affects stomach, bowels, liver, pancreas and gallbladder)	☐	☐	☐	☐	☐	☐
Urogenital (which affects urinary and genital organs)	☐	☐	☐	☐	☐	☐
Skin	☐	☐	☐	☐	☐	☐
Ear, Nose and Throat (ENT)	☐	☐	☐	☐	☐	☐
Oral	☐	☐	☐	☐	☐	☐
Ophthalmology (which affects eyes)	☐	☐	☐	☐	☐	☐
Chronic infections	☐	☐	☐	☐	☐	☐

Q13: Please rate how important you think it is to include the listed mental health conditions in a multimorbidity measure. If you are not sure, just tick 'Don't know'.

Condition	Exclude (not important)	Usually exclude (unless a good reason to include in a particular context)	Could include or exclude	Usually include (unless a good reason to exclude in particular context)	Always include (extremely important)	Don't know
Anxiety	☐	☐	☐	☐	☐	☐
Depression	☐	☐	☐	☐	☐	☐
Dementia	☐	☐	☐	☐	☐	☐
Schizophrenia	☐	☐	☐	☐	☐	☐

Round one public panel

Substance misuse (including alcohol and drug dependence)	☐	☐	☐	☐	☐	☐
Nicotine dependence	☐	☐	☐	☐	☐	☐
Post-traumatic stress disorder (PTSD)	☐	☐	☐	☐	☐	☐
Bipolar disorder	☐	☐	☐	☐	☐	☐
Chronic insomnia	☐	☐	☐	☐	☐	☐
Dissociative or personality disorders (altering to be a person with different personality and the alters are imaginary)	☐	☐	☐	☐	☐	☐
Eating disorders (including bulimia or anorexia)	☐	☐	☐	☐	☐	☐
Learning disability	☐	☐	☐	☐	☐	☐
Autism	☐	☐	☐	☐	☐	☐
Obsessive compulsive disorder (OCD)	☐	☐	☐	☐	☐	☐
Somatoform disorders (psychological disorder where a person experiences physical symptoms that cannot be explained by medical doctors)	☐	☐	☐	☐	☐	☐
Attention deficit hyperactivity disorder (ADHD)	☐	☐	☐	☐	☐	☐
Other (please specify either one or more conditions): ____________	☐	☐	☐	☐	☐	☐

Q14: Please rate how important you think it is to include the listed cancers in a multimorbidity measure.

Condition	Exclude (not important)	Usually exclude (unless a	Could include or exclude	Usually include (unless a	Always include	Don’t know

Round one public panel

		good reason to include in a particular context)		good reason to exclude in particular context)	(extremely important)	
Solid organ cancers (e.g. lung, colon, prostate, breast etc)	☐	☐	☐	☐	☐	☐
Haematological cancers (cancer that affects blood, e.g. leukaemia, lymphoma, myeloma)	☐	☐	☐	☐	☐	☐
Melanoma (malignant skin cancer)	☐	☐	☐	☐	☐	☐
Non-melanoma skin cancer	☐	☐	☐	☐	☐	☐
Benign cancers (excluding benign skin lumps and bumps)	☐	☐	☐	☐	☐	☐
Metastatic cancers (which have spread to other parts of the body)	☐	☐	☐	☐	☐	☐
Other (please specify either one or more conditions): ____________	☐	☐	☐	☐	☐	☐

Q15: For cancers, some researchers count every cancer a person has had. Others only count 'cancer' once even if someone has had more than one type of cancer. Which methods would you recommend. Please choose one:

- ☐ Count all cancers as one
- ☐ Count individual cancers separately.
- ☐ Other

Q16: Please rate how important you think it is to include the listed cardiovascular conditions in a multimorbidity measure.

Condition	Exclude (not important)	Usually exclude (unless a good reason to include in a particular context)	Could include or exclude	Usually include (unless a good reason to exclude in particular context)	Always include (extremely important)	Don't know

Round one public panel

Hypertension	☐	☐	☐	☐	☐	☐
Stroke	☐	☐	☐	☐	☐	☐
Transient Ischaemic Attack (mini stroke)	☐	☐	☐	☐	☐	☐
Lipid disorder (e.g. high cholesterol)	☐	☐	☐	☐	☐	☐
Coronary artery disease (heart attack or angina)	☐	☐	☐	☐	☐	☐
Heart failure	☐	☐	☐	☐	☐	☐
Peripheral artery disease (circulation problems in the legs)	☐	☐	☐	☐	☐	☐
Heart rhythm problem (irregular or very fast heart beats)	☐	☐	☐	☐	☐	☐
Heart valves problem (leaking or tight heart valves)	☐	☐	☐	☐	☐	☐
Aneurysm (a weakening or bulging of an artery wall)	☐	☐	☐	☐	☐	☐
Other (please specify either one or more conditions): ____________	☐	☐	☐	☐	☐	☐

Q17: Please rate how important you think it is to include the listed haematological conditions in a multimorbidity measure.

Condition	Exclude (not important)	Usually exclude (unless a good reason to include in a particular context)	Could include or exclude	Usually include (unless a good reason to exclude in particular context)	Always include (extremely important)	Don't know
Anaemia	☐	☐	☐	☐	☐	☐
Venous thrombotic disease (blood clots formed inside a blood vessel that can block circulation)	☐	☐	☐	☐	☐	☐
Other (please specify either one or more conditions): ____________	☐	☐	☐	☐	☐	☐

Round one public panel

Q18: Please rate how important you think it is to include the listed neurological conditions in a multimorbidity measure.

Condition	Exclude (not important)	Usually exclude (unless a good reason to include in a particular context)	Could include or exclude	Usually include (unless a good reason to exclude in particular context)	Always include (extremely important)	Don't know
Parkinson's disease	☐	☐	☐	☐	☐	☐
Epilepsy (fits/ seizures)	☐	☐	☐	☐	☐	☐
Chronic pain (including chronic low back pain and other neurological pain)	☐	☐	☐	☐	☐	☐
Migraine or other regular headache	☐	☐	☐	☐	☐	☐
Multiple sclerosis	☐	☐	☐	☐	☐	☐
Peripheral neuropathy (damage to the nerves going to legs or arms)	☐	☐	☐	☐	☐	☐
Paralysis/ Hemiplegia/ Paraplegia (not including those caused by stroke)	☐	☐	☐	☐	☐	☐
Other (please specify either one or more conditions): ____________	☐	☐	☐	☐	☐	☐

Q19: Please rate how important you think it is to include the listed metabolic/endocrine/nutritional conditions in a multimorbidity measure.

Condition	Exclude (not important)	Usually exclude (unless a good reason to include in a particular context)	Could include or exclude	Usually include (unless a good reason to exclude in particular context)	Always include (extremely important)	Don't know
Diabetes (any type)	☐	☐	☐	☐	☐	☐
Thyroid problems	☐	☐	☐	☐	☐	☐
Malnutrition	☐	☐	☐	☐	☐	☐
Other (please specify either one or more conditions): ____________	☐	☐	☐	☐	☐	☐

Q20: Please rate how important you think it is to include the listed musculoskeletal conditions in a multimorbidity measure.

Round one public panel

Condition	Exclude (not important)	Usually exclude (unless a good reason to include in a particular context)	Could include or exclude	Usually include (unless a good reason to exclude in particular context)	Always include (extremely important)	Don't know
Osteoporosis (thinning of the bones)	☐	☐	☐	☐	☐	☐
Osteoarthritis (wearing and tearing of joints that leads to inflammation and degeneration	☐	☐	☐	☐	☐	☐
Connective tissue disease (autoimmune disease that can affect our bones, cartilages or joints, such as rheumatoid arthritis or lupus)	☐	☐	☐	☐	☐	☐
Gout (crystals that formed inside or around joints, causing pain)	☐	☐	☐	☐	☐	☐
Long-term musculoskeletal problems due to injury (including hip fracture)	☐	☐	☐	☐	☐	☐
Other (please specify either one or more conditions): ____________	☐	☐	☐	☐	☐	☐

Q21: Please rate how important you think it is to include the listed respiratory conditions in a multimorbidity measure.

Condition	Exclude (not important)	Usually exclude (unless a good reason to include in a particular context)	Could include or exclude	Usually include (unless a good reason to exclude in particular context)	Always include (extremely important)	Don't' know
Chronic obstructive pulmonary disease (COPD – smoking related lung damage)	☐	☐	☐	☐	☐	☐
Asthma	☐	☐	☐	☐	☐	☐
Sleep apnoea (breathing	☐	☐	☐	☐	☐	☐

Round one public panel

problems when asleep)						
Cystic fibrosis (inherited condition that damages lungs)	☐	☐	☐	☐	☐	☐
Chronic/Allergic rhinitis (long-term nasal inflammation)	☐	☐	☐	☐	☐	☐
Other (please specify either one or more conditions): ____________	☐	☐	☐	☐	☐	☐

Q22: Please rate how important you think it is to include the listed digestive conditions in a multimorbidity measure.

Condition	Exclude (not important)	Usually exclude (unless a good reason to include in a particular context)	Could include or exclude	Usually include (unless a good reason to exclude in particular context)	Always include (extremely important)	Don't know
Chronic liver disease (including liver cirrhosis, liver failure and chronic hepatitis)	☐	☐	☐	☐	☐	☐
Inflammatory bowel disease (including ulcerative colitis and Crohn's disease)	☐	☐	☐	☐	☐	☐
Irritable bowel syndrome	☐	☐	☐	☐	☐	☐
Diverticular disease (small pockets in the lining of the intestine)	☐	☐	☐	☐	☐	☐
Pancreatic disease	☐	☐	☐	☐	☐	☐
Peptic ulcer (including gastric/ stomach ulcers and duodenal ulcers)	☐	☐	☐	☐	☐	☐
Gastroesophageal reflux (acid reflux and heartburn)	☐	☐	☐	☐	☐	☐
Gall bladder problems (including gallstones)	☐	☐	☐	☐	☐	☐
Other (please specify either one or more conditions): ____________	☐	☐	☐	☐	☐	☐

Round one public panel

Q23: Please rate how important you think it is to include the listed urogenital conditions in a multimorbidity measure.

Condition	Exclude (not important)	Usually exclude (unless a good reason to include in a particular context)	Could include or exclude	Usually include (unless a good reason to exclude in particular context)	Always include (extremely important)	Don't know
Chronic kidney disease	☐	☐	☐	☐	☐	☐
End-stage kidney disease (including kidney dialysis and transplant)	☐	☐	☐	☐	☐	☐
Kidney or bladder stones	☐	☐	☐	☐	☐	☐
Chronic urinary tract infections (including chronic bladder infections)	☐	☐	☐	☐	☐	☐
Urinary incontinence (loss of control over passing urine)	☐	☐	☐	☐	☐	☐
Uterus (womb) problems (including prolapse and fibroid)	☐	☐	☐	☐	☐	☐
Polycystic ovary syndrome	☐	☐	☐	☐	☐	☐
Prostatic hypertrophy (large prostate glands)	☐	☐	☐	☐	☐	☐
Endometriosis	☐	☐	☐	☐	☐	☐
Infertility	☐	☐	☐	☐	☐	☐
Sexual dysfunction	☐	☐	☐	☐	☐	☐
Other (please specify either one or more conditions): ____________	☐	☐	☐	☐	☐	☐

Q24: Please rate how important you think it is to include the listed chronic infectious conditions in a multimorbidity measure.

Condition	Exclude (not important)	Expect to usually exclude (unless a good reason to include in a particular context)	Could include or exclude	Expect to usually include (unless a good reason to exclude in particular context)	Always include (extremely important)

Round one public panel

HIV/AIDS	☐	☐	☐	☐	☐
Tuberculosis	☐	☐	☐	☐	☐
Other (please specify either one or more conditions): _____________	☐	☐	☐	☐	☐

Q25: Please rate how important you think it is to include the listed skin conditions in a multimorbidity measure.

Condition	Exclude (not important)	Usually exclude (unless a good reason to include in a particular context)	Could include or exclude	Usually include (unless a good reason to exclude in particular context)	Always include (extremely important)	Don't know
Psoriasis	☐	☐	☐	☐	☐	☐
Eczema	☐	☐	☐	☐	☐	☐
Chronic urticarial (chronic hives)	☐	☐	☐	☐	☐	☐
Other (please specify either one or more conditions): _____________	☐	☐	☐	☐	☐	☐

Q26: Please rate how important you think it is to include the listed ENT, eye & oral conditions in a multimorbidity measure.

Condition	Exclude (not important)	Usually exclude (unless a good reason to include in a particular context)	Could include or exclude	Usually include (unless a good reason to exclude in particular context)	Always include (extremely important)	Don't know
Hearing impairment or deafness	☐	☐	☐	☐	☐	☐
Meniere's disease (an ear condition that causes sudden attacks of vertigo)	☐	☐	☐	☐	☐	☐
Ear, nose, throat disease (including chronic sinusitis)	☐	☐	☐	☐	☐	☐
Vision impairment or blindness	☐	☐	☐	☐	☐	☐
Cataract	☐	☐	☐	☐	☐	☐
Glaucoma	☐	☐	☐	☐	☐	☐
Edentulism (having no teeth)	☐	☐	☐	☐	☐	☐

Round one public panel

Chronic gum disease	☐	☐	☐	☐	☐	☐
Other (please specify either one or more conditions): ____________	☐	☐	☐	☐	☐	☐

Q27: Please rate how important you think it is to include the listed congenital conditions in a multimorbidity measure.

Condition	Exclude (not important)	Usually exclude (unless a good reason to include in a particular context)	Could include or exclude	Usually include (unless a good reason to exclude in particular context)	Always include (extremely important)	Don't know
Congenital disease (conditions that babies are born with, including congenial heart disease, genital anomaly)	☐	☐	☐	☐	☐	☐
Other (please specify either one or more conditions): ____________	☐	☐	☐	☐	☐	☐

Q28: Please rate how important you think it is to include the listed risk factors/health behaviour/symptoms/syndromes in a multimorbidity measure.

Condition	Exclude (not important)	Usually exclude (unless a good reason to include in a particular context)	Could include or exclude	Usually include (unless a good reason to exclude in particular context)	Always include (extremely important)	Don't know
Obesity (body mass index ≥30)	☐	☐	☐	☐	☐	☐
Smoking	☐	☐	☐	☐	☐	☐
High blood pressure (untreated)	☐	☐	☐	☐	☐	☐
High cholesterol (untreated)	☐	☐	☐	☐	☐	☐
Sedentary lifestyle (spending most of the day sitting)	☐	☐	☐	☐	☐	☐
Physical disability	☐	☐	☐	☐	☐	☐
Dizziness (without a specific diagnosis)	☐	☐	☐	☐	☐	☐

Round one public panel

Chronic cough (without a specific diagnosis)	☐	☐	☐	☐	☐	☐
Post-sepsis syndrome	☐	☐	☐	☐	☐	☐
Side effects of medications	☐	☐	☐	☐	☐	☐
Treatment burden (the sum of all the hassles of taking medicines or attending appointments)	☐	☐	☐	☐	☐	☐
Social deprivation and poverty	☐	☐	☐	☐	☐	☐
Other (please specify either one or more conditions): ____________	☐	☐	☐	☐	☐	☐

Weighting

Q29: Most researchers define multimorbidity by just counting how many conditions someone has (a "simple count" of conditions). Others use a "weighted" count to estimate multimorbidity burden and predict outcomes (e.g. death, hospitalisation, quality of life). For example, a simple count would say that hay fever + heart attack + back pain = 3 conditions. A weighted measure that gave more weight to risk of death might count hay fever = 0.5, high blood pressure = 3, back pain = 0.5, total score = 4 *weighted for risk of death*. However a weighted measure that gave more weight to quality of life might count hay fever = 1, high blood pressure = 0.5, and back pain = 2, total score = 3.5 *weighted for quality of life.* In theory, weighted scores are better than simple counts at predicting if someone is going to have the outcome focused on.

A key issue for weighted measures, is which outcomes to focus on. Researchers vary in which outcomes they think are most important. Professionals and patients also vary in which outcomes they think are important.

We would like to know which outcomes you think are most important.

Outcome	Not at all important	Slightly important	Important	Sufficiently important	Very important	No opinion
Death	☐	☐	☐	☐	☐	☐
Healthcare use (e.g. number of emergency admissions to hospital; outpatient appointments)	☐	☐	☐	☐	☐	☐
Quality of life	☐	☐	☐	☐	☐	☐
Physical disability	☐	☐	☐	☐	☐	☐
Frailty (general physical and/or mental weakness and vulnerability)	☐	☐	☐	☐	☐	☐

Round one public panel

Mental health	☐	☐	☐	☐	☐	☐
Treatment burden (the sum of all the hassles of taking medicines or attending appointments)	☐	☐	☐	☐	☐	☐
Healthcare costs (how much treatment and care for each individual costs)	☐	☐	☐	☐	☐	☐
How people perceive their general health overall (e.g. whether it is excellent, very good, fair, poor, very poor)	☐	☐	☐	☐	☐	☐
Other (please specify) ________________	☐	☐	☐	☐	☐	☐
Other (please specify) _________________	☐	☐	☐	☐	☐	☐

Round two public panel

Round-two survey in the public panel

Socio-demographic information

Q1: Please describe the country where you are currently living.

Q2: What is your age?

- ☐ 18-34
- ☐ 35-54
- ☐ 55-64
- ☐ ≥ 65
- ☐ Prefer not to answer

Q3: What gender do you identify as?

- ☐ Female
- ☐ Male
- ☐ Other __________
- ☐ Prefer not to answer

Q4: We are seeking your views as a member of the public, but we know that some of you will have worked in healthcare or academia. Do you currently or did you previously work in academia, health and social care practice or healthcare policy?

- ☐ Yes
- ☐ No

Q5: Do you have multiple chronic conditions?

- ☐ Yes
- ☐ No

Q6: Do you have family or friends who have multiple chronic conditions?

- ☐ Yes
- ☐ No

What is multimorbidity?

Note inserted: In round one, more than 80% of panellists defined multimorbidity as the co-occurrence of two or more long-term conditions. This question has been agreed by more than 70% of panellists and thus will not be asked again in round two.

Round two public panel

There were also numerous free text comments, including some which suggested that there should be distinctions made between 'simple' and 'complex' multimorbidity. In this section, we are therefore asking you some new questions about 'simple' and 'complex' multimorbidity.

Q7-1: Do you agree that defining complex multimorbidity *in addition to a core definition of simple multimorbidity* is useful?

Strongly disagree/Disagree/Neither disagree nor agree/Agree/Strongly agree/Don't know

Q7-2: Irrespective of whether you agree, how would you define "complex multimorbidity" based on number of conditions. (please choose one)

☐ 3+ conditions irrespective of how many body systems
☐ 3+ conditions from 3+ body systems
☐ 4+ conditions irrespective of how many body systems
☐ 4+ conditions from 4+ body systems
☐ 5+ conditions irrespective of how many body systems
☐ 5+ conditions from 5+ body systems
☐ Other ____+ conditions from ____+ body systems

Q7-3: Sub-question: In addition to the above option you choose, please describe if there are other statements that, you think, should be included in the definition of "complex multimorbdiity".

☐ Any combination of 2+ conditions which includes both physical and mental health conditions
☐ Other

Q8: Defining 'condition' for multimorbidity measurement

Note inserted: Based on the results from round one, only medical diagnoses reached ≥70% consensus. Having read a summary of the results from round one, we would like you to answer the question again.

How would you define "condition" for the concept of multimorbidity? (please choose all that apply)

☐ Formal medical diagnoses (e.g. coronary heart disease, alcohol dependence)
☐ Clinical risk factors (e.g. obesity, high cholesterol)
☐ Symptoms that are not formal medical diagnoses (e.g. dizziness or fatigue)
☐ Health behaviours (e.g. smoking or exercise level)

Round two public panel

☐ Health impact (e.g. disability or frailty)
☐ Social deprivation and poverty
☐ Consequences of treatment and care (e.g. side effects of medications or the overall burden of treatment)
☐ Other (please specify)

Q9: Recurrence or remission

Note-inserted: In round one, more than 70% of members of the public defined long-term conditions as conditions that last for 6 months or more, and strongly agreed that conditions can be included in a multimorbidity measure if they are permanent in their effects. There was no consensus for categories related to active conditions and remitting/relapsing conditions. The first two questions below were asked in round 1 (reworded in response to feedback), and the third is based on suggestions in feedback. Please rate the degree to which you agree or disagree with the following statements.

Statement	Strongly disagree	Disagree	Neither agree nor disagree	Agree	Strongly agree	Don't know
Include conditions which are currently active	☐	☐	☐	☐	☐	☐
Include conditions which may recur	☐	☐	☐	☐	☐	☐
Include remitting-relapsing conditions which have happened during the last five years	☐	☐	☐	☐	☐	☐
Other	☐	☐	☐	☐	☐	☐

Q10: Treatment, care or surveillance

Note inserted: Some panellists suggested that some conditions that do not necessarily require treatment or care should be included because of significant impact on the individual. For example, people with arthritis might not be receiving current treatment/care but their quality of life could still be significantly affected. In addition, around 60% of panellists would

Round two public panel

include clinical risk factors many of which do not require treatment. Due to widely divergent views on Q10, we would like to ask you to rate the degree to which you agree or disagree with each of the following statements.

Statement	Strongly disagree	Disagree	Neither agree nor disagree	Agree	Strongly agree	Don't know
Include conditions which usually require current treatment, care or therapy (e.g. hypertension, diabetes, schizophrenia), diabetes, schizophrenia)	☐	☐	☐	☐	☐	☐
Include conditions which usually require treatment, care or therapy at some point in the future even if not currently treated	☐	☐	☐	☐	☐	☐
Include conditions which usually require surveillance (e.g. treated cancer or depression)	☐	☐	☐	☐	☐	☐
Other ☐	☐	☐	☐	☐	☐	☐

Q11: Principles of selecting conditions based on impacts

Note inserted: In the public version of survey, more than 70% of panellists strongly agreed that death should be taken into account when selecting conditions, and we are not asking you about this again. On the other hand, quality of life, physical disability and mental health were strongly agreed by more than 70% of professionals.

In addition, social deprivation factor is listed here to explore your views on whether it should be one of the determinants of condition selection for multimorbidity measurement. In other parts of the survey, a third of panellists voted to exclude social deprivation as a "condition" but there was considerable comment about its importance, including that it could be taken into account when selecting conditions for multimorbidity measurement.

Please rate the degree to which you agree or disagree with each of the following statements

Statement	Strongly disagree	Disagree	Neither agree nor disagree	Agree	Strongly agree	Don't know
Include conditions which significantly reduce quality of life	☐	☐	☐	☐	☐	☐

Round two public panel

Include conditions which cause significant physical disability	☐	☐	☐	☐	☐	☐
Include conditions which cause frailty (general physical and/or mental weakness and vulnerability)	☐	☐	☐	☐	☐	☐
Include conditions which significantly worsen mental health	☐	☐	☐	☐	☐	☐
Include conditions which significantly worsen how people perceive their general health	☐	☐	☐	☐	☐	☐
Include conditions which significantly increase treatment burden (the sum of all the hassles of taking medicines or attending appointments	☐	☐	☐	☐	☐	☐
Include conditions which could be impacted by or impact social deprivation and poverty	☐	☐	☐	☐	☐	☐
Other impact	☐	☐	☐	☐	☐	☐

Q12: Categorisation of conditions/counting methods

In round 1, we asked about how to account for complications of conditions and there was consensus (>70%) that all conditions that are currently active should be counted even if some are complications of other conditions. We are not asking this again. There was considerable free-text comment, including about the challenges of identifying specific conditions in electronic medical records. We have therefore revised the options for this question.

Please choose one option that you would prefer to use.

☐ Broad disease category based on body systems (e.g. cardiovascular disease, mental health problems, skin conditions).

☐ Individual conditions (e.g. myocardial infarction, angina, hypertension, depression, schizophrenia, eczema, psoriasis).

☐ Grouping together similar conditions that are in the same body system and treated similarly (e.g. angina and myocardial infarction, or aplastic anaemia and sickle cell anaemia)

Round two public panel

☐ Other. Please explain:

☒ Don't know

Q13: Multimorbidity has been measured by existing studies using either medical records or public self-reported surveys. Please rate the degree to which you agree or disagree the following statements.

Statement	Strongly disagree	Disagree	Neither agree nor disagree	Agree	Strongly agree	Don't know
Conditions included for multimorbidity measurement should be the same in both self-report and databases (e.g. identifying a core set of conditions or condition framework).	☐	☐	☐	☐	☐	☐
Conditions included in multimorbidity measures should differ between self-report and databases	☐	☐	☐	☐	☐	☐
Other	☐	☐	☐	☐	☐	☐

What conditions should be included?

Q14: System/disease domain

Note inserted: There was no consensus that which body systems should always be included in a multimorbidity measure.

Please rate the degree to which the following systems/domains, you think, are important.

Condition	Exclude (not importan t)	Usually exclude (unless a good reason to include in a particular context)	Could include or exclude	Usually include (unless a good reason to exclude in particular context)	Always include (extremely important)	Don't know
Mental health	☐	☐	☐	☐	☐	☐
Cancer	☐	☐	☐	☐	☐	☐
Cardiovascular	☐	☐	☐	☐	☐	☐
Blood disease (which affects formation of blood cells and blood clots)	☐	☐	☐	☐	☐	☐
Respiratory disease	☐	☐	☐	☐	☐	☐

Round two public panel

Neurological disease (which affects the nervous system)	☐	☐	☐	☐	☐	☐
Metabolic and Endocrine (which affects metabolic regulation. E.g. diabetes, thyroid problem)	☐	☐	☐	☐	☐	☐
Musculoskeletal (which affects joints, bones and muscles)	☐	☐	☐	☐	☐	☐
Digestive (which affects stomach, bowels, liver, pancreas and gallbladder)	☐	☐	☐	☐	☐	☐
Urogenital (which affects urinary and genital organs)	☐	☐	☐	☐	☐	☐
Skin	☐	☐	☐	☐	☐	☐
Ear, Nose and Throat (ENT)	☐	☐	☐	☐	☐	☐
Oral	☐	☐	☐	☐	☐	☐
Ophthalmology (which affects eyes)	☐	☐	☐	☐	☐	☐
Chronic infections	☐	☐	☐	☐	☐	☐

Note inserted: The number of conditions included in multimorbidity measurement varies substantially, which has led to the heterogeneity of multimorbidity prevalence. In the following section, we would like to ask you to rate a number of conditions that have been used for multimorbidity measurement, with the aim to identify a set of core conditions and conditions likely included for particular populations or regions.

Q15: Mental health

Note inserted: In mental health domain, none of the individual mental health conditions was rated by more than 70% of panellists as 'always include'. On the other hand, in the professional panel, there was consensus that dementia and schizophrenia should always be included in multimorbidity measurement.

Please rate the following mental health conditions as to whether they should be included or excluded, which allows us to see if your responses have changed between rounds. If you are not sure, please tick 'Don't know'.

Condition	Exclude (not important)	Usually exclude (unless a good reason to include in a particular context)	Could include or exclude	Usually include (unless a good reason to exclude in particular context)	Always include (extremely important)	Don't know
Anxiety	☐	☐	☐	☐	☐	☐
Depression	☐	☐	☐	☐	☐	☐

Round two public panel

Dementia	☐	☐	☐	☐	☐	☐
Schizophrenia	☐	☐	☐	☐	☐	☐
Substance use disorder(including alcohol and drug dependence)	☐	☐	☐	☐	☐	☐
Nicotine dependence	☐	☐	☐	☐	☐	☐
Post-traumatic stress disorder (PTSD)	☐	☐	☐	☐	☐	☐
Bipolar disorder	☐	☐	☐	☐	☐	☐
Chronic insomnia	☐	☐	☐	☐	☐	☐
Dissociative or personality disorders (altering to be a person with different personality and the alters are imaginary)	☐	☐	☐	☐	☐	☐
Eating disorders (including bulimia or anorexia)	☐	☐	☐	☐	☐	☐
Learning disability	☐	☐	☐	☐	☐	☐
Autism	☐	☐	☐	☐	☐	☐
Obsessive compulsive disorder (OCD)	☐	☐	☐	☐	☐	☐
Somatoform disorders (psychological disorder where a person experiences physical symptoms that cannot be explained by medical doctors)	☐	☐	☐	☐	☐	☐
Attention deficit hyperactivity disorder (ADHD)	☐	☐	☐	☐	☐	☐
Other (please specify either one or more conditions): _____________	☐	☐	☐	☐	☐	☐

Q16: In cancer domain, none of the individual cancer conditions was rated by more than 70% of panellists as ‘always include’. On the other hand, in the professional panel, there was consensus that solid organ cancers, haematological cancers and metastatic cancers should always be included in multimorbidity measurement. Please rate the following cancer

Round two public panel

conditions as to whether they should be included or excluded. If you are not sure, please tick 'Don't know'.

Condition	Exclude (not important)	Usually exclude (unless a good reason to include in a particular context)	Could include or exclude	Usually include (unless a good reason to exclude in particular context)	Always include (extremely important)	Don't know
Solid organ cancers (e.g. lung, colon, prostate, breast etc)	☐	☐	☐	☐	☐	☐
Haematological cancers (cancer that affects blood, e.g. leukaemia, lymphoma, myeloma)	☐	☐	☐	☐	☐	☐
Melanoma (serious form of malignant skin cancer)	☐	☐	☐	☐	☐	☐
Non-melanoma skin cancer	☐	☐	☐	☐	☐	☐
Benign cancers (excluding benign skin lumps and bumps)	☐	☐	☐	☐	☐	☐
Metastatic cancers (which have spread to other parts of the body)	☐	☐	☐	☐	☐	☐
Other (please specify either one or more conditions): ____________	☐	☐	☐	☐	☐	☐

Q17: Based on the panellists' responses, we have revised the options and would like to ask you to choose the method you would recommend.

☐ Count all cancers as one
☐ Count individual cancers separately irrespective of which systems they affect (e.g. count gastric cancer and liver cancer separately).
☐ Count individual cancers separately if they affect different systems (e.g. breast cancer and lung cancer).

☐ Other

Round two public panel

Q18: Note inserted: In cardiovascular domain, none of the individual cardiovascular conditions was rated by more than 70% of panellists as 'always include'. On the other hand, in the professional panel, there was consensus that stroke, coronary heart disease, heart failure and peripheral artery disease should always be included in multimorbidity measurement. Please rate the following cardiovascular conditions as to whether they should be included or excluded. If you are not sure, please tick 'Don't know'.

Condition	Exclude (not important)	Usually exclude (unless a good reason to include in a particular context)	Could include or exclude	Usually include (unless a good reason to exclude in particular context)	Always include (extremely important)	Don't know
Hypertension (treated)	☐	☐	☐	☐	☐	☐
High blood pressure (untreated)	☐	☐	☐	☐	☐	☐
Stroke	☐	☐	☐	☐	☐	☐
Transient Ischaemic Attack (mini stroke)	☐	☐	☐	☐	☐	☐
High cholesterol (treated)	☐	☐	☐	☐	☐	☐
High cholesterol (untreated)	☐	☐	☐	☐	☐	☐
Coronary artery disease (heart attack or angina)	☐	☐	☐	☐	☐	☐
Heart failure	☐	☐	☐	☐	☐	☐
Peripheral artery disease (circulation problems in the legs)	☐	☐	☐	☐	☐	☐
Heart rhythm problem (irregular or very fast heart beats)	☐	☐	☐	☐	☐	☐
Heart valves problem (leaking or tight heart valves)	☐	☐	☐	☐	☐	☐
Aneurysm (a weakening or bulging of an artery wall)	☐	☐	☐	☐	☐	☐
Other (please specify either one or more conditions): ____________	☐	☐	☐	☐	☐	☐

Round two public panel

Q19: Consistent with the results in Q14 that less than 70% of panellists would always include haematological system in multimorbidity measurement, none of the individual haematological conditions was rated by more than 70% of panellists as 'always include'.

Please rate the following haematological conditions as to whether they should be included or excluded. If you are not sure, please tick 'Don't know'.

Condition	Exclude (not important)	Usually exclude (unless a good reason to include in a particular context)	Could include or exclude	Usually include (unless a good reason to exclude in particular context)	Always include (extremely important)	Don't know
Anaemia	☐	☐	☐	☐	☐	☐
Venous thrombotic disease (blood clots formed inside a blood vessel that can block circulation)	☐	☐	☐	☐	☐	☐
Other (please specify either one or more conditions): ____________	☐	☐	☐	☐	☐	☐

Q20: Note inserted: In neurological domain, none of the individual neurological conditions was rated by more than 70% of panellists as 'always include'. On the other hand, in the professional panel, there was consensus that Parkinson's disease, epilepsy, multiple sclerosis, and paralysis/ hemiplegia/ paraplegia (not including those caused by stroke) should always be included in multimorbidity measurement.

Please rate the following neurological conditions as to whether they should be included or excluded. If you are not sure, please tick 'Don't know'.

Condition	Exclude (not important)	Usually exclude (unless a good reason to include in a particular context)	Could include or exclude	Usually include (unless a good reason to exclude in particular context)	Always include (extremel y important)	Don't know
Parkinson's disease	☐	☐	☐	☐	☐	☐
Epilepsy (fits/ seizures)	☐	☐	☐	☐	☐	☐
Migraine or other regular headache	☐	☐	☐	☐	☐	☐
Multiple sclerosis	☐	☐	☐	☐	☐	☐
Peripheral neuropathy (damage to the nerves going to legs or arms)	☐	☐	☐	☐	☐	☐
Paralysis/ Hemiplegia/ Paraplegia (not including those caused by stroke)	☐	☐	☐	☐	☐	☐

Round two public panel

Other (please specify either one or more conditions): _____________	☐	☐	☐	☐	☐	☐

Q21: Note inserted: In metabolic and endocrine domain, none of the individual conditions was rated by more than 70% of panellists as 'always include'. On the other hand, in the professional panel, there was consensus that diabetes (any types) should always be included in multimorbidity measurement.

Please rate the following metabolic and endocrine conditions as to whether they should be included or excluded. If you are not sure, please tick 'Don't know'.

Condition	Exclude (not important)	Usually exclude (unless a good reason to include in a particular context)	Could include or exclude	Usually include (unless a good reason to exclude in particular context)	Always include (extremely important)	Don't know
Diabetes (including type 1 and type 2)	☐	☐	☐	☐	☐	☐
Thyroid problems (abnormal production of thyroid hormones)	☐	☐	☐	☐	☐	☐
Malnutrition (including protein energy deficiency, commonly in low-income countries)	☐	☐	☐	☐	☐	☐
Addison's disease (adrenal insufficiency)	☐	☐	☐	☐	☐	☐
Other (please specify either one or more conditions): _____________	☐	☐	☐	☐	☐	☐

Q22: Note inserted: In musculoskeletal domain, none of the individual conditions was rated by more than 70% of panellists as 'always include'. On the other hand, in the professional panel, there was consensus that connective tissue disease (including rheumatoid arthritis or lupus) should always be included in multimorbidity measurement.

Please rate the following musculoskeletal conditions as to whether they should be included or excluded. If you are not sure, please tick 'Don't know'.

Condition	Exclude (not important)	Usually exclude (unless a good reason to include in a particular context)	Could include or exclude	Usually include (unless a good reason to exclude in particular context)	Always include (extremely important)	Don't know
Osteoporosis (thinning of the bones)	☐	☐	☐	☐	☐	☐

Round two public panel

Osteoarthritis (wearing and tearing of joints that leads to inflammation and degeneration	☐	☐	☐	☐	☐	☐
Connective tissue disease (autoimmune disease that can affect our bones, cartilages or joints, such as rheumatoid arthritis or lupus)	☐	☐	☐	☐	☐	☐
Gout (crystals that formed inside or around joints, causing pain)	☐	☐	☐	☐	☐	☐
Long-term musculoskeletal problems due to injury (including low back pain)	☐	☐	☐	☐	☐	☐
Other (please specify either one or more conditions): ____________	☐	☐	☐	☐	☐	☐

Q23: Note inserted: In respiratory domain, none of the individual conditions was rated by more than 70% of panellists as 'always include'. On the other hand, in the professional panel, there was consensus that COPD, asthma and cystic fibrosis should always be included in multimorbidity measurement.

Please rate the following respiratory condition as to whether it should be included or excluded. If you are not sure, please tick 'Don't know'.

Condition	Exclude (not important)	Usually exclude (unless a good reason to include in a particular context)	Could include or exclude	Usually include (unless a good reason to exclude in particular context)	Always include (extremely important)	Don't' know
Chronic obstructive pulmonary disease (COPD – smoking related lung damage)	☐	☐	☐	☐	☐	☐
Asthma	☐	☐	☐	☐	☐	☐
Sleep apnoea (breathing problems when asleep)	☐	☐	☐	☐	☐	☐
Cystic fibrosis (inherited condition that damages lungs)	☐	☐	☐	☐	☐	☐

Round two public panel

Chronic/Allergic rhinitis (long-term nasal inflammation)	☐	☐	☐	☐	☐	☐
Bronchiectasis (consistent infection in the lungs)	☐	☐	☐	☐	☐	☐
Post-acute COVID-19	☐	☐	☐	☐	☐	☐
Other (please specify either one or more conditions): ____________	☐	☐	☐	☐	☐	☐

Q24: Note inserted: In digestive domain, none of the individual conditions was rated by more than 70% of panellists as 'always include'. On the other hand, in the professional panel, there was consensus that chronic liver disease and inflammatory bowel disease should always be included in multimorbidity measurement.

Please rate the following digestive conditions as to whether they should be included or excluded. If you are not sure, please tick 'Don't know'.

Condition	Exclude (not important)	Usually exclude (unless a good reason to include in a particular context)	Could include or exclude	Usually include (unless a good reason to exclude in particular context)	Always include (extremely important)	Don't know
Chronic liver disease (including liver cirrhosis, liver failure and chronic hepatitis)	☐	☐	☐	☐	☐	☐
Inflammatory bowel disease (including ulcerative colitis and Crohn's disease)	☐	☐	☐	☐	☐	☐
Irritable bowel syndrome	☐	☐	☐	☐	☐	☐
Diverticulosis (small pockets in the lining of the intestine)	☐	☐	☐	☐	☐	☐
Pancreatic disease	☐	☐	☐	☐	☐	☐
Upper digestive disease— Peptic ulcer (including gastric/ stomach ulcers and duodenal ulcers) and Gastroesophageal reflux (acid reflux and heartburn)	☐	☐	☐	☐	☐	☐
Gall bladder problems (including gallstones)	☐	☐	☐	☐	☐	☐

Round two public panel

Other (please specify either one or more conditions): _____________	☐	☐	☐	☐	☐	☐

Q25: Note inserted: In urogenital domain, none of the individual conditions was rated by more than 70% of panellists as 'always include'. On the other hand, in the professional panel, there was consensus that chronic kidney disease and end-stage kidney disease should always be included in multimorbidity measurement.

Please rate the following urogenital conditions as to whether they should be included or excluded. If you are not sure, please tick 'Don't know'.

Condition	Exclude (not important)	Usually exclude (unless a good reason to include in a particular context)	Could include or exclude	Usually include (unless a good reason to exclude in particular context)	Always include (extremely important)	Don't know
Chronic kidney disease	☐	☐	☐	☐	☐	☐
End-stage kidney disease (including kidney dialysis and transplant)	☐	☐	☐	☐	☐	☐
Kidney or bladder stones	☐	☐	☐	☐	☐	☐
Chronic urinary tract infections (including chronic bladder infections)	☐	☐	☐	☐	☐	☐
Urinary incontinence (loss of control over passing urine)	☐	☐	☐	☐	☐	☐
Uterus (womb) problems (including prolapse and fibroid)	☐	☐	☐	☐	☐	☐
Polycystic ovary syndrome	☐	☐	☐	☐	☐	☐
Prostatic hypertrophy (large prostate glands)	☐	☐	☐	☐	☐	☐
Endometriosis	☐	☐	☐	☐	☐	☐
Infertility	☐	☐	☐	☐	☐	☐
Sexual dysfunction	☐	☐	☐	☐	☐	☐
Other (please specify either one or more conditions): _____________	☐	☐	☐	☐	☐	☐

Round two public panel

Q26: Note inserted: In chronic infections domain, none of the individual conditions was rated by more than 70% of panellists as 'always include'. On the other hand, in the professional panel, there was consensus that HIV/AIDS should always be included in multimorbidity measurement.

Please rate the following chronic infections conditions as to whether they should be included or excluded. If you are not sure, please tick 'Don't know'.

Condition	Exclude (not important)	Expect to usually exclude (unless a good reason to include in a particular context)	Could include or exclude	Expect to usually include (unless a good reason to exclude in particular context)	Always include (extremely important)
HIV/AIDS	☐	☐	☐	☐	☐
Tuberculosis	☐	☐	☐	☐	☐
Lyme disease (a bacterial infection that can be spread to humans by infected ticks)	☐	☐	☐	☐	☐
Other (please specify either one or more conditions): ____________	☐	☐	☐	☐	☐

Q27: Note inserted: In skin domain, none of the individual conditions was rated by more than 70% of panellists as 'always include', in both members of the public panel and professional panel.

Please rate the following skin conditions as to whether they should be included or excluded again. If you are not sure, please tick 'Don't know'.

Condition	Exclude (not important)	Usually exclude (unless a good reason to include in a particular context)	Could include or exclude	Usually include (unless a good reason to exclude in particular context)	Always include (extremely important)	Don't know
Psoriasis	☐	☐	☐	☐	☐	☐
Eczema	☐	☐	☐	☐	☐	☐
Chronic urticarial (chronic hives)	☐	☐	☐	☐	☐	☐
Other (please specify either one or more conditions): ____________	☐	☐	☐	☐	☐	☐

Round two public panel

Q28: Note inserted: In ENT, eye and oral domains, none of the individual conditions was rated by more than 70% of panellists as 'always include', in both members of the public panel and professional panel.

Please rate the following ENT, eye and oral conditions as to whether they should be included or excluded again. If you are not sure, please tick 'Don't know'.

Condition	Exclude (not important)	Usually exclude (unless a good reason to include in a particular context)	Could include or exclude	Usually include (unless a good reason to exclude in particular context)	Always include (extremely important)	Don't know
Hearing impairment or Deafness (that cannot be easily corrected with hearing aids)	☐	☐	☐	☐	☐	☐
Meniere's disease (an ear condition that causes sudden attacks of vertigo)	☐	☐	☐	☐	☐	☐
Chronic sinusitis (sinus infection)	☐	☐	☐	☐	☐	☐
Vision impairment or Blindness (that cannot be easily corrected with glasses)	☐	☐	☐	☐	☐	☐
Cataract	☐	☐	☐	☐	☐	☐
Glaucoma	☐	☐	☐	☐	☐	☐
Edentulism (having no teeth)	☐	☐	☐	☐	☐	☐
Chronic gum disease	☐	☐	☐	☐	☐	☐
Other (please specify either one or more conditions): ___________	☐	☐	☐	☐	☐	☐

Q29: Note inserted: In congenital disease domain, none of the individual conditions was rated by more than 70% of panellists as 'always include', in both members of the public panel and professional panel.

Please rate the following statement as to whether congenital conditions should be included or excluded again. If you are not sure, please tick 'Don't know'.

Condition	Exclude (not important)	Usually exclude (unless a	Could include or exclude	Usually include (unless a	Always include	Don't know

Round two public panel

		good reason to include in a particular context)		good reason to exclude in particular context)	(extremely important)	
Congenital disease (conditions that babies are born with, including congenial heart disease, chromosomal aberrations)	☐	☐	☐	☐	☐	☐
Other (please specify either one or more conditions): ____________	☐	☐	☐	☐	☐	☐

Q30: Note inserted: The following risk factors/symptoms have been included in multimorbidity measures by existing studies, but none of them was rated by more than 70% of panellists as 'always include'. Some suggested that those should be measured separately rather than as part of a multimorbdiity measure.

Having read a summary of the results from round one, please rate the following statement as to whether these conditions should be included or excluded again. If you are not sure, please tick 'Don't know'.

Condition	Exclude (not important)	Usually exclude (unless a good reason to include in a particular context)	Could include or exclude	Usually include (unless a good reason to exclude in particular context)	Always include (extremely important)	Don't know
Obesity (body mass index ≥30)	☐	☐	☐	☐	☐	☐
Smoking	☐	☐	☐	☐	☐	☐
Sedentary lifestyle (spending most of the day sitting)	☐	☐	☐	☐	☐	☐
Physical disability	☐	☐	☐	☐	☐	☐
Dizziness (without a specific diagnosis)	☐	☐	☐	☐	☐	☐
Chronic cough (without a specific diagnosis)	☐	☐	☐	☐	☐	☐
Post-sepsis syndrome	☐	☐	☐	☐	☐	☐
Side effects of medications	☐	☐	☐	☐	☐	☐
Treatment burden (the sum of all the hassles of taking medicines or attending appointments)	☐	☐	☐	☐	☐	☐

Round two public panel

Social deprivation and poverty	☐	☐	☐	☐	☐	☐
Other (please specify either one or more conditions): _____________	☐	☐	☐	☐	☐	☐

Weighting

Q31:

Reflecting on a summary of the results from round one and your responses so far, please rate the degree to which these outcomes, you think, are important to be considered when investigating the impact of multimorbidity.

Outcome	Not at all important	Slightly important	Important	Sufficiently important	Very important	No opinion
Death	☐	☐	☐	☐	☐	☐
Healthcare use (e.g. number of emergency admissions to hospital; outpatient appointments)	☐	☐	☐	☐	☐	☐
Quality of life	☐	☐	☐	☐	☐	☐
Physical disability	☐	☐	☐	☐	☐	☐
Frailty (general physical and/or mental weakness and vulnerability)	☐	☐	☐	☐	☐	☐
Treatment burden (the sum of all the hassles of taking medicines or attending appointments)	☐	☐	☐	☐	☐	☐
Healthcare costs (how much treatment and care for each individual costs)	☐	☐	☐	☐	☐	☐
How people perceive their general health overall (e.g. whether it is excellent, very good, fair, poor, very poor)	☐	☐	☐	☐	☐	☐
Other (please specify)	☐	☐	☐	☐	☐	☐
Other (please specify)	☐	☐	☐	☐	☐	☐

Round three public panel

Round-three survey in the public panel

Socio-demographic information

Q1: Please describe the country where you are currently living.

[ ]

Q2: What is your age?

- ☐ 18-34
- ☐ 35-54
- ☐ 55-64
- ☐ ≥ 65
- ☐ Prefer not to answer

Q3: What gender do you identify as?

- ☐ Female
- ☐ Male
- ☐ Other __________
- ☐ Prefer not to answer

Q4: We are seeking your views as a member of the public, but we know that some of you will have worked in healthcare or academia. Do you currently or did you previously work in academia, health and social care practice or healthcare policy?

- ☐ Yes
- ☐ No

Q5: Do you have multiple chronic conditions?

- ☐ Yes
- ☐ No

Q6: Do you have family or friends who have multiple chronic conditions?

- ☐ Yes
- ☐ No

What is multimorbidity?

Q7: Please rate the degree to which you agree or disagree with the following statement again so we can see if there is a change between round 2 and round 3.

	Strongly disagree	Disagree	Neither disagree nor agree	Agree	Strongly agree	Don't know

Round three public panel

Do you agree that defining complex multimorbidity in addition to a core definition of simple multimorbidity is useful?	☐	☐	☐	☐	☐	☐

Q8: Irrespective of whether you agree that complex multimorbidity is a useful concept, we would like to invite you to define "complex multimorbidity" based on number of conditions (please choose one).

☐ 3+ conditions irrespective of how many body systems
☐ 3+ conditions from 2+ body systems
☐ 3+ conditions from 3+ body systems
☐ 4+ conditions irrespective of how many body systems
☐ No opinion
☐ Other

Note: In the public and professional panel, there was no consensus on what additional patterns of conditions should be included in the definition. We have added a few more statements based on panellists' comments.

Sub-question: Please choose the following statements that, you think, should be included in the definition of "complex multimorbidity".

☐ No other patterns that I would like to include
☐ Any combination of two or more conditions which includes both physical and mental health conditions
☐ Any combination of two or more conditions with significant physical functional limitation
☐ Difficulty in managing illnesses due to social factors/social determinants of health (e.g. poverty).
☐ Any combination of two or more conditions and frailty
☐ Other

Q9. Recurrence or remission

Please rate the degree to which you agree or disagree with the following statements.

Statement	Strongly disagree	Disagree	Neither disagree nor agree	Agree	Strongly agree	Don't know

Round three public panel

Include remitting-relapsing conditions that require ongoing treatment/ therapy/ care (e.g. depression, epilepsy)—newly added	☐	☐	☐	☐	☐	☐
Include remitting-relapsing conditions which have relapsed during the last five years	☐	☐	☐	☐	☐	☐

Q10: Treatment, care or surveillance

Note inserted: More than 70% of panellists strongly agreed to include conditions that require current treatment, care, or therapy, and this question will not be asked again in round three. Please rate the degree to which you agree or disagree with the following statements.

Statement	Strongly disagree	Disagree	Neither disagree nor agree	Agree	Strongly agree	Don't know
Include conditions which usually require treatment, care or therapy 'at some point in the future' even if not currently treated (one panellist suggested to conceptualise this as 'current risk to future health outcomes')	☐	☐	☐	☐	☐	☐
Include conditions which usually require surveillance (one panellist suggested to conceptualise this as 'current health needs or complexity of providing care')	☐	☐	☐	☐	☐	☐

Q11: Categorisation of conditions/counting methods

Please choose one option that you would prefer to use.

☐ Broad disease category based on body and mind system (e.g. cardiovascular disease, mental health problems, skin conditions).

☐ Individual conditions (e.g. TIA (mini stroke) and stroke are counted separately).

☐ Grouping together similar conditions that are in the same category and treated similarly (e.g. grouping together 'Angina and Myocardial Infarction')

☐ Other. Please explain:

Round three public panel

☒ Don't know

Q12: Data source

Please rate the following statements again.

Statement	Strongly disagree	Disagree	Neither disagree nor agree	Agree	Strongly agree	Don't know
Conditions included for multimorbidity measurement should be the same/similar in both self-report and databases (e.g. identifying a core set of conditions or condition framework).	☐	☐	☐	☐	☐	☐
Other comments	☐	☐	☐	☐	☐	☐

What conditions should be included?

Note inserted: In the previous section, more than 70% of panellists have agreed to include formal medical diagnoses, clinical risk factors, conditions that are permanent in their effects, conditions that last 12 months or longer, and require current treatment, care, or therapy.

Q13: Note inserted: In cancer conditions, we added three new statements based on panellists' comments. Please rate the following cancer conditions as to whether they should be included or excluded. If you are not sure, please tick 'Don't know'.

Condition	Exclude (not important)	Usually exclude (unless a good reason to include in a particular context)	Could include or exclude	Usually include (unless a good reason to exclude in particular context)	Always include (extremely important)	Don't know
Treated cancer that requires surveillance	☐	☐	☐	☐	☐	☐
Treated cancer that did not recur over the past 5 years and does not require surveillance	☐	☐	☐	☐	☐	☐
Benign cerebral tumours (brain tumours that can cause functional limitations)	☐	☐	☐	☐	☐	☐

Round three public panel

Q14: Please choose the categorisation method you would prefer again.

☐ Count all primary cancers as one
☐ Count individual primary cancers separately irrespective of which systems they affect (e.g. count gastric cancer and liver cancer separately).
☐ Count individual primary cancers separately if they affect different systems (e.g. pancreatic cancer and lung cancer).
☐ Other

Q15: Conditions relevant to chronic pain and consequences of injury were revised based on panellists' comments. Please rate the following conditions as to whether they should be included or excluded. If you are not sure, please tick 'Don't know'.

Condition	Exclude (not important)	Usually exclude (unless a good reason to include in a particular context)	Could include or exclude	Usually include (unless a good reason to exclude in particular context)	Always include (extremel y important)	Don't know
Chronic or recurrent low back pain	☐	☐	☐	☐	☐	☐
Chronic primary pain (defined as pain with no clear underlying condition but significant impact on the person)	☐	☐	☐	☐	☐	☐
Long-term musculoskeletal problems due to injury (e.g. consequences of accidental injuries)	☐	☐	☐	☐	☐	☐

Q16: Conditions newly added in round two

Please rate again the following conditions so we can see if your responses have changed between round two and round three

Condition	Exclude (not important)	Usually exclude (unless a good reason to include in a particular context)	Could include or exclude	Usually include (unless a good reason to exclude in particular context)	Always include (extremel y important)	Don't know
Addison's disease (adrenal insufficiency, which is an uncommon	☐	☐	☐	☐	☐	☐

Round three public panel

disorder which is fatal without lifelong treatment)						
Bronchiectasis (a lung condition that causes cough, sputum production, and recurrent respiratory infections)	☐	☐	☐	☐	☐	☐
Post-acute covid 19 ("long COVID")	☐	☐	☐	☐	☐	☐
Chronic Lyme disease (a bacterial infection that can be spread to humans by infected ticks)	☐	☐	☐	☐	☐	☐
