# Supplementary material for: Measuring multimorbidity in research: Delphi consensus study
Source: BMJ Med. 2022 Jul 27;1(1):e000247. doi: 10.1136/bmjmed-2022-000247 (PMC9978673; doi:10.1136/bmjmed-2022-000247)
Supplement: Supplementary data [file bmjmed-2022-000247supp003.pdf]

Measuring multimorbidity in research: a Delphi consensus study

Appendix 3

Table of Contents

Figure S1: Criteria for defining consensus of items ... 2

Box 1: Rasch Model used for sensitivity analysis ... 3

Table S1: Characteristics of non-respondents and unidentified panelists ... 4

Table S2: Responses to questions on conceptualising multimorbidity ... 6

Figure S2: Type of conditions to include when defining multimorbidity ... 8

Table S3: Defining “condition” in the concept of multimorbidity (based on participant free-text comments, questions changed from round to round) ... 9

Table S4: Consideration of conditions to include based on impact (based on participant free-text comments, questions changed from round to round) ... 10

Table S5: Responses to questions relevant to weighting (based on participant free-text comments, questions changed from round to round) ... 11

Table S6: Professional ratings for choice of simple counts vs weighted measures depending on the purpose of a study ... 13

Table S7: Professional ratings for choice of simple counts vs weighted measures for outcome prediction depending on the outcome being measured ... 14

Table S8: Professional scores for ‘always include’ ... 15

Table S9: Public scores for ‘always include’ ... 17

Table S10: Professional scores for ‘usually include’ (the sum of ‘always’ and ‘usually’) ... 19

Table S11: Public scores for ‘usually include’ (the sum of ‘always’ and ‘usually’) ... 21

Table S12: Conditions not recommended to ‘always include’ or ‘usually include’ by both panels ... 23

Table S13: Differential item functioning (used to examine which condition-choice items were more likely to be endorsed by panelists with varying interest in different populations [4]) ... 24

Table S14: Level of endorsement of the condition-related items ... 26

References ... 30

Figure S1: Criteria for defining consensus of items

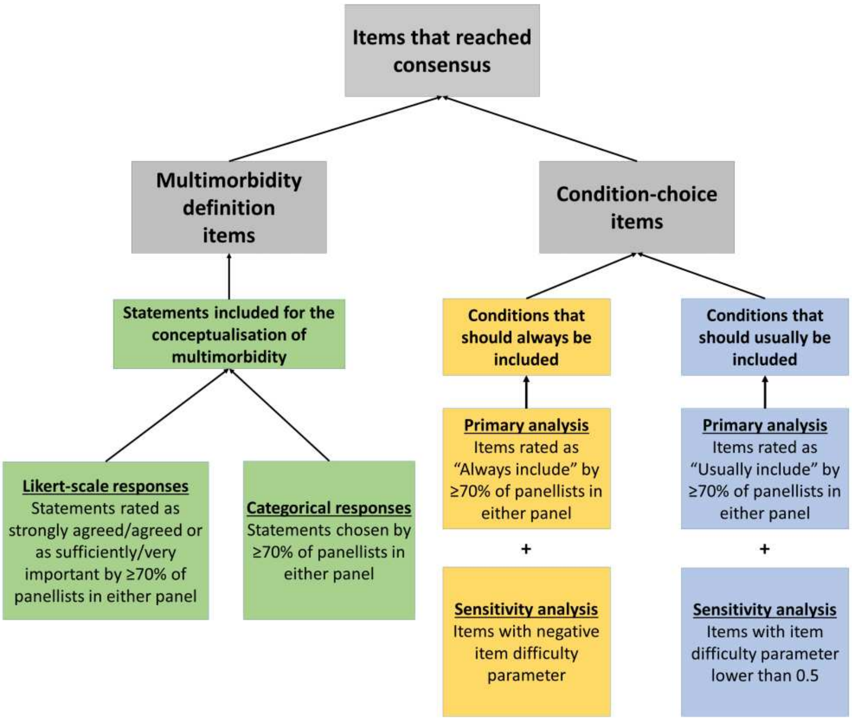

Box 1: Rasch Model used for sensitivity analysis

The Rasch dichotomous model is an item response theory model with one item parameter [1]. We used Rasch modelling to examine the degree to which condition-relevant items were endorsed by panelists. The common feature in the Rasch model is conditional maximum likelihood estimation which leads to the separability of item and person parameters [2]. Item discrimination (the level of endorsement varies with person ability) in the Rasch analysis is fixed for all items [3].

The mathematical form of the Rasch dichotomous model is given as follows[1, 3]:

$P(X_{is} = 1|\theta s , \beta i) = e(\theta s-\beta i)/1 + e(\theta s-\beta i)$
X_{is} refers to response (*X*) made by subject *s* to item *i.*
X_{is} = 1 refers to an endorsement of the item
βi refers to the difficulty of item *i*
θ refers to person's trait/ability
To estimate the trait level, we can take the natural log of a ratio of proportion endorsed responses to proportion unendorsed responses:
$\theta = LN(Ps/1 - Ps)$
To estimate item difficulty, we can take the natural log of a ratio of proportion unendorsed responses to proportion endorsed responses.
$\beta = LN(1 - Ps /Ps)$

Table S1: Characteristics of non-respondents and unidentified panelists‡

Characteristics	Professional round one participant who did not respond or could not be tracked in round two (n=42)[a] No (%)	Professional round two participant who did not respond in round three Professional round two (n=15)[b] No (%)	Public round one participant who did not respond or could not be tracked in round two (n=10)[c] No (%)	Public round two participant who did not respond or could not be tracked in round three (n=7)[d] No (%)
**Continent**				
Europe	24 (57.1%)	9 (60.0%)	7 (70.0%)	7 (100.0%)
North America	7 (16.7%)	2 (13.3%)	1 (10.0%)	0
Australasia	1 (2.4%)	2 (13.3%)	0	0
Asia	9 (21.4%)	1 (6.7%)	1 (10.0%)	0
South America	1 (2.4%)	0	0	0
Africa	0	1 (6.7%)	0	0
Not stated	0	0	1 (10.0%)	0
**Country income**				
High-income	39 (92.9%)	14 (93.3%)	8 (80.0%)	7 (100.0%)
Low and middle income	3 (7.1%)	1 (6.7%)	1 (10.0%)	0
Not stated	0	0	1 (10.0%)	0
**Participant has multimorbidity**				
Yes	2 (4.8%)	3 (20.0%)	13 (52.0%)	19 (61.3%)
No	40 (95.2%)	12 (80.0%)	12 (48.0%)	12 (38.7%)
Not stated	0	0	0	0
**Family or friends have multimorbidity**				
Yes	28 (66.7%)	10 (66.7%)	6 (60.0%)	5 (71.4%)
No	14 (33.3%)	5 (33.3%)	4 (40.0%)	2 (28.6%)
**Type of work***				
Research	31 (73.8%)	13 (86.7%)	-	-
Public policy	4 (9.5%)	2 (13.3%)	-	-
Clinical practice	16 (38.1%)	4 (26.7%)	-	-
Teaching	2 (4.8%)	0	-	-
**Main work setting**				
Government	4 (9.5%)	0	-	-
Academia	26 (61.9%)	13 (86.7%)	-	-
Hospital	2 (4.8%)	0	-	-
Primary care	10 (23.8%)	2 (13.3%)	-	-
Other	0	0	-	-
**Populations of interest***				
General population	27 (64.3%)	11 (73.3%)	-	-
Older people	29 (69.0%)	7 (46.7%)	-	-
Middle-aged and older	26 (61.9%)	6 (40.0%)	-	-
Socially-deprived population	23 (54.8%)	6 (40.0%)	-	-
Women	10 (23.8%)	2 (13.3%)	-	-
Men	10 (23.8%)	1 (6.7%)	-	-
Children	5 (11.9%)	1 (6.7%)	-	-
Ethnic minority/indigenous	1 (2.4%)	3 (20.0%)	-	-
People with disability	0	2 (13.3%)	-	-
**Age group**				
18-34	-	-	1 (10.0%)	0
35-54	-	-	2 (20.0%)	2 (28.6%)
55-64	-	-	3 (30.0%)	1 (14.3%)
≥65	-	-	4 (40.0%)	4 (57.1%)

Characteristics	Professional round one participant who did not respond or could not be tracked in round two (n=42)[a] No (%)	Professional round two participant who did not respond in round three Professional round two (n=15)[b] No (%)	Public round one participant who did not respond or could not be tracked in round two (n=10)[c] No (%)	Public round two participant who did not respond or could not be tracked in round three (n=7)[d] No (%)
**Gender**				
Female	-	-	4 (40.0%)	6 (85.7%)
Male	-	-	6 (60.0%)	1 (14.3%)

ǂ"Unidentified panelists" is defined as "panelists who did not provide their email or preferred name in the previous and following rounds". Some who dropped out in the following round did not provide their email and preferred name either. Thus, they were in the pool of those who either dropped out or cannot be identified". As we couldn't distinguish these two groups in the pool, we reported the characteristics of the two groups together.
*multiple select question
a. The 42 panelists consist of 38 who dropped out in round 2, and 4 are unidentified.
b. The 15 non-respondents in round 2
c. The 10 panelists consist of 6 who dropped out in round 2 and 4 are unidentified
d. The 7 panelists consist of 6 who dropped out in round 3 and 1 are unidentified

Table S2: Responses to questions on conceptualising multimorbidity

Question	Choice	Professional panel			Public panel		
		**Round 1 (n=150)**	**Round 2 (n=112)**	**Round 3 (n=97)**	**Round 1 (n=25)**	**Round 2 (n=31)**	**Round 3 (n=25)**
Number of conditions to define multimorbidity	**2 or more**<br>3 or more<br>4 or more<br>5 or more<br>Other	68.3%<br>29.3%<br>1.3%<br>0.7%<br>0.7%	**84.8%**<br>12.5%<br>0.9%<br>0<br>1.8%	-	**88.0%**<br>8.0%<br>0<br>4.0%<br>0	-	-
How long-term a condition should be	3 months or more<br>**6 months or more**<br>**12 months or more**<br>Other	16.7%<br>42.7%<br>33.3%<br>7.3%	3.6%<br>**70.5%**<br>23.2%<br>2.7%	-	8.0%<br>8.0%<br>**76.0%**<br>8.0%	-	-
Do you agree that defining complex multimorbidity in addition to a core definition of simple multimorbidity is useful?	**Strongly agree**<br>**Agree**<br>Neither agree nor disagree<br>Disagree<br>Strongly disagree	-	**36.4%**<br>**32.7%**<br>9.1%<br>15.5%<br>6.4%	**60.4%**<br>**27.1%**<br>4.2%<br>5.2%<br>3.1%	-	**33.3%**<br>**53.3%**<br>3.3%<br>6.7%<br>3.3%	**36.0%**<br>**48.0%**<br>4.0%<br>4.0%<br>8.0%
How would you define complex multimorbidity based on number of conditions?	3 or more conditions irrespective of how many body systems<br>3 or more conditions from two or more body systems<br>3 or more conditions from three or more body systems<br>4 or more conditions irrespective of how many body systems<br>4 or more conditions four or more body systems<br>5 or more conditions irrespective of how many body systems<br>5 or more conditions five or more body systems<br>Other/No opinion	-	17.9%<br>29.5%<br>14.3%<br>6.3%<br>1.8%<br>3.6%<br>1.8%<br>25.0%	25.8%<br>33.0%<br>26.8%<br>3%<br>0<br>0<br>0<br>11.3%	-	41.9%<br>16.1%<br>19.4%<br>9.7%<br>0<br>0<br>0<br>12.9%	**76.0%**<br>12.0%<br>12.0%<br>0<br>0<br>0<br>0<br>0
Other patterns I would like to include in the definition of complex multimorbidity*	No other patterns that I would like to include<br>Any combination of two or more conditions which includes both physical and mental health conditions<br>Any combination of two or more conditions with significantly physical functional limitation<br>Difficulty in managing illnesses due to social factors/social determinants of health<br>Any combination of two or more conditions and frailty	-	29.5%<br>36.6%<br>0<br>0<br>0	22.7%<br>33.0%<br>30.9%<br>26.8%<br>25.8%	-	16.1%<br>64.5%<br>0<br>0<br>0	20.0%<br>44.0%<br>32.0%<br>28.0%<br>12.0%
What kind of conditions to include*	**Formal medical diagnosis**<br>**Clinical risk factor**<br>Symptom	**98.7%**<br>54.0%<br>22.7%	**99.1%**<br>49.1%<br>20.5%	-	**96.0%**<br>60.0%<br>28.0%	**96.8%**<br>**74.2%**<br>58.1%	-

	Health behaviour (e.g. smoking)<br>Health impact (e.g. disability)<br>Social deprivation and poverty<br>Consequences of treatment<br>Environmental factors	11.3%<br>39.3%<br>15.3%<br>22.7%<br>-	8.9%<br>36.6%<br>10.7%<br>21.4%<br>4.5%		16.0%<br>40.0%<br>16.0%<br>40.0%<br>-	51.6%<br>25.8%<br>38.7%<br>29.0%<br>22.6%	
How to count conditions (round 1)	Count broad disease category based on body and mind system<br>**Count individual conditions**<br>Other	23.1%<br>**72.0%**<br>4.9%	-	-	12.0%<br>**88.0%**<br>0	-	-
How to count conditions (round 2)	**Count all of the conditions that are currently active**<br>Count the primary health condition<br>Only count the complications	**84.0%**<br>4.0%<br>2.0%	-	-	**80.0%**<br>16.0%<br>0	-	-
How to count cancers (round 1)	Count all cancers as one<br>Count individual cancers separately<br>Other	25.3%<br>**62.0%**<br>12.7%	-	-	24%<br>**76%**<br>0	-	-
How to count cancers (round 2)	Count all cancers as one<br>Count individual cancers separately irrespective of which systems they affect<br>Count individual cancers separately if they affect different systems<br>Other	-	14.3%<br>38.4%<br>45.5%<br>1.8%	-	-	16.1%<br>38.7%<br>45.2%<br>0	-
How to count cancers (round 3)	Count all primary cancers as one<br>Count individual primary cancers separately irrespective of which systems they affect<br>Count individual primary cancers separately only if they affect different systems<br>Other	-	-	11.3%<br>55.7%<br>33.0%<br>0	-	-	12.0%<br>48.0%<br>36.0%<br>4.0%
Conditions included for multimorbidity measurement should be the same/similar in both self-report and databases	**Strongly agree**<br>**Agree**<br>Neither agree nor disagree<br>Disagree<br>Strongly disagree	-	**32.1%**<br>**41.3%**<br>11.9%<br>11.9%<br>2.8%	**29.1%**<br>**42.7%**<br>5.5%<br>7.3%<br>1.8%	-	**36.7%**<br>**60.0%**<br>3.3%<br>0<br>0	**44.0%**<br>**52.0%**<br>4.0%<br>0<br>0

* multiple select question

Figure S2: Type of conditions to include when defining multimorbidity

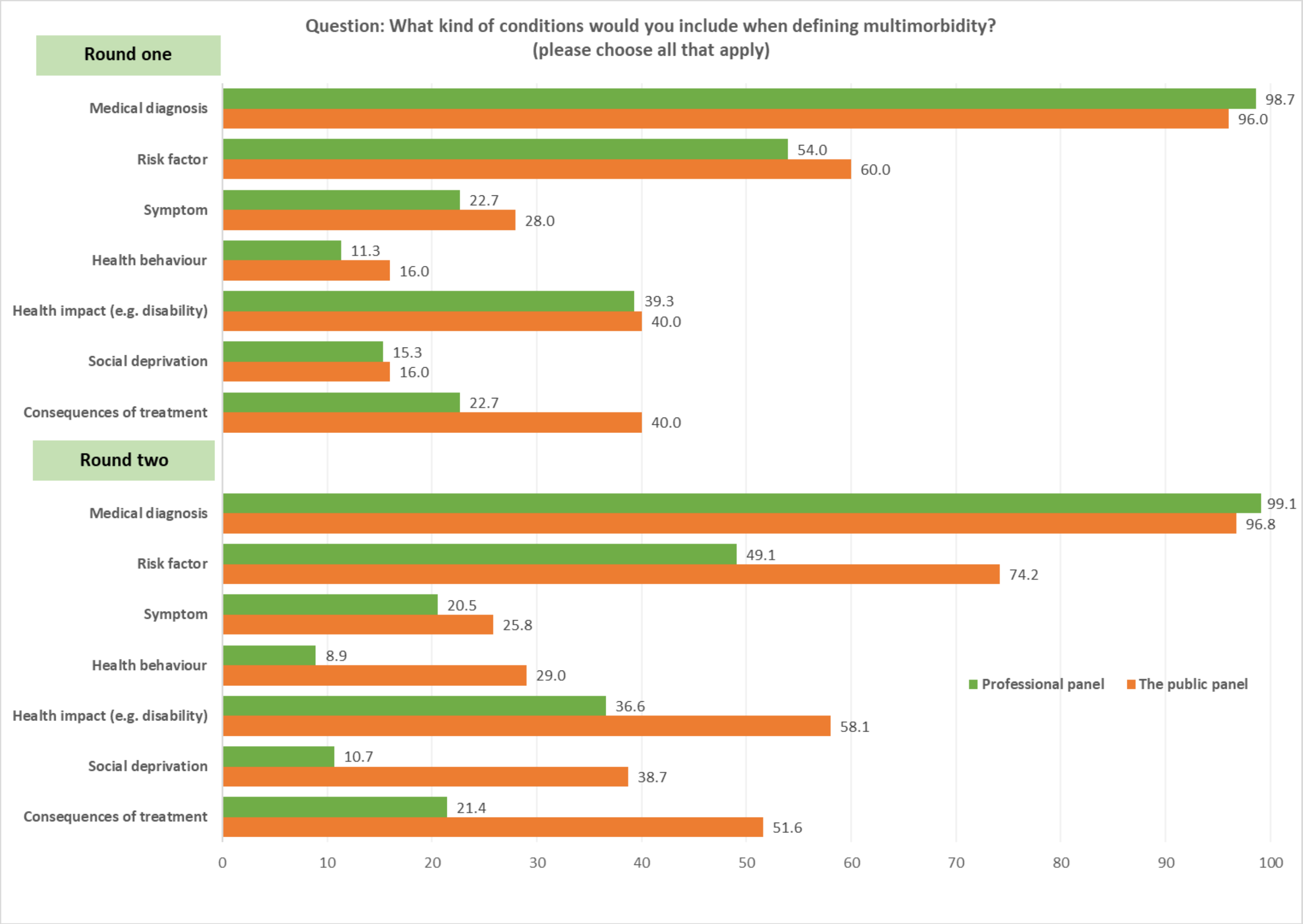

Table S3: Defining "condition" in the concept of multimorbidity (questions changed from round to round based on participant free-text feedback)

Statement	Rating	Professional panel			Public panel		
		Round 1 (n=150)	Round 2 (n=112)	Round 3 (n=97)	Round 1 (n=25)	Round 2 (n=31)	Round 3 (n=25)
Include conditions which are permanent in their effects	**Strongly agree**<br>**Agree**<br>Neither agree/disagree<br>Disagree<br>Strongly disagree	**89.9%**<br>**8.7%**<br>0<br>0<br>1.3%			**80.0%**<br>**16.0%**<br>0<br>0<br>4.0%		
Include conditions which are currently active	**Strongly agree**<br>**Agree**<br>Neither agree/disagree<br>Disagree<br>Strongly disagree	**73.2%**<br>**25.5%**<br>0<br>0.7%<br>0.7%			**60.0%**<br>**28.0%**<br>8.0%<br>0<br>4.0%	**64.5%**<br>**29.0%**<br>3.2%<br>0<br>3.2%	
Include conditions which may recur but happen rarely	Strongly agree<br>Agree<br>Neither agree/disagree<br>Disagree<br>Strongly disagree	13.6%<br>25.9%<br>19.7%<br>35.4%<br>5.4%			28.0%<br>16.0%<br>36.0%<br>20.0%<br>0		
Include conditions which may recur or remit	Strongly agree<br>Agree<br>Neither agree/disagree<br>Disagree<br>Strongly disagree		27.3%<br>48.2%<br>14.5%<br>7.3%<br>2.7%			41.9%<br>45.2%<br>9.7%<br>0<br>3.2%	
Include remitting-relapsing conditions which have relapsed during the last five years	Strongly agree<br>Agree<br>Neither agree/disagree<br>Disagree<br>Strongly disagree		23.4%<br>56.1%<br>10.3%<br>10.3%<br>0	12.9%<br>48.4%<br>22.6%<br>15.1%<br>1.1%		43.3%<br>40.0%<br>10.0%<br>3.3%<br>3.3%	21.0%<br>37.5%<br>37.5%<br>0<br>4.2%
Include remitting-relapsing conditions that require ongoing treatment/ therapy/ care	**Strongly agree**<br>**Agree**<br>Neither agree/disagree<br>Disagree<br>Strongly disagree			**49.0%**<br>**44.8%**<br>2.1%<br>3.1%<br>1.0%			**52.0%**<br>**40.0%**<br>0<br>0<br>8.0%
Only include conditions which usually require formal treatment or care	Strongly agree<br>Agree<br>Neither agree/disagree<br>Disagree<br>Strongly disagree	13.4%<br>31.5%<br>14.1%<br>32.9%<br>8.1%			28.0%<br>20.0%<br>8.0%<br>28.0%<br>16.0%		
Include conditions which usually require 'current' treatment, care or therapy	**Strongly agree**<br>**Agree**<br>Neither agree/disagree<br>Disagree<br>Strongly disagree		**77.5%**<br>**22.5%**<br>0<br>0<br>0			**71.0%**<br>**25.8%**<br>0<br>0<br>3.2%	
Include conditions which usually require treatment, care or therapy 'at some point in the future' even if not currently treated	Strongly agree<br>Agree<br>Neither agree/disagree<br>Disagree<br>Strongly disagree		36.7%<br>38.5%<br>11.9%<br>12.8%<br>0	13.7%<br>43.2%<br>23.2%<br>17.9%<br>2.1%		27.6%<br>51.7%<br>10.3%<br>3.4%<br>6.9%	24.0%<br>44.0%<br>20.0%<br>8.0%<br>4.0%
Include conditions which usually require surveillance	Strongly agree<br>Agree<br>Neither agree/disagree<br>Disagree<br>Strongly disagree		38.0%<br>37.0%<br>11.1%<br>13.0%<br>0.9%	**20.0%**<br>**54.7%**<br>12.6%<br>11.6%<br>1.1%		41.9%<br>45.2%<br>9.7%<br>0<br>3.2%	**32.0%**<br>**56.0%**<br>8.0%<br>0<br>4.0%

Percentages were rounded.
Number of panelists voting "No opinion/Don't know" was not added in the denominator when calculating percentages

Table S4: Consideration of conditions to include based on impact (based on participant free-text comments, questions changed from round to round)

Statement	Rating	Professional panel		Public panel	
		Round one (n=150)	Round two (n=112)	Round one (n=25)	Round two (n=31)
Include conditions which significantly increase risk of death	**Strongly agree**	**68.7%**	**69.4%**	**80.0%**	-
	**Agree**	**20.4%**	**25.2%**	**20.0%**	
	Neither agree nor disagree	5.4%	2.7%	0	
	Disagree	4.8%	0.9%	0	
	Strongly disagree	0.7%	1.8%	0	
Include conditions which significantly reduce quality of life	**Strongly agree**	**76.5%**	-	**60.0%**	**67.7%**
	**Agree**	**20.1%**		**28.0%**	**25.8%**
	Neither agree nor disagree	2.7%		8.0%	3.2%
	Disagree	0.7%		4.0%	0
	Strongly disagree	0		0	3.2%
Include conditions which cause significant physical disability	**Strongly agree**	**69.8%**	-	**68.0%**	**64.5%**
	**Agree**	**23.5%**		**24.0%**	**32.3%**
	Neither agree nor disagree	5.4%		8.0%	0
	Disagree	1.3%		0	0
	Strongly disagree	0		0	3.2%
Include conditions which cause frailty	**Strongly agree**	**65.8%**	**61.5%**	**44.0%**	**51.6%**
	**Agree**	**24.8%**	**28.4%**	**40.0%**	**38.7%**
	Neither agree nor disagree	7.4%	5.5%	16.0%	6.5%
	Disagree	2.0%	1.8%	0	0
	Strongly disagree	0	2.8%	0	3.2%
Include conditions which significantly worsen mental health	**Strongly agree**	**71.8%**	-	**62.5%**	**64.5%**
	**Agree**	**20.8%**		**16.7%**	**22.6%**
	Neither agree nor disagree	6.0%		20.8%	9.7%
	Disagree	1.3%		0	0
	Strongly disagree	0		0	3.2%
Include conditions which significantly worsen self-perceived health status	**Strongly agree**	**49.7%**	**48.6%**	17.4%	16.7%
	**Agree**	**28.9%**	**28.8%**	43.5%	46.7%
	Neither agree nor disagree	17.4%	16.2%	26.1%	30.0%
	Disagree	4.0%	4.5%	13.0%	0
	Strongly disagree	0	1.8%	0	6.7%
Include conditions which significantly increase treatment burden	**Strongly agree**	-	**55.0%**	-	**51.6%**
	**Agree**		**32.4%**		**35.5%**
	Neither agree nor disagree		7.2%		9.7%
	Disagree		3.6%		0
	Strongly disagree		1.8%		3.2%
Include conditions which could be impacted by or impact social deprivation and poverty	Strongly agree	-	25.5%	-	**38.7%**
	Agree		38.7%		**35.5%**
	Neither agree nor disagree		21.7%		16.1%
	Disagree		11.3%		3.2%
	Strongly disagree		2.8%		6.5%

Percentages were rounded.

Table S5: Responses to questions relevant to weighting (based on participant free-text comments, questions changed from round to round)

Question	Responses	Professional round 1 No. (%)	Professional round 2 No. (%)	Professional round 3 No. (%)	Public round 1 No. (%)	Public round 2 No. (%)	Public round 3 No. (%)
Please describe what type of measures you would use to measure multimorbidity*	Weighted morbidity measure<br>A simple count of conditions<br>Other	91 (53.2%)<br>69 (40.4%)<br>11 (6.4%)	-	-	-	-	-
Please choose what type of measures you would use to understand the impact of multimorbidity on outcomes	Weighted morbidity measure<br>A simple count of conditions<br>Both<br>Other	92 (61.3%)<br>38 (25.3%)<br>7 (4.7%)<br>13 (8.7%)	-	-	-	-	-
What weighting methods would you use in a multimorbidity measure?[a]*	Use existing weighted indices<br>Empirically derive weights based on the individual impact of diseases on an outcome (e.g. use regression models to calculate weights)<br>Set rules based on level of severity to grade each condition/disease category (e.g. if having "presence of a condition": 1 point, "treatment": additional 1 point, "functional limitation": additional 1 point)<br>Other	47 (27.3%)<br>60 (34.9%)<br>47 (27.3%)<br>18 (10.5%)	20 (21.7%)<br>43 (46.7%)<br>24 (26.1%)<br>5 (5.4%)	-	-	-	-
Please rate the degree to which the outcomes listed are important to weight against[a]							
Death	Not at all important<br>Slightly important<br>Important<br>Sufficiently important<br>Very important	2 (1.4%)<br>2 (1.4%)<br>6 (4.3%)<br>**12 (8.6%)**<br>**117 (84.2%)**	-	-	0<br>2 (8.3%)<br>2 (8.3%)<br>4 (16.7%)<br>16 66.7%)	0<br>0<br>1 (3.2%)<br>**3 (9.7%)**<br>**27 (87.1%)**	-
Healthcare use	Not at all important<br>Slightly important<br>Important<br>Sufficiently important<br>Very important	1 (0.7%)<br>5 (3.5%)<br>17 (12.1%)<br>**31 (22.0%)**<br>**87 (61.7%)**	-	-	0<br>0<br>7 (29.2%)<br>4 (16.7%)<br>13 54.2%)	0<br>0<br>3 (9.7%)<br>**11 (35.5%)**<br>**17 (54.8%)**	-
Health-related quality of life	Not at all important<br>Slightly important<br>Important<br>Sufficiently important<br>Very important	1 (0.7%)<br>3 (2.1%)<br>7 (4.9%)<br>**17 (11.9%)**<br>**115 (80.4%)**	-	-	0<br>3 (12.5%)<br>3 (12.5%)<br>7 (29.2%)<br>11 45.8%)	1 (3.2%)<br>0<br>2 (6.5%)<br>**8 (25.8%)**<br>**20 (64.5%)**	-

Question	Responses	Professional round 1 No. (%)	Professional round 2 No. (%)	Professional round 3 No. (%)	Public round 1 No. (%)	Public round 2 No. (%)	Public round 3 No. (%)
Physical disability	Not at all important	1 (0.7%)	-	-	0	0	-
	Slightly important	3 (2.2%)			1 (4.2%)	1 (3.3%)	
	Important	13 (9.4%)			10 41.7%)	3 (10.0%)	
	Sufficiently important	**30 (21.6%)**			6 (25.0%)	**12 (40.0%)**	
	Very important	**92 (66.2%)**			7 (29.2%)	**14 (46.7%)**	
Frailty	Not at all important	2 (1.4%)	-	-	0	0	-
	Slightly important	3 (2.2%)			5 (20.0%)	1 (3.3%)	
	Important	14 (10.1%)			10 40.0%)	6 (20.0%)	
	Sufficiently important	**42 (30.2%)**			4 (16.0%)	**11 (36.7%)**	
	Very important	**78 (56.1%)**			6 (24.0%)	**12 (40.0%)**	
Mental health	Not at all important	2 (1.4%)	-	-	0	-[b]	-
	Slightly important	6 (4.3%)			1 (4.0%)		
	Important	14 (10.1%)			8 (32.0%)		
	Sufficiently important	**36 (26.1%)**			5 (20.0%)		
	Very important	**80 (58.0%)**			11(44.0%)		
Treatment burden	Not at all important	3 (2.2%)	-	-	1 (4.2%)	1 (3.3%)	-
	Slightly important	9 (6.5%)			10 40.0%)	6 (20%)	
	Important	24 (17.3%)			2 (8.0%)	3 (10%)	
	Sufficiently important	**32 (23.0%)**			7 (28.0%)	12 (40.0%)	
	Very important	**71 (51.1%)**			5 (20.0%)	8 (26.7%)	
Healthcare costs	Not at all important	5 (3.6%)	-	-	3 (12.5%)	2 (6.7%)	-
	Slightly important	8 (5.8%)			6 (24.0%)	10 (33.3%)	
	Important	26 (18.7%)			4 (16.0%)	8 (26.7%)	
	Sufficiently important	**46 (33.1%)**			6 (24.0%)	5 (16.7%)	
	Very important	**54 (38.8%)**			6 (24.0%)	5 (16.7%)	
Self-perceived health status	Not at all important	3 (2.1%)	-	-	2 (8.0%)	2 (6.5%)	-
	Slightly important	11 (7.9%)			5 (20.0%)	6 (19.4%)	
	Important	22 (15.7%)			6 (24.0%)	7 (22.6%)	
	Sufficiently important	**40 (28.6%)**			6 (24.0%)	9 (29.0%)	
	Very important	**64 (45.7%)**			6 (24.0%)	7 (22.6%)	

Number of panelists voting "no opinion" was not added in the denominator when calculating percentages. *multiple select question; a: Non-mandatory question; b: we did not ask this question in round two because some panelists commented that conditions cannot be weighted against based on the general term "mental health"

Table S6: Professional ratings for choice of simple counts vs weighted measures depending on the purpose of a study

Question	Response	Round 2 No. of panelists (%)	Round 3 No. of panelists (%)	Round 3 summary
For the purpose of estimating the prevalence of multimorbidity	Prefer simple count<br>Prefer weighted measure<br>Either is acceptable	66 (59.5%)<br>18 (16.2%)<br>27 (24.3%)	58 (63.0%)<br>15 (16.3%)<br>19 (20.7%)	**77 (83.7%) prefer a simple count or think it equally acceptable**<br>34 (37.0%) prefer a weighted measure or think it equally acceptable
For the purpose of identifying and counting disease clusters	Prefer simple count<br>Prefer weighted measure<br>Either is acceptable	66 (68.8%)<br>15 (15.6%)<br>15 (15.6%)	59 (64.8%)<br>18 (19.8%)<br>14 (15.4%)	**73 (80.2%) prefer a simple count or think it equally acceptable**<br>32 (35.2%) prefer a weighted measure or think it equally acceptable
For the purpose of exploring trajectories of multimorbidity (e.g. trends of multimorbidity prevalence and the number of conditions an individual has had)	Prefer simple count<br>Prefer weighted measure<br>Either is acceptable	35 (32.4%)<br>27 (25.0%)<br>46 (42.6%)	34 (38.6%)<br>24 (27.3%)<br>30 (34.1%)	**64 (72.7%) prefer a simple count or think it equally acceptable**<br>54 (61.4%) prefer a weighted measure or think it equally acceptable
For the purpose of exploring/identifying predictors of multimorbidity (e.g. socio-demographic information)	Prefer simple count<br>Prefer weighted measure<br>Either is acceptable	30 (27.5%)<br>35 (32.1%)<br>44 (40.4%)	28 (30.8%)<br>32 (35.2%)<br>31 (34.1%)	59 (64.9%) prefer a simple count or think it equally acceptable<br>63 (69.3%) prefer a weighted measure or think it equally acceptable
For the purpose of assessing the severity of disease burden	Prefer simple count<br>Prefer weighted measure<br>Either is acceptable	12 (10.8%)<br>56 (50.5%)<br>43 (38.7%)	5 (5.4%)<br>66 (71.7%)<br>21 (22.8%)	26 (28.2%) prefer a simple count or think it equally acceptable<br>**87 (94.5%) prefer a weighted measure or think it equally acceptable**
For the purpose of risk adjustment or outcome prediction	Prefer simple count<br>Prefer weighted measure<br>Either is acceptable	8 (7.3%)<br>57(52.3%)<br>44 (40.4%)	8 (8.8%)<br>62 (68.1%)<br>21 (23.1%)	29 (31.9%) prefer a weighted measure or think it equally acceptable<br>**83 (91.2%) prefer a weighted measure or think it equally acceptable**

Table S7: Professional ratings for choice of simple counts vs weighted measures for outcome prediction depending on the outcome being measured

For the following outcomes of interest, please choose which type of multimorbidity measures you would prefer to use for outcome prediction [a]		Round 2 No. of panelists (%)	Round 3 No. of panelists (%)	Round 3 summary
Death	Prefer simple count<br>Prefer weighted measure<br>Either is acceptable	25 (24.5%)<br>41 (40.2%)<br>36 (35.3%)	10 (10.6%)<br>54 (57.4%)<br>30 (31.9%)	40 (42.5%) prefer a simple count or think it equally acceptable<br>**94 (89.3%) prefer a weighted measure or think it equally acceptable**
Healthcare use	Prefer simple count<br>Prefer weighted measure<br>Either is acceptable	28 (26.2%)<br>39 (36.4%)<br>40 (37.4%)	15 (16.1%)<br>41 (44.1%)<br>37 (39.8%)	52 (55.1%) prefer a simple count or think it equally acceptable<br>**78 (83.9%) prefer a weighted measure or think it equally acceptable**
Health-related quality of life	Prefer simple count<br>Prefer weighted measure<br>Either is acceptable	15 (14.2%)<br>49 (46.2%)<br>42 (39.6%)	5 (5.3%)<br>54 (57.4%)<br>35 (37.2%)	40 (42.5%) prefer a simple count or think it equally acceptable<br>**89 (94.6%) prefer a weighted measure or think it equally acceptable**
Physical disability	Prefer simple count<br>Prefer weighted measure<br>Either is acceptable	18 (16.8%)<br>49 (45.8%)<br>40 (37.4%)	8 (8.5%)<br>54 (57.4%)<br>32 (34.0%)	40 (42.5%) prefer a simple count or think it equally acceptable<br>**86 (91.4%) prefer a weighted measure or think it equally acceptable**
Frailty	Prefer simple count<br>Prefer weighted measure<br>Either is acceptable	17 (15.9%)<br>47 (43.9%)<br>43 (23.4%)	9 (9.6%)<br>52 (55.3%)<br>33 (35.1%)	42 (44.7%) prefer a simple count or think it equally acceptable<br>**85 (90.4%) prefer a weighted measure or think it equally acceptable**
Mental health	Prefer simple count<br>Prefer weighted measure<br>Either is acceptable	25 (40.2%)<br>43 (56.2%)<br>39 (36.4%)	14 (15.2%)<br>42 (45.7%)<br>36 (39.1%)	50 (54.3%) prefer a simple count or think it equally acceptable<br>78 **(84.8%) prefer a weighted measure or think it equally acceptable**
Treatment burden	Prefer simple count<br>Prefer weighted measure<br>Either is acceptable	19 (17.8%)<br>47 (43.9%)<br>41 (38.3%)	12 (12.8%)<br>53 (56.4%)<br>29 (30.9%)	41 (43.7%) prefer a simple count or think it equally acceptable<br>**82 (87.3%) prefer a weighted measure or think it equally acceptable**
Healthcare costs	Prefer simple count<br>Prefer weighted measure<br>Either is acceptable	20 (18.9%)<br>40 (37.7%)<br>46 (43.4%)	14 (15.2%)<br>41 (44.6%)<br>37 (40.2%)	51 (55.4%) prefer a simple count or think it equally acceptable<br>**78 (84.8%) prefer a weighted measure or think it equally acceptable**
Self-perceived health status	Prefer simple count<br>Prefer weighted measure<br>Either is acceptable	24 (23.1%)<br>41 (39.4%)<br>39 (37.5%)	15 (16.1%)<br>40 (43.0%)<br>38 (40.9%)	53 (57.0%) prefer a simple count or think it equally acceptable<br>**78 (83.9%) prefer a weighted measure or think it equally acceptable**

Table S8: Professional scores for 'always include'

Condition	Round 1 N=150 %	Round 2 N=112 %	Round 3 N=97 %	Professional panel consensus
Heart failure[a]	90.0	-	-	Always include
Chronic liver disease[a]	88.5	-	-	Always include
Diabetes[a]	87.3	-	-	Always include
Parkinson's disease[a]	86.6	-	-	Always include
End-stage kidney disease[a]	86.4	-	-	Always include
Chronic obstructive pulmonary disease[a]	85.9	-	-	Always include
Coronary artery disease[a]	82.7	-	-	Always include
Dementia[a]	82.6	-	-	Always include
Inflammatory bowel disease[a]	82.6	-	-	Always include
Multiple sclerosis[a]	80.7	-	-	Always include
Stroke[a]	80.0	-	-	Always include
Connective tissue disease[a]	79.7	-	-	Always include
Chronic kidney disease[a]	79.3	-	-	Always include
HIV/AIDS[a]	78.5	-	-	Always include
Metastatic cancers[a]	77.4	-	-	Always include
Haematological cancers[a]	77.2	-	-	Always include
Solid organ cancers[a]	76.5	-	-	Always include
Paralysis[a]	76.0	-	-	Always include
Cystic fibrosis[a]	75.8	-	-	Always include
Schizophrenia[a]	75.2	-	-	Always include
Epilepsy[a]	73.0	-	-	Always include
Peripheral artery disease[a]	71.1	-	-	Always include
Asthma[a]	70.7	-	-	Always include
Depression	69.6	67.0	-	
Heart valve disorders	57.7	53.6	-	
Bipolar disorder	61.9	61.8	-	
Melanoma	65.8	54.5	-	
Addison's disease[b]	-	48.1	34.5	
Bronchiectasis[b]	-	36.9	31.1	
Osteoarthritis	59.7	51.4	-	
Pancreatic disease	66.0	38.5	-	
Arrhythmia	58.4	46.4	-	
Thyroid problems	59.7	40.0	-	
Venous thrombotic disease	45.9	43.5	-	
Drug/alcohol misuse	51.4	51.8	-	
Anaemia	43.9	44.0	-	
Chronic Lyme disease[b]	-	29.8	25.3	
Transient ischaemic attack	49.3	48.2	-	
Hypertension	56.7	43.8	-	
Anxiety	52.0	47.3	-	
Treated cancer that requires surveillance[c]	-	-	35.9	
Eating disorders	45.0	44.5	-	
Vision impairment or blindness that cannot be corrected with glasses	52.7	46.4	-	
Musculoskeletal problems due to injury	44.5	41.1	36.1	
Tuberculosis	61.5	46.8	-	
Gout	45.3	38.5	-	
Endometriosis	42.4	35.5	-	
Chronic primary pain[d]	48.3	-	33.0	
Peptic ulcer	48.6	36.9	-	
Hearing impairment or deafness that cannot be corrected with hearing aids	48.0	39.6	-	
Post-acute COVID-19[b]	-	27.7	24.5	
Post-traumatic stress disorder	38.0	42.2	-	

Psoriasis	43.2	31.2	-	
Benign cerebral tumours[c]	-	-	28.9	
Peripheral neuropathy	52.4	36.1	-	
Hypertension (untreated)	54.7	39.6	-	
Sleep apnoea	45.5	29.9	-	
Congenital disease and chromosomal abnormalities	46.2	35.8	-	
Chronic low back pain[d]	-	-	30.9	
Chronic urinary tract infection	38.6	30.0	-	
Aneurysm[c]	-	-	22.7	
Meniere’s disease	34.9	26.9	-	
Migraine or other regular headache	37.8	28.7	-	
Osteoporosis	52.0	41.4	-	
Autism	36.2	29.0	-	
Obsessive compulsive disorder	38.6	32.7	-	
Dissociative or personality disorders	41.0	40.7	-	
Glaucoma	44.2	36.0	-	
Urinary incontinence	38.8	31.5	-	
Irritable bowel syndrome	38.8	29.4	-	
Polycystic ovary syndrome	36.6	29.8	-	
Chronic insomnia	35.0	30.8	-	
Obesity	48.3	35.1	-	
Dyslipidaemia (treated)	39.6	33.6	-	
Uterine (womb) problems	31.0	21.6	-	
Cataract	31.8	26.1	-	
Gall bladder problems	35.9	26.4	-	
Prostatic hypertrophy	29.5	27.2	-	
Attention deficit hyperactivity disorder	27.0	25.9	-	
Somatoform disorders	30.7	25.5	-	
Gastroesophageal reflux	32.0	27.3	-	
Chronic urticaria	29.1	19.2	-	
Malnutrition	36.2	28.3	-	
High cholesterol (untreated)	44.9	25.5	-	
Learning disability	28.1	25.2	-	
Kidney or bladder stones	28.6	22.4	-	
Physical disability	38.9	27.9	-	
Eczema	25.9	19.3	-	
Non-melanoma skin cancer	30.3	23.1	-	
Chronic sinusitis	27.4	17.3	-	
Chronic/allergic rhinitis	30.1	17.4	-	
Diverticulosis	38.7	22.2	-	
Chronic gum disease	16.8	19.8	-	
Nicotine dependence	20.0	22.2	-	
Sexual dysfunction	14.0	16.2	-	
Edentulism	20.1	18.7	-	
Smoking	29.3	27.9	-	
Social deprivation and poverty	24.3	17.9	-	
Infertility	10.6	14.0	-	
Post-sepsis syndrome	14.3	15.7	-	
Treatment burden	16.1	14.5	-	
Dizziness	10.3	10.8	-	
Side effects of medications	12.0	11.9	-	
Chronic cough	8.3	10.7	-	
Benign cancers	18.6	9.3	-	
Sedentary lifestyle	14.4	9.9	-	
Treated cancer not requiring surveillance[c]	-	-	5.4	

a. Reached ‘always include’ consensus after round 1
b. Proposed as additional conditions by participants after round 1
c. Proposed as additional conditions by participants after round 2
d. Chronic pain asked about in rounds 1, split into chronic primary pain and chronic low back pain by participants after round 2

Table S9: Public scores for 'always include'

Condition	Round 1 N=150 %	Round 2 N=112 %	Round 3 N=97 %	Public panel consensus
End-stage kidney disease	68.0	90.3	-	Always include
Heart failure	68.0	83.9	-	Always include
Dementia	60.0	83.3	-	Always include
Chronic liver disease	64.0	80.6	-	Always include
Stroke	60.0	80.6	-	Always include
Chronic kidney disease	64.0	80.6	-	Always include
Parkinson's disease	56.0	77.4	-	Always include
Multiple sclerosis	60.0	77.4	-	Always include
Coronary artery disease	64.0	74.2	-	Always include
Cystic fibrosis	62.5	74.2	-	Always include
Diabetes	64.0	71.0	-	Always include
HIV/AIDS	37.5	71.0	-	Always include
Epilepsy	44.0	71.0	-	Always include
Addison's disease	-	40.0	70.8	Always include
Metastatic cancers	70.8	80.0	-	Always include
Haematological cancers	70.8	77.4	-	Always include
Solid organ cancers	70.8	80.0	-	Always include
Inflammatory bowel disease	60.0	58.1	-	
Heart valve disorders	36.0	61.3	-	
Melanoma	58.3	67.7	-	
Chronic obstructive pulmonary disease	60.0	67.7	-	
Peripheral artery disease	24.0	35.5	-	
Venous thrombotic disease	48.0	64.5	-	
Transient ischaemic attack	32.0	64.5	-	
Aneurysm	-	67.7	-	
Pancreatic disease	48.0	53.3	-	
Anaemia	24.0	43.3	-	
Peripheral neuropathy	44.0	26.7	-	
Schizophrenia	56.5	67.7	-	
Bipolar disorder	52.2	51.6	-	
Connective tissue disease	54.2	66.7	-	
Paralysis	56.0	63.3	-	
Post-acute COVID-19	-	44.8	60.0	
Tuberculosis	54.2	54.8	-	
Congenital disease	44.0	53.3	-	
Prostatic hypertrophy	25.0	23.3	-	
Endometriosis	36.4	50.0	-	
Bronchiectasis	-	45.2	48.0	
Osteoarthritis	41.7	41.9	-	
Thyroid problems	37.5	35.5	-	
Autism	22.7	35.5	-	
Chronic urinary tract infection	20.0	40.0	-	
Polycystic ovary syndrome	40.9	37.9	-	
Arrhythmia	28.0	39.3	-	
Peptic ulcer	29.2	35.5	-	
Learning disability	25.0	38.7	-	
Asthma	32.0	58.1	-	
Osteoporosis	33.3	45.2	-	
Uterine (womb) problems	22.7	35.5	-	
Treated cancer that requires surveillance	-	-	36.0	
Chronic primary pain	24.0	-	20.0	
Malnutrition	14.3	36.7	-	
Non-melanoma skin cancer	30.4	36.7	-	
Chronic Lyme disease	-	38.7	45.8	

Depression	37.5	25.8	-	
Irritable bowel syndrome	20.0	26.7	-	
Kidney or bladder stones	20.0	26.7	-	
Benign cerebral tumours	-		52.0	
Diverticulosis	25.0	24.1	-	
Drug/alcohol misuse	20.8	35.5	-	
Eating disorders	34.8	45.2	-	
Vision impairment or blindness that cannot be corrected with glasses	36.0	41.9	-	
Hearing impairment or deafness that cannot be corrected with hearing aids	25.0	38.7	-	
Post-traumatic stress disorder	30.4	25.8	-	
Dyslipidaemia (treated)	24.0	29.0	-	
Physical disability	29.2	41.9	-	
Hypertension (untreated)	29.2	35.5	-	
Meniere’s disease	29.2	25.8	-	
Dissociative or personality disorders	42.9	41.9	-	
Musculoskeletal problems due to injury	25.0	16.1	45.8	
Glaucoma	39.1	33.3	-	
Gall bladder problems	29.2	36.7	-	
Sleep apnoea	37.5	19.4	-	
Obsessive compulsive disorder	14.3	22.6	-	
Urinary incontinence	20.8	22.6	-	
Chronic/allergic rhinitis	16.7	16.1	-	
Gout	25.0	19.4	-	
Psoriasis	16.7	16.1	-	
Obesity	20.8	16.1	-	
Attention deficit hyperactivity disorder	19.0	12.9	-	
Somatoform disorders	5.0	17.2	-	
Hypertension (treated)	29.2	16.7	-	
Anxiety	25.0	16.7	-	
Benign cancers	17.4	17.2	-	
Migraine or other regular headache	20.8	12.9	-	
Gastroesophageal reflux	24.0	12.9	-	
Eczema	20.8	6.5	-	
Post-sepsis syndrome	23.8	28.6	-	
Chronic low back pain	-	-	24.0	
Cataract	20.8	16.1	-	
Treatment burden	12.5	20.0	-	
Side effects of medications	16.7	23.3	-	
Social deprivation and poverty	17.4	23.3	-	
Chronic sinusitis	16.0	9.7	-	
Nicotine dependence	13.6	22.6	-	
High cholesterol (untreated)	29.2	12.9	-	
Smoking	17.4	16.1	-	
Chronic insomnia	18.2	13.3	-	
Chronic urticaria	13.0	6.9	-	
Chronic gum disease	21.7	9.7	-	
Chronic cough	12.5	3.2	-	
Sedentary lifestyle	0.0	9.7	-	
Sexual dysfunction	13.6	13.8	-	
Edentulism	8.3	6.5	-	
Dizziness	17.4	3.2	-	
Infertility	22.7	17.2	-	
Treated cancer not requiring surveillance	-	-	8.0	

Table S10: Professional scores for 'usually include' (the sum of 'always' and 'usually')

Condition	Round 1 N=150 %	Round 2 N=112 %	Round 3 N=97 %	Professional panel consensus
Heart failure	98.7	-	-	Always include
Chronic liver disease	98.6	-	-	Always include
Diabetes	98.7	-	-	Always include
Parkinson's disease	98.0	-	-	Always include
End-stage kidney disease	94.6	-	-	Always include
Chronic obstructive pulmonary disease	98.7	-	-	Always include
Coronary artery disease	97.3	-	-	Always include
Dementia	96.6	-	-	Always include
Inflammatory bowel disease	94.0	-	-	Always include
Multiple sclerosis	94.0	-	-	Always include
Stroke	97.3	-	-	Always include
Connective tissue disease	97.3	-	-	Always include
Chronic kidney disease	97.3	-	-	Always include
HIV/AIDS	94.0	-	-	Always include
Metastatic cancers	91.1	-	-	Always include
Haematological cancers	94.0	-	-	Always include
Solid organ cancers	94.6	-	-	Always include
Paralysis	88.4	-	-	Always include
Cystic fibrosis	90.6	-	-	Always include
Schizophrenia	94.0	-	-	Always include
Epilepsy	95.3	-	-	Always include
Peripheral artery disease	92.6	-	-	Always include
Asthma	92.7	-	-	Always include
Depression	93.9	92.9	-	Usually include
Heart valve disorder	85.2	92.0	-	Usually include
Bipolar disorder	90.5	90.0	-	Usually include
Melanoma	87.7	88.2	-	Usually include
Addison's disease	-	83.7	86.9	Usually include
Bronchiectasis	-	78.6	86.7	Usually include
Osteoarthritis	87.9	84.7	-	Usually include
Pancreatic disease	88.9	84.4	-	Usually include
Arrhythmia	85.9	83.9	-	Usually include
Thyroid problems	83.9	82.7	-	Usually include
Venous thrombotic disease	77.4	82.4	-	Usually include
Drug/alcohol misuse	82.4	81.8	-	Usually include
Anaemia	75.0	81.7	-	Usually include
Chronic Lyme disease	-	61.5	81.3	Usually include
Transient ischaemic attack	76.7	80.4	-	Usually include
Hypertension (treated)	78.7	80.4	-	Usually include
Anxiety	86.5	80.0	-	Usually include
Treated cancer that requires surveillance	-	-	79.3	Usually include
Eating disorders	74.3	79.1	-	Usually include
Vision impairment or blindness that cannot be corrected with glasses	80.1	78.6	-	Usually include
Musculoskeletal problems due to injury	80.1	78.6	78.4	Usually include
Tuberculosis	82.4	76.6	-	Usually include
Gout	79.1	76.1	-	Usually include
Endometriosis	73.6	75.7	-	Usually include
Chronic primary pain	78.5	-	75.3	Usually include
Peptic ulcer	79.7	73.9	-	Usually include
Hearing impairment or deafness that cannot be corrected with hearing aids	70.9	73.9	-	Usually include
Post-acute COVID-19	-	70.3	73.4	Usually include
Post-traumatic stress disorder	77.5	73.4	-	Usually include

Psoriasis	77.0	73.4	-	Usually include
Benign cerebral tumours	-	-	73.3	Usually include
Peripheral neuropathy	83.4	73.1	-	Usually include
Hypertension (untreated)	73.6	73.0	-	Usually include
Sleep apnoea	77.2	72.9	-	Usually include
Congenital disease	79.7	72.6	-	Usually include
Chronic low back pain	-	-	72.2	Usually include
Chronic urinary tract infection	73.8	71.8	-	Usually include
Aneurysm	-	-	71.6	Usually include
Meniere’s disease	63.0	71.3	-	Usually include
Migraine or other regular headache	68.9	71.3	-	Usually include
Osteoporosis	77.3	70.3	-	Usually include
Autism	63.0	70.1	-	Usually include
Obsessive compulsive disorder	63.6	70.1	-	Usually include
Dissociative or personality disorders	69.8	69.4	-	
Glaucoma	72.8	69.4	-	
Urinary incontinence	67.3	66.7	-	
Irritable bowel syndrome	63.9	66.1	-	
Polycystic ovary syndrome	66.9	65.4	-	
Chronic insomnia	69.3	64.5	-	
Obesity	69.1	64.0	-	
Dyslipidaemia (treated)	63.1	63.6	-	
Uterine (womb) problems	71.0	63.1	-	
Cataract	62.2	63.1	-	
Gall bladder problems	67.6	62.7	-	
Prostatic hypertrophy	55.0	62.1	-	
Attention deficit hyperactivity disorder	56.9	60.2	-	
Somatoform disorders	55.7	59.4	-	
Gastroesophageal reflux	60.5	59.1	-	
Chronic urticaria	59.6	58.7	-	
Malnutrition	64.5	57.5	-	
High cholesterol (untreated)	63.9	57.3	-	
Learning disability	53.3	55.1	-	
Kidney or bladder stones	57.1	55.1	-	
Physical disability	60.4	55.0	-	
Eczema	60.5	54.1	-	
Non-melanoma skin cancer	54.5	53.7	-	
Chronic sinusitis	54.1	53.6	-	
Chronic/allergic rhinitis	58.2	53.2	-	
Diverticulosis	69.7	50.0	-	
Chronic gum disease	46.2	49.1	-	
Nicotine dependence	42.9	48.1	-	
Sexual dysfunction	42.0	46.7	-	
Edentulism	38.8	41.1	-	
Smoking	45.6	37.8	-	
Social deprivation and poverty	41.0	36.6	-	
Infertility	33.3	33.6	-	
Post-sepsis syndrome	42.1	32.4	-	
Treatment burden	27.3	31.8	-	
Dizziness	33.6	30.6	-	
Side effects of medications	28.9	29.4	-	
Chronic cough	29.0	27.7	-	
Benign cancers	35.9	26.2	-	
Sedentary lifestyle	28.8	18.0	-	
Treated cancer not requiring surveillance	-	-	21.5	

Table S11: Public scores for 'usually include' (the sum of 'always' and 'usually')

Condition	Round 1 N=25 %	Round 2 N=31 %	Round 3 N=25 %	Public panel consensus
End-stage kidney disease	96.0	100.0	-	Always include
Heart failure	100.0	96.8	-	Always include
Dementia	92.0	96.7	-	Always include
Chronic liver disease	100.0	100.0	-	Always include
Stroke	92.0	96.8	-	Always include
Chronic kidney disease	100.0	100.0	-	Always include
Parkinson's disease	92.0	96.8	-	Always include
Multiple sclerosis	100.0	100.0	-	Always include
Coronary artery disease	96.0	96.8	-	Always include
Cystic fibrosis	100.0	96.8	-	Always include
Diabetes	92.0	100.0	-	Always include
HIV/AIDS	91.7	90.3	-	Always include
Epilepsy	92.0	100.0	-	Always include
Addison's disease	-	88.0	95.8	Always include
Metastatic cancers	91.7	96.7	-	Always include
Haematological cancers	91.7	100.0	-	Always include
Solid organ cancers	91.7	100.0	-	Always include
Inflammatory bowel disease	100.0	100.0	-	Usually include
Heart valve disorders	76.0	100.0	-	Usually include
Melanoma	91.7	100.0	-	Usually include
Chronic obstructive pulmonary disease	100.0	96.8	-	Usually include
Peripheral artery disease	88.0	96.8	-	Usually include
Venous thrombotic disease	80.0	96.8	-	Usually include
Transient ischaemic attack	88.0	96.8	-	Usually include
Aneurysm	-	96.8	-	Usually include
Pancreatic disease	96.0	96.7	-	Usually include
Anaemia	60.0	96.7	-	Usually include
Peripheral neuropathy	76.0	96.7	-	Usually include
Schizophrenia	91.3	93.5	-	Usually include
Bipolar disorder	95.7	93.5	-	Usually include
Connective tissue disease	83.3	93.3	-	Usually include
Paralysis	84.0	93.3	-	Usually include
Post-acute COVID-19	-	93.1	92.0	Usually include
Tuberculosis	91.7	90.3	-	Usually include
Congenital disease	88.0	90.0	-	Usually include
Prostatic hypertrophy	58.3	90.0	-	Usually include
Endometriosis	72.7	89.3	-	Usually include
Bronchiectasis	-	87.1	88.0	Usually include
Osteoarthritis	79.2	87.1	-	Usually include
Thyroid problems	75.0	87.1	-	Usually include
Autism	63.6	87.1	-	Usually include
Chronic urinary tract infection	60.0	86.7	-	Usually include
Polycystic ovary syndrome	72.7	86.2	-	Usually include
Arrhythmia	68.0	85.7	-	Usually include
Peptic ulcer	66.7	83.9	-	Usually include
Learning disability	50.0	83.9	-	Usually include
Asthma	96.0	80.6	-	Usually include
Osteoporosis	70.8	80.6	-	Usually include
Uterine (womb) problems	63.6	80.6	-	Usually include
Treated cancer that requires surveillance	-	-	80.0	Usually include
Chronic primary pain	76.0	-	80.0	Usually include
Malnutrition	57.1	80.0	-	Usually include
Non-melanoma skin cancer	69.6	80.0	-	Usually include
Chronic Lyme disease	-	83.9	79.2	Usually include

Depression	87.5	77.4	-	Usually include
Irritable bowel syndrome	68.0	76.7	-	Usually include
Kidney or bladder stones	52.0	76.7	-	Usually include
Benign cerebral tumours	58.3	-	76.0	Usually include
Diverticulosis	62.5	75.9	-	Usually include
Drug/alcohol misuse	78.3	74.2	-	Usually include
Eating disorders	72.0	74.2	-	Usually include
Vision impairment or blindness that cannot be corrected with glasses	58.3	74.2	-	Usually include
Hearing impairment or deafness that cannot be corrected with hearing aids	65.2	74.2	-	Usually include
Post-traumatic stress disorder	64.0	74.2	-	Usually include
Dyslipidaemia (treated)	70.8	74.2	-	Usually include
Physical disability	79.2	74.2	-	Usually include
Hypertension (untreated)	75.0	71.0	-	Usually include
Meniere’s disease	81.0	71.0	-	Usually include
Dissociative or personality disorders	70.8	71.0	-	Usually include
Musculoskeletal problems due to injury	69.6	67.7	70.8	Usually include
Glaucoma	58.3	70.0	-	Usually include
Gall bladder problems	62.5	70.0	-	Usually include
Sleep apnoea	47.6	67.7	-	
Obsessive compulsive disorder	41.7	67.7	-	
Urinary incontinence	50.0	67.7	-	
Chronic/allergic rhinitis	41.7	67.7	-	
Gout	54.2	64.5	-	
Psoriasis	58.3	64.5	-	
Obesity	47.6	61.3	-	
Attention deficit hyperactivity disorder	45.0	61.3	-	
Somatoform disorders	83.3	58.6	-	
Hypertension (treated)	62.5	56.7	-	
Anxiety	52.2	56.7	-	
Benign cancers	58.3	55.2	-	
Migraine or other regular headache	56.0	54.8	-	
Gastroesophageal reflux	45.8	54.8	-	
Eczema	66.7	54.8	-	
Post-sepsis syndrome	-	53.6	-	
Chronic low back pain	41.7	-	52.0	
Cataract	29.2	51.6	-	
Treatment burden	41.7	50.0	-	
Side effects of medications	39.1	50.0	-	
Social deprivation and poverty	44.0	46.7	-	
Chronic sinusitis	40.9	45.2	-	
Nicotine dependence	66.7	45.2	-	
High cholesterol (untreated)	34.8	41.9	-	
Smoking	54.5	41.9	-	
Chronic insomnia	43.5	40.0	-	
Chronic urticaria	34.8	37.9	-	
Chronic gum disease	54.2	35.5	-	
Chronic cough	13.0	35.5	-	
Sedentary lifestyle	45.5	35.5	-	
Sexual dysfunction	25.0	34.5	-	
Edentulism	47.8	32.3	-	
Dizziness	45.5	32.3	-	
Infertility	-	27.6	-	
Treated cancer not requiring surveillance		-	24.0	

Table S12: Conditions *not* recommended to 'always include' or 'usually include' by *both* panels

Condition	Professional panel (%)	Public panel (%)	Both panels (difficulty parameter, logit)	Final consensus
**Consensus to usually include in one panel (n=22)**				
Hypertension (treated)	80.4 (R2)	56.7 (R2)	0.3 (R2)	Usually include*
Anxiety	80.0 (R2)	56.7 (R2)	0.4 (R2)	Usually include*
Gout	76.1 (R2)	64.5 (R2)	0.4 (R2)	Usually include*
Psoriasis	73.4 (R2)	64.5 (R2)	0.6 (R2)	No consensus
Sleep apnoea	72.9 (R2)	67.7 (R2)	0.6 (R2)	No consensus
Obsessive compulsive disorder	70.1 (R2)	67.7 (R2)	0.7 (R2)	No consensus
Prostatic hypertrophy	62.1 (R2)	90.0 (R2)	0.7 (R2)	No consensus
Polycystic ovary syndrome	65.4 (R2)	86.2 (R2)	0.7 (R2)	No consensus
Dissociative or personality disorders	69.4 (R2)	71.0 (R2)	0.7 (R2)	No consensus
Glaucoma	69.4 (R2)	70.0 (R2)	0.7 (R2)	No consensus
Chronic low back pain	72.2 (R3)	52.0 (R3)	0.8 (R3)	No consensus
Migraine	71.3 (R2)	54.8 (R2)	0.8 (R2)	No consensus
Uterine (womb) problems	63.1 (R2)	80.0 (R2)	0.8 (R2)	No consensus
Irritable bowel syndrome	66.1 (R2)	76.7 (R2)	0.8 (R2)	No consensus
Gall bladder problems	62.7 (R2)	70.0 (R2)	1.0 (R2)	No consensus
Malnutrition	57.5 (R2)	80.0 (R2)	1.1 (R2)	No consensus
Learning disability	55.1 (R2)	83.9 (R2)	1.2 (R2)	No consensus
Kidney or bladder stones	55.1 (R2)	76.7 (R2)	1.2 (R2)	No consensus
High cholesterol (untreated)	57.3 (R2)	74.2 (R2)	1.2 (R2)	No consensus
Non-melanoma skin cancer	53.7 (R2)	80.6 (R2)	1.3 (R2)	No consensus
Physical disability	55.0 (R2)	74.2 (R2)	1.3 (R2)	No consensus
Diverticulosis	50.0 (R2)	75.9 (R2)	1.5 (R2)	No consensus
**No consensus in either panel (n=27)**				
Urinary incontinence	66.7 (R2)	67.7 (R2)	0.9 (R2)	No consensus
Obesity	64.0 (R2)	61.3 (R2)	1.0 (R2)	No consensus
Cataract	63.1 (R2)	51.6 (R2)	1.2 (R2)	No consensus
Attention deficit hyperactivity disorder	60.2 (R2)	61.3 (R2)	1.2 (R2)	No consensus
Gastroesophageal reflux	59.1 (R2)	54.8 (R2)	1.3 (R2)	No consensus
Chronic insomnia	64.5 (R2)	40.0 (R2)	1.3 (R2)	No consensus
Dyslipidaemia (treated)	63.6 (R2)	41.9 (R2)	1.3 (R2)	No consensus
Somatoform disorders	59.4 (R2)	58.6 (R2)	1.3 (R2)	No consensus
Chronic/Allergic rhinitis	53.2 (R2)	67.7 (R2)	1.4 (R2)	No consensus
Eczema	54.1 (R2)	54.8 (R2)	1.5 (R2)	No consensus
Chronic urticarial (chronic hives)	58.7 (R2)	37.9 (R2)	1.6 (R2)	No consensus
Chronic sinusitis	53.6 (R2)	45.2 (R2)	1.7 (R2)	No consensus
Chronic gum disease	49.1 (R2)	35.5 (R2)	1.9 (R2)	No consensus
Nicotine dependence	48.1 (R2)	45.2 (R2)	2.0 (R2)	No consensus
Sexual dysfunction	46.7 (R2)	34.5 (R2)	2.1 (R2)	No consensus
Edentulism	41.1 (R2)	32.3 (R2)	2.3 (R2)	No consensus
Smoking	37.8 (R2)	41.9 (R2)	2.4 (R2)	No consensus
Social deprivation and poverty	36.6 (R2)	46.7 (R2)	2.4 (R2)	No consensus
Post-sepsis syndrome	32.4 (R2)	53.6 (R2)	2.5 (R2)	No consensus
Treatment burden	31.8 (R2)	50.0 (R2)	2.5 (R2)	No consensus
Benign cancers	26.2 (R2)	55.2 (R2)	2.7 (R2)	No consensus
Infertility	33.6 (R2)	27.6 (R2)	2.7 (R2)	No consensus
Side effects of medications	29.4 (R2)	50.0 (R2)	2.8 (R2)	No consensus
Chronic cough	27.7 (R2)	35.5 (R2)	2.9 (R2)	No consensus
Dizziness	30.6 (R2)	32.3 (R2)	2.9 (R2)	No consensus
Treated cancer not requiring surveillance	21.5 (R3)	24.0 (R3)	3.4 (R3)	No consensus
Sedentary lifestyle	18.0 (R2)	35.5 (R2)	3.4 (R2)	No consensus

* Consensus to 'usually include' in one panel *and* difficulty parameter across both panels ≤0.5

Table S13: Differential item functioning (used to examine which condition-choice items were more likely to be endorsed by panelists with varying interest in different populations [4])

Subgroup	Condition	Group 1 (b parameter, logit)	Group 0 (b parameter, logit)	Z-statistic	p-value
Panelists specifically interested in multimorbidity in women (group1) versus those not specifically interested (group 0)	Anaemia	-0.6	-0.2	-0.7	0.51
	Depression	-1.0	-1.8	0.9	0.35
	Anxiety	0.04	-0.1	0.3	0.78
	Dementia	-1.9	-2.8	0.9	0.35
	Eating disorder	-0.3	0.06	-0.5	0.62
	Osteoarthritis	-1.5	-0.3	-1.5	0.14
	Osteoporosis	0.04	0.7	-1.2	0.25
	Connective tissue disease	-2.6	-2.8	0.2	0.86
	Endometriosis	0.08	0.3	-0.3	0.78
	Chronic urinary tract infection	0.3	0.5	-0.4	0.71
	Polycystic ovary syndrome	0.9	0.9	-0.004	1.0
	Urinary incontinence	0.3	1.0	-1.2	0.25
	Uterus (womb) problems	1.0	1.0	0.004	1.0
Panelists specifically interested in multimorbidity in children (group1) versus those not specifically interested (group 0)	Anxiety	-0.8	0.01	-0.9	0.37
	Depression	-0.8	-1.7	1.0	0.32
	Drug/Alcohol misuse	-0.8	-0.2	-0.7	0.50
	Eating disorder	-0.2	0.02	-0.2	0.83
	Autism	-0.8	0.8	-1.8	0.07
	Learning disability	1.4	1.5	-0.08	0.94
	Attention deficit hyperactivity disorder	-0.2	1.4	**-2.0**	**0.04**
	Haematological cancers	-1.8	-1.9	0.01	1.0
	Benign brain tumour	2.9	3.2	-0.4	0.65
	Epilepsy	-1.0	-2.4	1.5	0.12
	Cystic fibrosis	-1.8	-1.3	-0.5	0.61
	Congenital diseases	-0.08	0.5	-0.7	0.46
	Asthma	-1.8	-1.6	-0.2	0.84
Panelists specifically interested in multimorbity in socially-deprived populations (group1) versus those not specifically interested (group 0)	Anxiety	-0.7	0.4	-2.1	0.04
	Depression	-1.5	-2.0	0.6	0.56
	Schizophrenia	-1.5	-2.6	1.4	0.15
	Drug/Alcohol misuse	-0.4	-0.2	-0.4	0.71
	Nicotine dependence	1.6	1.9	-0.6	0.52
	Post-traumatic stress disorder	0.3	0.4	-0.2	0.85
	Bipolar disorder	-1.2	-1.1	-0.1	0.91
	Chronic liver disease	-3.4	-3.0	0.4	0.69
	HIV/AIDS	-2.0	-2.1	0.2	0.88

Subgroup	Condition	Group 1 (b parameter, logit)	Group 0 (b parameter, logit)	Z-statistic	p-value
	Tuberculosis	0.07	0.01	0.1	0.91

p-value larger than 0.05 indicates that there is no statistical difference between group 1 and group 0. A positive value for the z-statistic indicates that group 0 is more likely to endorse the condition than group 1.

Table S14: Level of endorsement of the condition-related items

Condition	Difficulty parameter (log(odds), 95% Confidence Interval)		
	Professional panel	Public panel	Both panels combined
**System domain**			
Cardiovascular diseases	**-4.2 (-6.1 to -2.2)**	**-1.0 (-2.2 to 0.2)**	**-2.8 (-3.8 to -1.8)**
Respiratory diseases	**-3.8 (-5.8 to -1.8)**	**endorsed by all panelists**	**-3.9 (-5.9 to -2.0)**
Neurological diseases	**-3.4 (-4.9 to -2.0)**	**-1.5 (-2.9 to -0.03)**	**-2.8 (-3.8 to -1.8)**
Metabolic and endocrine	**-2.7 (-3.7 to -1.7)**	**-1.0 (-2.2 to 0.2)**	**-2.2 (-3.0 to -1.4)**
Mental health	**-2.2 (-3.1 to -1.4)**	**-0.2 (-1.1 to 0.8)**	**-1.6 (-2.2 to -0.9)**
Haematological disorders	**-2.0 (-3.0 to -1.1)**	**-1.0 (-2.2 to 0.2)**	**-1.7 (-2.5 to -1.0)**
Cancers	**-1.9 (-2.7 to 1.2)**	**-1.5 (-2.9 to -0.03)**	**-1.8 (-2.4 to -1.1)**
Musculoskeletal diseases	**-1.6 (-2.3 to 1.0)**	**-0.7 (-1.8 to 0.4)**	**-1.4 (-2.0 to -0.8)**
Digestive diseases	**-1.1 (-1.8 to -0.4)**	**-1.0 (-2.2 to 0.2)**	**-1.0 (-1.6 to -0.4)**
Chronic infections	**-1.2 (-1.9 to -0.5)**	0.3 (-0.6 to 1.1)*	**-0.7 (-1.2 to -0.2)**
Urogenital disorders	**-0.8 (-1.4 to -0.1)**	**-0.7 (-1.8 to 0.4)**	**-0.7 (-1.2 to -0.2)**
Eye diseases	**0.05 (-0.5 to 0.6)**	0.3 (-0.6 to 1.1)*	0.1 (-0.3 to 0.6)*
Ear, Nose and Throat disorders	0.3 (-0.2 to 0.8)*	**-0.2 (-1.1 to 0.8)**	0.2 (-0.2 to 0.7)*
Oral disorders	0.4 (-0.05 to 0.9)*	0.1 (-0.8 to 1.0)*	0.4 (-0.02 to 0.8)*
Skin disorders	0.5 (0.02 to 1.0)*	0.1 (-0.8 to 1.0)*	0.4 (-0.02 to 0.9)*
**Mental health and behaviour disorders**			
Dementia	**-2.4 (-3.4 to -1.5)**	**-2.2 (-4.2 to -0.2)**	**-2.3 (-3.2 to -1.5)**
Schizophrenia	**-1.7 (-2.4 to -1.0)**	**-1.5 (-2.9 to -0.03)**	**-1.6 (-2.2 to -1.0)**
Depression	**-1.5 (-2.3 to -0.7)**	**0.1 (-0.8 to 1.0)**	**-0.9 (-1.5 to -0.4)**
Bipolar disorder	**-1.0 (-1.7 to -0.3)**	**-1.5 (-2.9 to -0.03)**	**-1.1 (-1.7 to -0.5)**
Anxiety	**-0.02 (-0.6 to 0.5)**	1.2 (0.4 to 1.9)	0.4 (-0.08 to 0.8)*
Drug/Alcohol misuse	**-0.2 (-0.7 to 0.4)**	0.3 (-0.6 to 1.1)*	**-0.02 (-0.5 to 0.4)**
Eating disorder	0.1 (-0.5 to 0.6)*	0.3 (-0.6 to 1.1)*	0.2 (-0.3 to 0.6)*
Post-traumatic stress disorder	0.5 (-0.002 to 1.0)*	0.3 (-0.6 to 1.1)*	0.5 (0.05 to 0.9)*
Dissociative or personality disorder	0.7 (0.3 to 1.2)	0.4 (-0.4 to 1.3)*	0.7 (0.3 to 1.1)
Chronic insomnia	1.0 (0.6 to 1.5)	1.9 (1.1 to 2.8)	1.3 (0.9 to 1.7)
Autism	0.7 (0.2 to 1.2)	**-0.7 (-1.8 to 0.4)**	0.5 (0.03 to 0.9)*
Obsessive compulsive disorder	0.5 (0.2 to 1.2)*	0.6 (0.2 to 1.4)	0.7 (0.3 to 1.1)
Attention deficit hyperactivity disorder	1.3 (0.8 to 1.7)	1.4 (0.7 to 2.2)	1.2 (0.8 to 1.6)
Somatoform disorders	1.3 (0.9 to 1.8)	1.0 (0.2 to 1.7)	1.3 (0.9 to 1.7)
Learning disability	1.5 (1.1 to 2.0)	**-0.4 (-1.4 to 0.6)**	1.2 (0.8 to 1.5)
Nicotine dependence	1.9 (1.5 to 2.3)	1.8 (1.0 to 2.5)	1.9 (1.5 to 2.3)
**Cardiovascular diseases**			
Heart failure	**-3.4 (-4.9 to -2.0)**	**-2.2 (-4.2 to -0.2)**	**-3.1 (-4.3 to -2.0)**
Coronary heart diseases	**-2.7 (-3.7 to -1.7)**	**-2.2 (-4.2 to -0.2)**	**-2.6 (-3.5 to -1.6)**
Stroke	**-2.7 (-3.7 to -1.7)**	**-2.2 (-4.2 to -0.2)**	**-2.6 (-3.5 to -1.6)**
Peripheral artery diseases	**-1.5 (-2.2 to -0.9)**	**-2.2 (-4.2 to -0.2)**	**-1.6 (-2.2 to -0.9)**
Arrhythmia	**-0.4 (-1.0 to 0.2)**	**-0.6 (1.7 to 0.5)**	**-0.4 (-0.9 to 0.1)**
Heart valve disorders	**-0.3 (-0.8 to 0.2)**	**endorsed by all panelists**	**-1.6 (-2.3 to -0.9)**
Venous thrombotic disease	**-0.3 (-0.8 to -0.3)**	**-2.2 (-4.2 to -0.2)**	**-0.5 (-1.0 to -0.003)**
Hypertension (treated)	**-0.1 (-0.6 to 0.4)**	1.2 (0.4 to 2.0)	0.3 (-0.1 to 0.7)*

Hypertension (untreated)	0.4 (-0.05 to 0.9)*	0.4 (-0.4 to 1.3)*	0.4 (-0.1 to 0.9)*
Aneurysm	0.6 (0.05 to 1.1)	**-2.2 (-4.2 to -0.2)**	0.5 (0.1 to 0.9)*
Dyslipidaemia (treated)	1.0 (0.6 to 1.5)	1.9 (1.1 to 2.7)	1.3 (0.9 to 1.7)
High cholesterol (untreated)	1.4 (0.9 to 1.8)	0.4 (-0.4 to 1.3)*	1.2 (0.8 to 1.5)
**Disorders of the blood**			
Anaemia	**-0.2 (-0.7 to 0.4)**	**-2.2 (-4.2 to -0.2)**	**-0.4 (-0.9 to 0.08)**
**Cancers**			
Solid organ cancers	**-1.9 (-2.6 to -1.1)**	**endorsed by all panelists**	**-2.0 (-2.7 to -1.3)**
Haematological cancers	**-1.7 (-2.5 to -1.0)**	**endorsed by all panelists**	**-1.9 (-2.6 to -1.2)**
Metastatic cancers	**-1.3 (-1.9 to -0.6)**	**-2.2 (-4.2 to -0.2)**	**-1.3 (-1.9 to -0.7)**
Melanoma	**-0.8 (-1.5 to -0.2)**	**endorsed by all panelists**	**-1.1 (-1.7 to -0.5)**
Treated cancers requiring surveillance	**-0.04 (-0.6 to 0.5)**	0.03 (-1.0 to 1.1)*	0.01 (-0.5 to 0.5)*
Benign cerebral tumours	0.3 (-0.2 to 0.9)*	0.3 (-0.7 to 1.3)*	0.4 (-0.1 to 0.8)*
Non-melanoma skin cancers	1.6 (1.2 to 2.0)	**-0.1 (-1.0 to 0.9)**	1.3 (0.9 to 1.7)
Benign cancers	3.2 (2.7 to 3.7)	1.3 (0.5 to 2.1)	2.7 (2.3 to 3.1)
Treated cancer not requiring surveillance	3.4 (2.9 to 4.0)	3.1 (2.1 to 4.1)	3.4 (2.9 to 3.9)
**Neurological diseases**			
Parkinson’s disease	**-3.0 (-4.2 to -1.8)**	**-2.2 (-4.2 to -0.2)**	**-2.8 (-3.8 to -1.8)**
Epilepsy	**-2.1 (-2.9 to -1.3)**	**endorsed by all panelists**	**-2.2 (-3.0 to -1.4)**
Multiple sclerosis	**-1.8 (-2.5 to -1.1)**	**endorsed by all panelists**	**-1.9 (-2.6 to -1.2)**
Paralysis	**-0.9 (-1.5 to -0.3)**	**-1.4 (-2.9 to 0.01)**	**-0.9 (-1.4 to -0.4)**
Transient ischaemic attack	**-0.1 (-0.6 to 0.4)**	**-2.2 (-4.2 to -0.2)**	**-0.4 (-0.8 to 0.1)**
Chronic primary pain	0.2 (-0.3 to 0.8)*	0.003 (-1.0 to 1.1)*	0.2 (-0.2 to 0.7)*
Peripheral neuropathy	0.5 (-0.02 to 1.0)*	**-2.2 (-4.2 to -0.2)**	0.1 (-0.3 to 0.6)*
Sleep apnoea	0.5 (-0.3 to 1.0)*	0.6 (-0.2 to 1.4)	0.6 (0.2 to 1.0)
Migraine	0.5 (0.1 to 1.1)*	1.3 (0.5 to 2.1)	0.8 (0.4 to 1.2)
**Metabolic and endocrine disorders**			
Diabetes	**-3.4 (-4.9 to -2.0)**	**endorsed by all panelists**	**-3.5 (-4.9 to -2.1)**
Cystic fibrosis	**-1.2 (-1.8 to -0.6)**	**-2.2 (-4.2 to -0.2)**	**-1.3 (-1.9 to -0.7)**
Thyroid disorders	**-0.3 (-0.9 to 0.3)**	**-0.7 (-1.8 to 0.4)**	**-0.3 (-0.8 to 0.2)**
Addison’s disease	**-0.6 (-1.3 to 0.1)**	**-1.7 (-3.8 to 0.3)**	**-0.8 (-1.4 to 0.1)**
Malnutrition	1.4 (0.9 to 1.8)	**-0.1 (-1.0 to 0.9)**	1.1 (0.7 to 1.5)
**Musculoskeletal diseases**			
Connective tissue disease	**-3.4 (-4.8 to -2.0)**	**-1.4 (-2.9 to 0.01)**	**-2.3 (-3.1 to -1.5)**
Osteoarthritis	**-0.5 (-1.0 to 0.1)**	**-0.7 (-1.8 to 0.4)**	**-0.5 (-1.0 to 0.04)**
Long-term musculoskeletal impairment due to injuries	0.01 (-0.5 to 0.6)*	0.6 (-0.3 to 1.6)	0.2 (-0.3 to 0.7)*
Gout	0.2 (-0.3 to 0.7)*	0.8 (-0.009 to 1.6)	0.4 (0.01 to 0.9)*
Chronic low back pain	0.5 (-0.1 to 1.0)*	1.6 (0.7 to 2.4)	0.8 (0.3 to 1.2)
Osteoporosis	0.6 (0.2 to 1.1)	**-0.2 (-1.1 to 0.8)**	0.5 (0.08 to 0.9)*
**Respiratory diseases**			
Chronic obstructive pulmonary disease	**-3.4 (-4.8 to -2.0)**	**-2.2 (-4.2 to -0.2)**	**-3.1 (-4.2 to -1.9)**
Asthma	**-1.5 (-2.2 to -0.9)**	**0.2 (-1.1 to 0.8)**	**-1.1 (-1.7 to -0.6)**
Bronchiectasis	**-0.7 (-1.3 to 0.001)**	**-1.1 (-2.6 to 0.4)**	**-0.7 (-1.3 to -0.1)**
Post-acute COVID-19	0.4 (-0.2 to 0.9)*	**-0.6 (-1.9 to 0.6)**	0.2 (-0.3 to 0.7)*
Chronic/Allergic rhinitis	1.6 (1.2 to 2.0)	0.6 (-0.2 to 1.4)	1.4 (1.0 to 1.8)

**Infectious diseases**			
HIV/AIDS	**-1.7 (-2.5 to -1.0)**	**-1.0 (-2.2 to 0.2)**	**-1.5 (-2.2 to -0.9)**
Chronic Lyme disease	**-0.1 (0.7 to 0.5)**	0.2 (-0.9 to 1.2)*	**-0.03 (-0.5 to 0.5)**
Tuberculosis	0.2 (-0.3 to 0.7)*	**-1.0 (-2.2 to 0.2)**	0.07 (-0.4 to 0.5)*
**Digestive diseases**			
Chronic liver diseases	**-3.4 (-4.8 to -2.0)**	**endorsed by all panelists**	**-3.5 (-4.9 to -2.1)**
Inflammatory bowel disease	**-1.8 (-2.5 to -1.0)**	**endorsed by all panelists**	**-1.9 (-2.6 to -1.2)**
Pancreatic disease	**-0.4 (-1.0 to 0.1)**	**-2.2 (-4.2 to -0.2)**	**-0.7 (-1.2 to -0.1)**
Peptic ulcer	0.4 (-1.0 to 0.9)*	**-0.4 (-1.4 to 0.6)**	0.3 (-0.2 to 0.7)*
Irritable bowel syndrome	0.9 (0.4 to 1.4)	0.1 (-0.8 to 1.0)*	0.8 (0.4 to 1.2)
Gall bladder disorders	1.1 (0.6 to 1.5)	0.5 (-0.3 to 1.4)*	1.0 (0.6 to 1.4)
Gastroesophageal reflux	1.3 (0.9 to 1.7)	1.3 (0.5 to 2.1)	1.3 (0.9 to 1.7)
Diverticulosis	1.8 (1.4 to 2.3)	0.1 (-0.8 to 1.0)*	1.5 (1.1 to 1.9)
**Urogenital diseases**			
Chronic kidney disease	**-2.7 (-3.7 to -1.7)**	**endorsed by all panelists**	**-2.8 (-3.8 to -1.8)**
End-stage kidney disease	**-1.9 (-2.6 to -1.1)**	**endorsed by all panelists**	**-2.0 (-2.7 to -1.2)**
Endometriosis	0.3 (-0.2 to 0.8)*	**-0.7 (-2.0 to 0.5)**	0.1 (-0.3 to 0.6)*
Chronic urinary tract infection	0.5 (-0.05 to 1.0)*	**-0.6 (-1.7 to 0.4)**	0.3 (-0.1 to 0.8)*
Polycystic ovary syndrome	0.9 (0.5 to 1.4)	**-0.6 (-1.7 to 0.5)**	0.7 (0.2 to 1.1)
Urinary incontinence	0.9 (0.4 to 1.3)	0.6 (-0.2 to 1.4)	0.9 (0.5 to 1.3)
Uterine (womb) problems	1.1 (0.6 to 1.5)	**-0.2 (-1.1 to 0.8)**	0.8 (0.4 to 1.2)
Prostatic hypertrophy	1.1 (0.7 to 1.6)	**-1.0 (-2.2 to 0.2)**	0.7 (0.3 to 1.2)
Kidney or bladder stones	1.5 (1.1 to 2.0)	0.1 (-0.8 to 1.0)*	1.2 (0.8 to 1.6)
Sexual dysfunction	2.0 (1.5 to 2.4)	2.3 (1.5 to 3.2)	2.1 (1.7 to 2.5)
Infertility	2.7 (2.2 to 3.2)	2.8 (1.9 to 3.7)	2.7 (2.3 to 3.1)
**Skin disorders**			
Psoriasis	0.4 (-0.05 to 0.9)*	0.8 (-0.009 to 1.6)	0.6 (0.2 to 1.0)
Eczema	1.6 (1.1 to 2.0)	1.3 (0.5 to 2.1)	1.5 (1.2 to 1.9)
Chronic urticarial	1.4 (0.9 to 1.8)	2.1 (1.3 to 2.9)	1.6 (1.2 to 2.0)
**Eye diseases**			
Vision impairment that cannot be corrected with glasses	0.05 (-0.5 to -0.6)*	0.3 (-0.6 to 1.1)*	0.1 (-0.3 to 0.6)*
Glaucoma	0.7 (0.2 to 1.1)	0.5 (-0.3 to 1.3)*	0.7 (0.3 to 1.1)
Cataract	1.0 (0.6 to 1.5)	1.4 (0.7 to 2.2)	1.2 (0.8 to 1.6)
**ENT disorders**			
Hearing impairment that cannot be corrected with hearing aids	0.4 (-0.1 to -0.9)*	0.3 (-0.6 to 1.1)*	0.4 (-0.02 to 0.8)*
Meniere's disease	0.6 (0.1 to 1.1)	0.4 (-0.4 to 1.3)*	0.5 (0.2 to 1.0)*
Chronic sinusitis	1.6 (1.1 to 2.0)	1.8 (1.0 to 2.5)	1.7 (1.3 to 2.0)
**Oral disorders**			
Chronic gum disease	1.3 (0.9 to 1.7)	2.3 (1.4 to 3.1)	2.0 (1.6 to 2.4)
Edentulism	1.9 (1.5 to 2.3)	2.4 (1.6 to 3.3)	2.3 (1.9 to 2.7)
**Congenital and chromosomal abnormalities**	0.5 (-0.003 to 1.0)*	**-1.0 (-2.2 to 0.2)**	0.2 (-0.2 to 0.7)*
**Risk factors/Symptoms/ Behaviour**			
Obesity	1.0 (0.5 to 1.4)	1.0 (0.2 to 1.7)	1.0 (0.6 to 1.4)
Physical disability	1.5 (1.1 to 2.0)	0.3 (-0.6 to 1.1)*	1.3 (0.9 to 1.6)

Smoking	2.5 (2.0 to 2.9)	1.9 (1.1 to 2.7)	2.4 (2.0 to 2.8)
Social deprivation or poverty	2.5 (2.1 to 3.0)	1.7 (0.9 to 2.5)	2.4 (2.0 to 2.8)
Post-sepsis syndrome	2.8 (2.3 to 3.3)	1.4 (0.5 to 2.2)	2.5 (2.1 to 2.9)
Treatment burden	2.8 (2.4 to 3.3)	1.5 (0.4 to 2.3)	2.5 (2.2 to 2.9)
Dizziness	2.9 (2.4 to 3.4)	2.4 (1.6 to 3.3)	2.9 (2.4 to 3.4)
Side effects of medicines	3.0 (2.5 to 3.4)	1.5 (0.4 to 2.3)	2.8 (2.4 to 3.2)
Chronic cough	3.1 (2.6 to 3.6)	2.3 (1.4 to 3.1)	2.9 (2.5 to 3.3)
Sedentary lifestyle	3.8 (3.3 to 4.4)	2.3 (1.4 to 3.1)	3.4 (3.0 to 3.9)

Lower difficulty parameter estimates indicate more frequent endorsement of items. Here we classified the numbers to three levels— ≤0 (in bold) indicates strongly endorsed, 0-0.5 indicates endorsed (*), and >0.5 indicates infrequently endorsed [5, 6]

References

1. Boone, W.J., *Rasch Analysis for Instrument Development: Why, When, and How?* CBE life sciences education, 2016. **15**(4): p. 1-7.
2. Ptukhin, Y. and Y. Sheng, *Comparing two maximum likelihood algorithms for mixture Rasch models.* Behaviormetrika, 2019. **46**(1): p. 101-119.
3. Khine, M.S., *Rasch measurement applications in quantitative educational research*. 2020, Singapore: Springer.
4. Hagquist, C. and D. Andrich, *Recent advances in analysis of differential item functioning in health research using the Rasch model.* Health and Quality of Life Outcomes, 2017. **15**(181): p. 1-8.
5. Hays, R.D., L.S. Morales, and S.P. Reise, *Item response theory and health outcomes measurement in the 21st century.* Medical care, 2000. **38**(9): p. II28-II42.
6. Verhelst, N.D., *Item response theory*, in *Relating Language examinations to the Common European Framework of Reference for Languages: learning, teaching, assessment* 2004, Language Policy Division: Strasbourg.
